# Neuropathic pain; what we know and what we should do about it

**DOI:** 10.3389/fpain.2023.1220034

**Published:** 2023-09-22

**Authors:** Peter A. Smith

**Affiliations:** Neuroscience and Mental Health Institute and Department of Pharmacology, University of Alberta, Edmonton, AB, Canada

**Keywords:** neurogenic neuroinflammation, allodynia, dorsal horn, dorsal root ganglia, neuropathy, nerve injury, neuroinflammation, neuroimmunology

## Abstract

Neuropathic pain can result from injury to, or disease of the nervous system. It is notoriously difficult to treat. Peripheral nerve injury promotes Schwann cell activation and invasion of immunocompetent cells into the site of injury, spinal cord and higher sensory structures such as thalamus and cingulate and sensory cortices. Various cytokines, chemokines, growth factors, monoamines and neuropeptides effect two-way signalling between neurons, glia and immune cells. This promotes sustained hyperexcitability and spontaneous activity in primary afferents that is crucial for onset and persistence of pain as well as misprocessing of sensory information in the spinal cord and supraspinal structures. Much of the current understanding of pain aetiology and identification of drug targets derives from studies of the consequences of peripheral nerve injury in rodent models. Although a vast amount of information has been forthcoming, the translation of this information into the clinical arena has been minimal. Few, if any, major therapeutic approaches have appeared since the mid 1990's. This may reflect failure to recognise differences in pain processing in males vs. females, differences in cellular responses to different types of injury and differences in pain processing in humans vs. animals. Basic science and clinical approaches which seek to bridge this knowledge gap include better assessment of pain in animal models, use of pain models which better emulate human disease, and stratification of human pain phenotypes according to quantitative assessment of signs and symptoms of disease. This can lead to more personalized and effective treatments for individual patients. Significance statement: There is an urgent need to find new treatments for neuropathic pain. Although classical animal models have revealed essential features of pain aetiology such as peripheral and central sensitization and some of the molecular and cellular mechanisms involved, they do not adequately model the multiplicity of disease states or injuries that may bring forth neuropathic pain in the clinic. This review seeks to integrate information from the multiplicity of disciplines that seek to understand neuropathic pain; including immunology, cell biology, electrophysiology and biophysics, anatomy, cell biology, neurology, molecular biology, pharmacology and behavioral science. Beyond this, it underlines ongoing refinements in basic science and clinical practice that will engender improved approaches to pain management.

## Introduction

Diseases or lesions that affect the somatosensory system often elicit long lasting neuropathic pain. The signs and symptoms in each individual depend strongly on the nature of the injury as well as their sex, age, ethnicity, genetic predisposition, intestinal microbiome, possible exposure to prior neonatal injury, personality and cultural and environmental factors (1–11). The predominant signs and symptoms include bouts of spontaneous “electric shock-like” pain, the generation of pain by non-noxious touch or cold (mechanical or thermal allodynia) as well as hyperalgesia and sensory disturbances. The latter may present as paresthesias, described as a crawling or pricking sensation or tingling (12). Some patients experience *anesthesia dolorosa* where the site of injury is painful yet insensitive to touch (13). Others experience the persistent burning pain of causalgia (14). Neuropathic pain is often intractable (15), insensitive to the actions of NSAID's and resistant to the actions of opioids (16, 17). Unlike nociceptive pain, which alerts and protects an individual from actual or potential tissue injury, neuropathic pain persists long after damaged tissue has healed and recovered (18, 19). Since it appears to serve no obvious biological purpose, neuropathic pain has long been assumed to be maladaptive (20–23).

Maladaptive or not, neuropathic pain afflicts 5%–10% of the world's population (15, 24, 25) and frequently presents with co-morbidities such as anxiety, depression, irritability and sleep disorders (12, 26).

Such high prevalence reflects the association of neuropathic pain with a broad range of injuries and/or maladies (12, 14, 27). These not only include peripheral nerve trauma (13, 23, 28, 29), amputation (30), brain trauma (14, 20) or spinal cord injury (31, 32). Neuropathic pain may also occur as a result of multiple sclerosis (33, 34), stroke (14, 35), fibromyalgia (36, 37), small fiber neuropathy (38), post herpetic or trigeminal neuralgia (14, 39), migraine (40), osteoarthritis (41, 42), complex regional pain syndromes I and II (43, 44), rheumatoid arthritis (45), painful diabetic neuropathy (46, 47), autoimmune disease (48), viral infections such as HIV (49–51) or COVID 19 (52) and neuropathies associated with cancer *per se* (47) and/or chemotherapy (53–55). Neuropathic pain is also prevalent in individuals afflicted with posttraumatic stress disorder (56) and is a positive sign of rare yet debilitating Na^+^ channelopathies (57–59). In view of the prevalence of this frequently intractable condition, there is a clear and increasingly urgent need to develop new therapeutic approaches (14, 17, 22).

Despite the heterogeneity of the patient population and the association of neuropathic pain with multiple clinical conditions (27), much of the present understanding derives from studies using peripheral nerve injury models in rodents (47, 60, 61). Frequently used models include chronic constriction injury of the sciatic nerve (CCI), spared nerve injury (SNI) of sciatic nerve branches, spinal nerve ligation (SNL), chronic constriction of dorsal root ganglia (CCDRG) and partial nerve ligation or the Seltzer model (PNL) (60–63). This multidisciplinary review will present a synopsis of these findings showing how they have led to a very general understanding of pain aetiology and to the identification of numerous potential drug targets. Despite this, translation between the laboratory and clinic has met with very limited success (10, 25, 64). The extent of the misalignment between preclinical pain research and the clinical population is becoming increasingly clear (25, 65). In view of this, clinical and basic science strategies that seek to bridge this knowledge gap will be presented.

## Peripheral nerve injury and the generation and release of primary inflammatory mediators

Peripheral nerve injury capable of causing neuropathic pain does not usually kill peripheral neurons (66). It does however promote Wallerian degeneration of severed axons. This is driven by activation of Schwann cells, fibroblasts, mast cells, keratinocytes, epithelial cells at the site of injury as well as neutrophil, macrophage and T- lymphocyte invasion. This is accompanied by activation of satellite glial cells and resident macrophages within the dorsal root ganglia (DRG) (18, 67–72).

**Figure 1:**
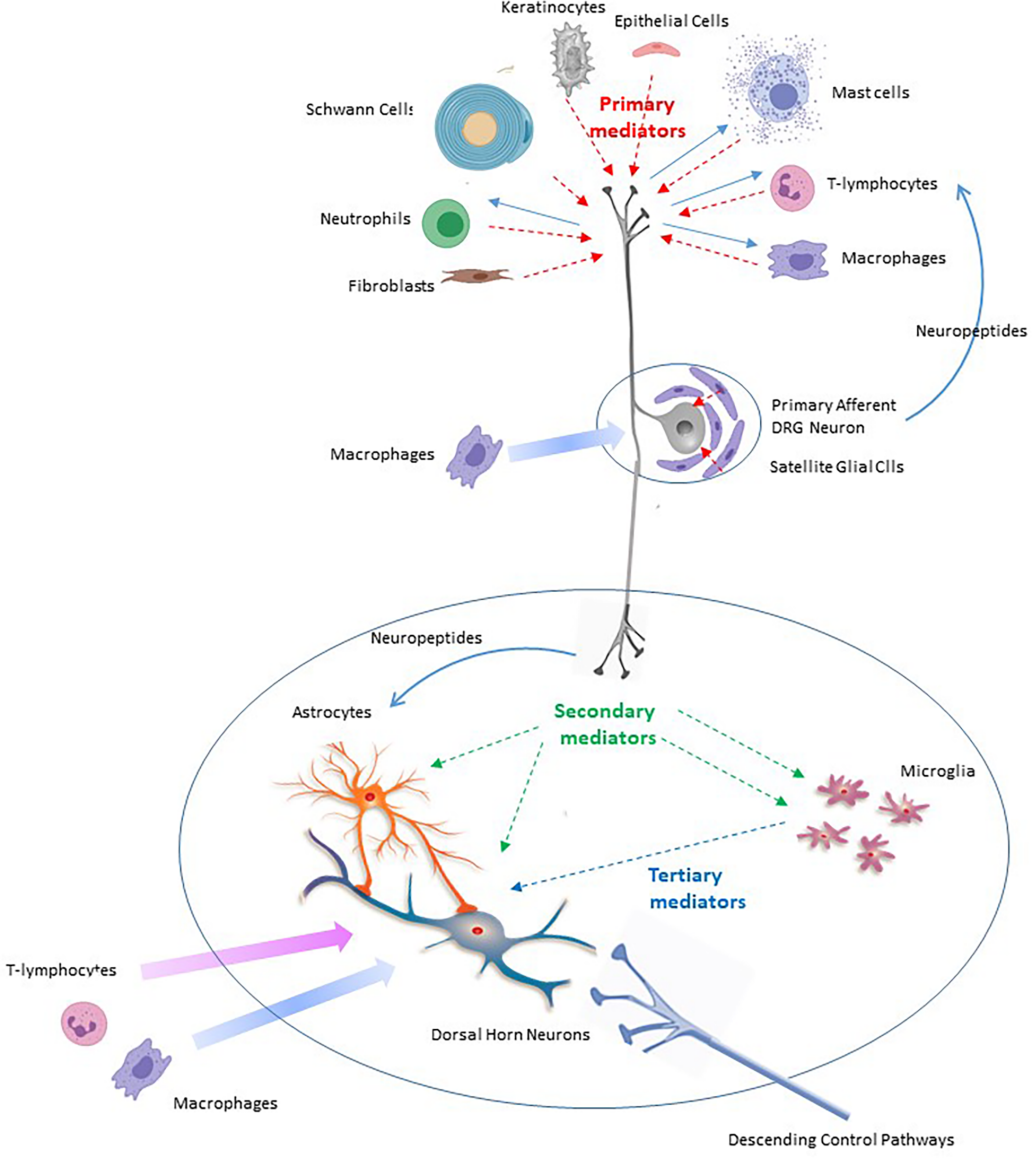
Scheme to illustrate the roles of primary, secondary and tertiary mediators in the onset and maintenance of neuropathic pain in response to peripheral nerve injury.

Once activated, each of these immunocompetent cell types generate and release an assortment of pro-inflammatory **primary mediators** (Table 1 and Figure 1). These include interleukins 1α, 1β, 6, 8, 15, 17 and 18 (IL-1α, IL-1β, IL-6, IL-8, IL-15, IL-17 and IL-18) (73–84), tumor necrosis factor *α* (TNF-α) (81, 85, 86), leukemia inhibitory factor (LIF) (87), oncostatin M (OSM) (88), nerve growth factor (NGF) (18, 89, 90), serotonin, histamine, and substance P (91–94), the secreted glycoproteins Wnt3a and Wnt5a (wingless-type mammary tumor virus integration site family, members 3A and 5A) (95, 96) and the chemokines CCL-2 (97–99), CXCL-1 (70, 100), CXCL-4 (101) and CXCL-12 (102–104) (Table 1). Generation of primary mediators is accompanied by the production of reactive oxygen and nitrogen species (ROS and NOS) such as peroxynitrite and hydrogen peroxide (105–107). These damage mitochondria causing them to leak ROS and components of damage associated molecular patterns (DAMPs) (27, 108). Mitochondrial dysfunction is emerging as a key process in pain etiology (55, 109).

**Table 1:** List of primary, secondary and tertiary mediators involved in the onset and maintenance of neuropathic pain in response to peripheral nerve injury.

Primary Mediators	Secondary Mediators	Tertiary Mediators	Receptor
Released from peripheral immunocompetent cells following injury and acting on DRG neurons	Released in the spinal cord and affecting the properties of microglia and astrocytes	Released from microglia and astrocytes and acting upon dorsal horn neurons	
*** ***	CSF-1 (68, 110–113)		CSF-1r
	CCL-21 (114–116)		CXCR-3
		BDNF (117–123)	TrkB
	Wnt5a (124)		Human frizzled-5 (hFz5)
	FKN (CX3CL-1) (125–127)		CX3CR-1
CCL-2 (MCP-1) (97, 128, 129)			CCR-2, CCR-4
CXCL-1 (70, 100)			CKCR-2
CXCL-12 (102–104)	CXCL-12 (102–104)		CXCR-4
CXCL-4 (101, 104, 130)			CXCR-4
Histamine (92, 93)			H3, H4
IFN–γ (131)	IFN–γ (132)	IFN–γ	IFN–γ -R
IL-17 (84)		IL-17 (133, 134)	IL-17R
IL-1β (76, 135–140)		IL-1β (18, 70, 75, 141–144)	IL-1R
LIF (145)			LIF-R
NGF (89)			TrkA
Serotonin (146, 147)			5-HT4
Oncostatin M (88)			Oncostatin M Receptor
Substance P(94, 148)			NK1-R
TNF-α (or β) (85, 149)		TNF-α or β (68, 85, 150–152)	TNFR-1, TNF-R2
Wnt3a (95, 153)			Human frizzled-3 (hFz3)?

Whilst some primary mediators have predominantly localized actions, others are released into the systemic circulation (38, 82).

### Generation, release and processing of IL-1β and TNF-α in damaged peripheral nerves

Schwann cell derived IL-1α and TNF-α serve as very early mediators in the response of axons to injury. They recruit macrophages and initiate molecular and cellular events in Wallarian degeneration such as the production of additional cytokines and NGF (81).

Generation of IL-1β is brought about by activation of the Nod-like receptor family pyrin domain containing 3 (NLRP3) inflammasone (33, 154–156). NLRP3 is activated following the release of DAMPs and their interaction with pattern recognition receptors (PRRs) (157), such as Toll-like receptors (TLRs) (158, 159) and with purinergic P2X7 receptors (159). IL-1β is released as a pro-protein and processed into its mature bioactive form by caspase-1 (160) or by metalloproteases 2 and 9 (MMP2 and MMP9) (161). Release of IL-1β from macrophages, dendritic cells and neutrophils, may be brought about via the formation of gasdermin D pores in the cell membrane (160, 162, 163). Alternatively IL-1β release may involve its exocytosis via panexin channels (164).

Metalloproteases also cleave the membrane bound form of TNF-α into the mature 17-kDa form (165). This and their ability to also cleave IL-1β and to produce pain when administered intrathecally has led to the suggestion that MMP antagonists may be useful in pain management (161). There are however no reports of use of metalloprotease inhibitors in pain management in the clinic. This may be due, in part, to the observation that activated MMP2 and MMP9 cleave the mature form of NGF into biologically inactive products (166). The effect of MMP blockade here would be to preserve the presence of pro-inflammatory NGF. This possible proinflammatory action of MMP blockers would tend to restrain any anti-inflammatory/analgesic action.

## Neuroinflammation and the actions of primary mediators on primary afferent neurons

The overall response of neuronal tissue to inflammatory mediators is described as “neuroinflammation” (167–170). It is characterized by glial cell proliferation and modulation of their phenotype as well as increased neuronal activity. Although the same mediators are responsible for both phenomena, neuroinflammation should not be confused with classical inflammation of whole tissues which is associated with redness (rubor), swelling (tumour), heat (calor) and pain (dolor).

Administration of primary mediators *in vivo* promotes pain in uninjured animals (70, 74, 135, 165) and perturbation of their actions in nerve-injured animals abrogates or attenuates signs of neuropathic pain (73, 75, 76, 79, 89, 92, 93, 98, 101, 104, 114, 171–173).

Primary mediators such as IL-1β, IL-17, TNF-α, CCL-2, CXCL-12 or type 1 interferons (IFN-1) (131) interact with their cognate receptors on primary afferent neurons to promote extensive changes in genes coding for chemokines, cytokines, eicosanoids, receptors, neuropeptides, signal transduction molecules, synaptic vesicle proteins and ion channels (174, 175). They also affect the expression of long non-coding RNA's (176) and microRNA's (mIR) (177–185). The latter post-transcriptionally regulate the protein expression of hundreds of genes in a sequence-specific manner (186–188) to orchestrate both immune and neuronal processes (189). The observation that extracellular release of mIRs from rodent DRG is increased after CCI (190) is consistent with their suggested role in pain etiology.

It should be noted that the actions of primary mediators are not restricted to peripheral nociceptors (136–138). Tactile information from fast conducting Aβ fibres is processed exclusively within the deep dorsal horn. After peripheral inflammation however, inhibitory spinal circuits are compromised so that innocuous tactile information finds its way to pain processing neurons in the *substantia gelatinosa* (191). In other words, types of afferent that do not convey pain under normal circumstances start to signal information that is interpreted as pain after nerve injury. This likely contributes to the phenomenon of allodynia. Actions of primary mediators on any type of sensory neuron may therefore have relevance to the onset of pain.

Primary mediators that enter the blood stream promote plasma extravasation and increased permeability of the blood-brain barrier (192) and the blood-nerve barrier in the periphery (193). This and the chemoattractant properties of mediators such as TNF-α (81) facilitate the continuing recruitment of immunocompetent macrophages, leucocytes and lymphocytes to the site of nerve injury as well as to the spinal cord, DRG and supra-spinal structures (70, 77, 78, 194, 195).

Although these findings point to numerous drug targets, clinical trials that involve the perturbation of the action of chemokines, cytokines and other primary mediators have failed to bring forth new and effective therapeutic entities (17, 141, 196).

### Importance of primary afferent hyper-excitability and actions of primary mediators on ion channels in peripheral neurons

Peripheral nerve injury, via the actions of primary mediators, leads to ectopic spontaneous activity in primary afferents that is crucial for the onset and persistence of neuropathic pain in humans and signs of such pain in rodent models (19, 30, 35, 197–206). Thus, suppression of aberrant peripheral nerve activity in animal models *in vivo* by either optogenetic or pharmacological methodologies (205, 207) leads to attenuation of hyperalgesia and abatement of injury-induced allodynia.

In general, peripheral nerve injury, by the action of primary mediators, decreases K^+^ channel function and increases that of voltage-gated Na^+^ and Ca^2+^ channels, TRP channels and HCN channels in DRG neurons (208–213). Injury-induced changes in ion channels can also provoke bursting activity in sensory neurons (214) that may relate to release of ATP and its interaction with P2X3 receptors at the site of injury and the initiation synchronous oscillations in primary afferents (206). In addition, altered excitability may be a consequence of mitochondrial dysfunction and chronic energy deficit (215).

Peripheral activity after injury may affect the whole somatosensory system. It may provoke enduring low frequency cortical oscillations and synaptic remodeling in S1 somatosensory cortex as well as for inducing animals' pain-like behaviors (206). This is supported by the observation that enhancing the synchrony of DRG neuronal activity causes synaptic changes in S1 and pain-like behaviors similar to those seen after spared nerve injury (SNI).

An overview of the actions of IL-1β, TNF-α, Wnt ligands, chemokines and other primary mediators on peripheral neurons is presented in the succeeding sections.

### Effects of IL-1β on ion channels in peripheral neurons

Acute application of IL-1β increases the excitability of DRG neurons by relieving slow inactivation of tetrodotoxin (TTX)-resistant voltage-gated sodium channels (135). IL-1β levels peak at 1 d after injury and remain elevated for ∼7 d (139) and investigations of its longer term actions following 5 d–6 d exposure reveal different effects on different neuronal subpopulations (136). These are observed at remarkably low concentrations (216).

The long term effects of IL-1β on small IB_4_ -positive neurons (most of which are non-peptidergic, low threshold mechanoceptors) include a reversible increase in action potential (AP) amplitude as a result of increased tetrodotoxin (TTX)-sensitive Na^+^ current and an irreversible increase in AP duration as result of decreased Ca^2+^- sensitive K^+^ conductance (138).

The effects of IL-1β on medium sized neurons, which are the cell bodies of Aδ fibres, are dominated by decreases in K^+^ currents (137). Although the precise ionic mechanisms differ, IL-1β increases the excitability of both small-diameter IB_4_-positive neurons and medium-diameter neurons. By contrast, large neurons which are the cell bodies of fast conducting Aβ fibres and IB_4_-negative neurons, which are predominantly peptidergic nociceptors, are little affected (136).

### Effects of TNF-α on ion channels in peripheral neurons

Macrophage and Schwann cell derived TNF-α is upregulated at the site of injury following CCI (85) and its peripheral application promotes ectopic activity in nociceptors *in vivo* (217). This effect is enhanced after SNL injury (173). Microinjection of TNF-α lowers mechanical pain threshold in nerve-injured animals in a similar fashion to IL-1β. Most actions of TNF-α in DRG involve modifications of Na^+^ channel function (218) rather than effects on K^+^ channels (210). For example, TNF-α upregulates Na_v_1.7 (219) as well as slow persistent TTX-resistant Na^+^ channel currents (149).

### Effects of Wnt ligands on excitability of peripheral neurons

Intraplantar injection of Wnt3a promotes mechanical hypersensitivity and thermal hyperalgesia in uninjured animals. It also upregulates the ionotropic ATP receptor P2X3 as well as TRPA1 receptor channels. P2X3 receptors may be activated by the passive release of ATP from damaged cells leading to increased sensory neuron excitability (153). Wnt3a also stimulates production of TNF-α and IL-18, thereby augmenting the overall inflammatory response

### Effects of chemokines on peripheral neuron excitability

Several chemokines excite DRG neurons (97, 220).

CCL-2 signals through CCR-2 to increase nociceptor excitability (97, 128, 221, 222). Its effectiveness is increased after DRG compression (CCDRG) (223). CCL-2 is expressed by DRG neurons where it is packaged into large dense-core vesicles. Release of vesicles can be induced by depolarization in a Ca^2+^-dependent manner (224). This autocrine function could thereby amplify injury-induced excitatory processes evoked in DRG.

CXCL-12 signalling through its cognate receptor, CXCR-4 increases excitability of Na_v_1.8-positive DRG neurons and this plays a role in the generation of mechanical allodynia as well as small-fiber degeneration in a mouse model of peripheral diabetic neuropathy (101). CXCL-12 and CXCR-4 are upregulated after CCDRG. In addition, intrathecal injection of a CXCL-12 antagonist or a CXCL-12 neutralizing antibody reverse allodynia after SNI or CCDRG (103, 104, 130). These findings suggest that peripheral CXCL-12/CXCR-4 signaling contributes to pain after damage to the DRG *per se* (104).

### Effects of prostaglandins, histamine and serotonin on ion channels in peripheral neurons

In addition to secreted proteins, chemokines, cytokines and growth factors, several small molecules produced at the site of injury act as primary mediators. These include prostaglandin E2, bradykinin, serotonin (146) and histamine (92); all of which increase the excitability of DRG neurons (131, 147). Actions of both serotonin and PGE2 involve augmentation of TTX-resistant I_Na_ in nociceptors (225).

### Peripheral neuron ion channels as therapeutic targets

As already mentioned, manipulation of the actions of cytokines, chemokines or other primary mediators has so far failed to bring forth any promising therapeutic approaches. On the other hand, the crucial role of primary afferent hyperexcitability and spontaneous activity in pain etiology (30, 199, 202, 205) draws attention to the potential use of ion channels as therapeutic targets (59, 208–210, 226).

### Voltage-gated K^+^ channels

DRG neurons express a variety of K^+^ channel subtypes including delayed rectifiers (K_v_1.1, 1.2), A-channels (K_v_1.4, 3.3, 3.4, 4.1, 4.2, and 4.3), KCNQ or M-channels (K_v_7.2, 7.3, 7.4, and 7.5), ATP-sensitive K^+^ channels (K_IR_6.2), Ca^2+^-activated K^+^ channels (K_Ca_1.1, 2.1, 2.2, 2.3, and 3.1), Na^+ ^-activated K^+^ channels (K_Ca_4.1and 4.2) and two pore domain leak channels (K2p; TWIK related channels). These channel subtypes are preferentially and differentially expressed in various neuronal subpopulations and attempts to restore K^+^ channel function have involved the use of channel activators (210). Although K_v_7 activators are quite effective in rodent models (227, 228),, the anticonvulsant, retigabine failed to reach its efficacy endpoint in a trial for post herpetic neuralgia (17).. Nevertheless, as will be outlined below, better phenotypical stratification of patents into clusters on the basis of quantitative measurements of their pathophysiology may reveal clinical efficacy of drugs that failed to demonstrate effectiveness in large groups of patients (8). In the case of K^+^ channel activators, over 200 new molecules are currently under investigation (227).

Mechanisms that control K^+^ channel expression and function may present additional therapeutic targets. For example, the expression of K_v_7.2, K_v_1.4 and K_Ca_1.1 is controlled by the histone methyltranferase G9a (229). Pharmacological inhibition of G9a attenuates neuropathic pain in rodent models (230, 231). Although there is considerable interest in developing histone methyltransferase inhibitors in cancer treatment (232), none have been examined for treatment of neuropathic pain.

### Voltage-gated Na^+^ channels

A variety of Na^+^ channel blockers show promise as therapeutic agents; inhibition of Na_v_1.7, 1.8 or 1.9 seems particularly effective (208, 233, 234). Because it is not found to any great extent in non-neuronal vital tissue such as heart or skeletal muscle, Na_v_1.7 represents an especially attractive target for therapeutic manipulation (59, 208). Indeed, some level of success has been realized in phase II clinical trials for trigeminal and diabetic neuralgia with the Na_v_1.7 blocker, vixotrigine (235, 236) but phase III trials remain at the planning stage (235).

Expression of Na_v_1.8 in DRG neurons is controlled by NGF (237) and the NGF binding antibody tanezumab is effective in various human pain states (238). Small molecule, peripherally-acting TrkA inhibitors have also been identified (239–241).

### High voltage-activated Ca^2+^ channels

DRG neurons express high voltage-activated (HVA) Ca^2+^ channels; Ca_v_2.2 (N-type) as Ca_v_2.1 (P/Q-type) and Ca_v_1.2 (L-type) (242). Low voltage-activated (LVA) channels (T-type) are also present, notably Ca_v_3.2 and 3.3 (243–245).

Because Ca_v_2.1 (P/Q type) and Ca_v_2.2 (N-type) Ca^2+^ channels contain a synaptic protein interaction site (246) they are closely associated with the synaptic vesicles that govern neurotransmitter release. In view of this, the role of Ca^2+^ channels in controlling neuronal excitability and reports of upregulation of both HVA and LVA Ca^2+^ channels by injury (247–249), Ca^2+^ channels emerge as an important therapeutic target for pain management (208, 209, 226, 250–252). This potential has been realized by the use of the of N-type Ca^2+^ channel blocker ziconotide as a last resort for pain that is refractory to all other treatments (253). The drawback is that ziconotide needs to be delivered directly to the spinal cord via the intrathecal route (254). In view of this, there is strong interest in developing orally effective N-channel blockers (209, 226, 251, 253) and although several promising agents have appeared in the last five years, the ubiquitous distribution of N-type channels throughout the nervous system means that side effects of such agents may present a serious barrier to drug development.

The function of N-type Ca^2+^ channels is modulated by G_i/o_ coupled agonists (255, 256) but the clinical efficacy of the α2-adrenoceptor agonist, clonidine is limited to subsets of patients within the postherpetic neuralgia, complex regional pain syndrome or diabetic neuropathy cohorts (257). Nevertheless, this documented efficacy of clonidine has led to an extensive *in silico* modelling study. Compounds with nanomolar affinities for the α2a-adrenoceptors and limited ability to recruit arrestin *β* have been identified and tested in animal models where they behave as non-sedating, orally effective agents that attenuate signs of neuropathic, inflammatory and acute pain (258). The potent α2-adrenoceptor agonist, xylazine has been available for over 30 years, but its use has been restricted to pain management in veterinary medicine as it promotes severe hypotension and dangerous bradycardia in humans (259). It also has documented abuse potential (260).

In addition, the therapeutically important gabapentinoids (16, 261) modulate HVA Ca^2+^ channel function by binding to their α2δ–1 regulatory subunits (262). Gabapentinoids may antagonise the actions of the endogenous ligand thrombospondin (263). This means that perturbation of thrombospondin expression and/or function may present a novel therapeutic route to pain management. The α2δ–1 subunit plays a major role in Ca^2+^ channel trafficking, expression and function (22, 248, 264, 265) and deletion of the α2δ–1 gene delays development of mechanical hypersensitivity that follows peripheral nerve damage (262). α2δ–1 is also implicated in controlling the expression of Ca^2+^ permeable AMPA channels (266) and NMDA receptor channels (267). It is likely therefore that the therapeutic benefits of gabapentinoids involve interactions with several channel types.

### Low voltage-activated Ca^2+^ channels

LVA T-channels control nociceptor excitability (226, 268–270) and are involved in transmitter release from primary afferent terminals (271, 272). In some patients, gain of function mutations of Ca_v_3.1 contribute to trigeminal neuralgia (273). Peripheral nerve injury (CCI or diabetic neuropathy model) increases function of Ca_v_3.2, in rodent DRG neurons (249, 269) and specific knockdown of Ca_v_3.2 induces marked analgesia *in vivo* (270).

Although several small molecule Ca_v_3.2 blockers have shown promise in preclinical studies (274, 275) most have failed to exert a significant effects in cohorts of pain patients (208, 276). On the other hand, the high-affinity T-type channel blocker Z944 is especially effective in murine pain models and this may reflect selective blockade of Ca_v_3.1, Ca_v_3.2, and Ca_v_3.3 (277) in peripheral, spinal and thalamic neurons (278, 279). Preliminary results of phase 1 and phase 2 trials with Z944 also appear promising (280).

Some neuropathic pain patients respond favorably to cannabinoids (281) and this may be ascribed to inhibition of Ca_v_3.1 and/or Ca_v_3.2 Ca^2+^ channels (282, 283) as well as inhibition of N-type Ca^2+^ channels (284), augmentation of BK type K^+^ channel currents (285) and stabilization of an inactivated state of Na_v_1.8 channels (286). There has been considerable interest in NMP-7 and Compound 9 which affect Ca_v_3.2 channels by interactions CB1 and/or CB2 receptors. Although these compounds seem highly effective in animal models, they do not appear to have been tested in the clinic (287–289).

Rather than direct channel block or inhibition by the action of G_i/o_ coupled agonists, there is considerable interest in modulating Ca_v_3 channel activity by targeting the molecular mechanisms that regulate them.

**Figure 2:**
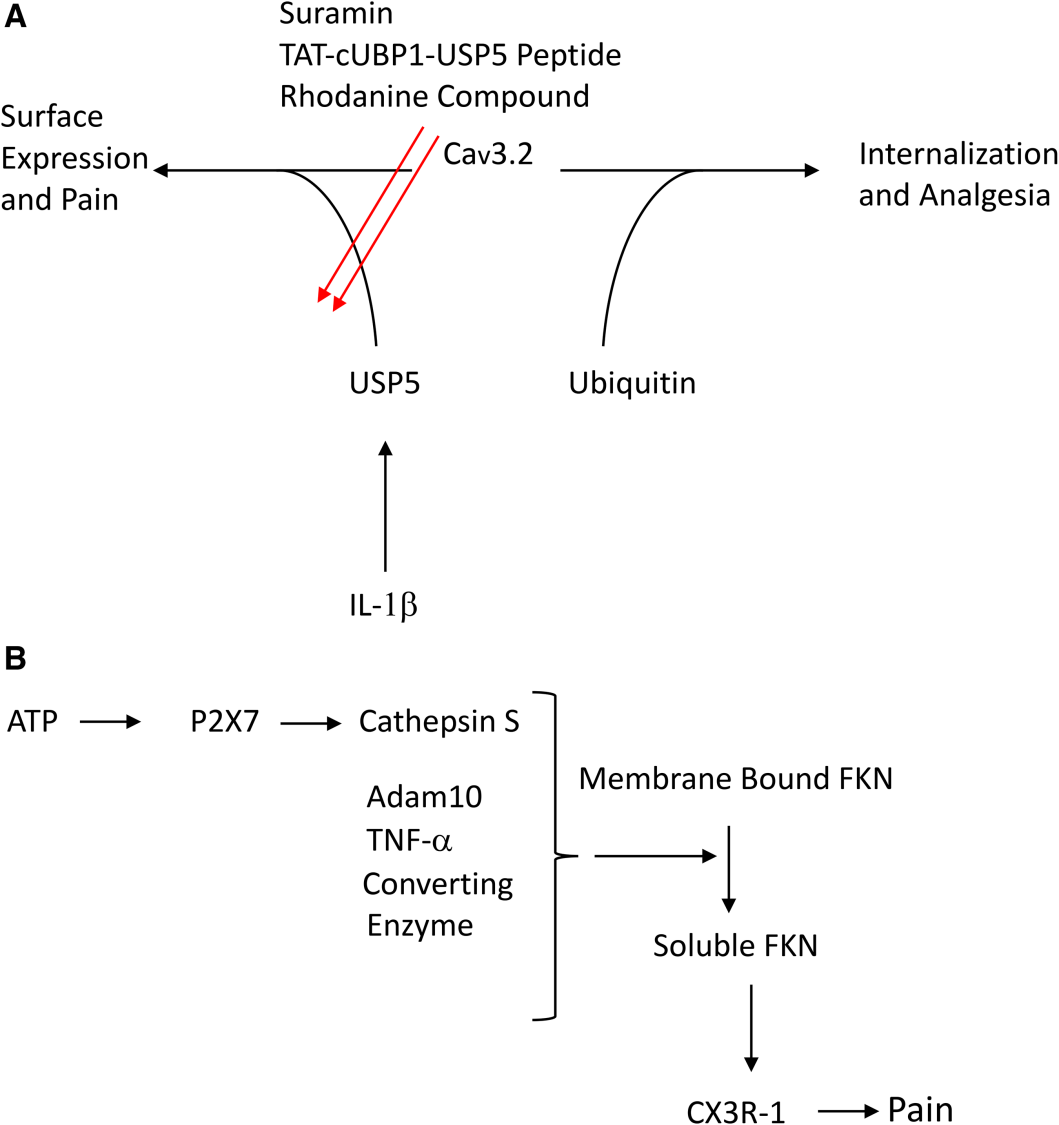
(**A**) Scheme to illustrate control of Ca_v_3.2 expression by ubiquitin and the deubiquitinase USP5 (**B**) scheme to illustrate role of cathespin and FKN in neuropathic pain.

For example, upregulation of the deubiquitinase, USP5 by IL-1β impairs Ca_v_3.2 ubiquitination thereby protecting it from proteasomal degradation and prolonging its surface expression (140, 272, 290, 291). USP5 knockdown thus increases Ca_v_3.2 ubiquitination, reduces its surface expression leading to reduction of Ca_v_3.2 whole-cell currents. This in turn, leads to attenuation of mechanical hypersensitivity in murine models of both inflammatory and neuropathic pain. As shown in Figure 2A, Ca_v_3.2/USP5 interactions are interrupted by a novel bioactive rhodanine compound (292), by the antiparasitic agent, suramin, and by a TAT-cUBP1-USP5 peptide. Each of these substances attenuate surface expression of Ca_v_3.2 and show analgesic activity in neuropathic and inflammatory pain models (292–294). These observations may lead to the development of new therapeutic approaches (292).

### HCN channels in primary afferent neurons

Nerve injury or long-term exposure to IL-1β increases HCN channel function in DRG (137, 295). This increase drives spontaneous activity (296, 297) and increases the release of neurotransmitter from primary afferents terminals (298, 299). HCN channel blockers thus supress signs of neuropathic pain in rodent models (300, 301) and selective deletion of HCN2 in nociceptive neurons prevents the development of neuropathic and inflammatory pain (296). Because the HCN2 channel subtype is mainly expressed in neurons as opposed to other excitable tissues (302), HCN2 blockers abrogate DRG hyperexcitability without affecting the HCN1 channels that control cardiac rhythmicity (303). In the clinic, the non-selective HCN blocker, ivabradine which is approved for treatment of heart failure, has a beneficial effect in painful diabetic neuropathy but only a weak effect in other forms of neuropathic pain (304).

### TRPV1 channels in peripheral neurons

TRPV1 receptor channels in nociceptors are upregulated following SNL injury (305) and sensitized by the action of inflammatory mediators (211). Unfortunately, the clinical effectiveness of TRPV1 blockers is limited by the presence of undesirable side effects (306). By contrast, transdermal patches containing a high concentration of the TRPV1 agonist, capsaicin have a role pain management (16). They are applied for 60 min in combination with regional anesthesia. This high level of capsaicin destroys the terminals of TRPV1 expressing nociceptors. This may include those that have sprouted into areas previously occupied by low threshold mechanoceptors (29).

In animal models, combining local anesthetics with capsaicin is especially effective in attenuating signs of pain. Molecules such as lidocaine pass freely through the pore of activated TRPV1 channels and thereby gain access to their intracellular binding site on the Na^+^ channel. The local anesthetic thus directly and selectively targets TRPV1 expressing nociceptors (307).

## Neuropeptides and their role in neuron-immune cell interactions

### Injury-induced changes in neuropeptide expression in primary afferent neurons

In addition to generation and release of inflammatory primary mediators, peripheral nerve injury alters expression of neuropeptides and their cognate receptors in primary afferent neurons (308–311). Neuronal activity promotes the release of neuropeptides such as CGRP and substance P from peripheral nerve endings, DRG cell bodies (312, 313) and primary afferent terminals (314, 315). They modulate sensory neuron activity by excitatory actions in DRG (148, 316, 317) and by their participation in axon reflexes at peripheral nerve endings (318, 319). Although increased effect of CGRP and substance P thus likely contributes to increased excitability and spontaneous activity of peripheral nerve, substance P antagonists are not effective in pain management in the clinical setting (320).

Erenumab, a monoclonal antibody raised against CGRP is available for the management of migraine (321) and recent evidence support the use of CGRP antagonists in the management of trigeminal neuralgia (321, 322). CGRP antagonism, both in the clinic and in animal models is less effective in males than in females (323, 324).

### Neuropeptides and other mediators of neurogenic neuroinflammation

Neuronal activity produces enduring changes in immune and glial cell function (18, 27, 68, 125, 325–327). This process has been termed neurogenic neuroinflammation (328, 329).

Injury-induced upregulation and release of neuropeptides is one of several mechanisms that effects transmission from neurons to glia and immune cells. For example, CGRP, substance P and vasoactive intestinal peptide (VIP) act on their cognate receptors on immune cells and vasculature to promote inflammation (318). CGRP regulates spinal microglial activation in a rodent model of neuropathic pain (330) and substance *P* regulates expression of IL-1β in keratinocytes (331).

Neuron-immune cell interactions can also be brought about by the synthesis and release of cytokines (110) and chemokines (224) from neurons *per se*.

### The immune reflex and control of neuro-immune interactions

By contrast with neurogenic neuroinflammation which is a consequence of injury, essentially the reverse effect; suppression of immune system activity by neuronal activity, characterises a well-defined immune reflex. This contributes to the resolution of inflammation following injury (327). The best characterized part of this reflex involves the vagal release of acetylcholine which acts on the nicotinic acetylcholine receptor subunit α7 (α7nAChR) on innate immune cells to supress cytokine generation and release (327). Activation of β2 adrenoceptors is also immunosuppressant and this is thought to involve downregulation of the TNF-α signaling pathway within the DRG. This may contribute to the efficacy of serotonin - noradrenaline re uptake inhibitors (SNRI's) in neuropathic pain (332). This is because invading sympathetic fibres following nerve injury (44) provide a source of noradrenaline to active immunosuppressant β2 adrenoceptors and noradrenaline abundance is increased by the action of the SNRI, duloxetine. It has also been reported that activation of β2 adrenoceptors on microglia attenuates signs of neuropathic pain in a mouse model (333).

## Injury-induced structural changes in peripheral nerves

In addition to altered neuronal signalling, neuroinflammation, hyperexcitability, modulation of glial phenotypes and altered expression and function of numerous proteins, neuropathic pain is often associated with enduring structural changes in the peripheral, central and autonomic nervous systems (29, 44, 64, 334, 335).

### Reorganization of nociceptors

Neuropathic pain generated by peripheral nerve injury may involve sprouting of nociceptors into denervated territories such as skeletal muscle and skin. Here they replace the initial map and configuration of low threshold sensory axons that do not regenerate. Genetic ablation of nociceptors fully abrogates this type of re-innervation allodynia. These results reveal the emergence of a component of neuropathic pain that is driven by structural plasticity of peripheral sensory nerves, abnormal terminal connectivity and malfunction of nociceptors during reinnervation (29).

### Reorganization of peripheral sympathetic nerves

Peripheral nerve injury provokes sprouting of perivascular sympathetic axons and appearance of ectopic excitatory α-adrenoceptors on the cell bodies of primary afferent neurons and on their terminals at the site of injury (44, 255). This sprouting may be driven by the neurotrophic action of LIF or NGF (145, 336, 337) and/or may be a consequence of spontaneous afferent activity (338). This is yet another means by which nerve injury increases primary afferent excitability (44, 255, 339–341), leading to signs of neuropathic pain in animal models (342) and to the development of complex regional pain syndromes in humans (343).

## Failure to resolve chronic neuroinflammation

The chronic nature neuropathic pain (14, 18) contrasts with nociceptive pain and inflammation that are usually short-lasting or acute. This is because identified “off signals” actively supress the classical signs of inflammation that follow injury to non-neuronal tissue (344, 345). It is not yet understood why these signals fail to activate in neuropathic pain. “Off signals” include lipid-derived specialized pro-resolving mediators (SPMs) and anti-inflammatory cytokines such as IL-10 (346–348) and perhaps IL-6 (349, 350). Subtypes of immune cells such as antinociceptive (M2) macrophages, pain-resolving microglia and regulatory T-cells and modulators of the gut microbiota-immune system are also involved (11).

As emphasised above, spontaneous and ectopic activity in primary afferent fibres is crucial for the maintenance and persistence of signs of neuropathic pain (19, 30, 35, 197–205). Excessive neuronal activity as seen in neurogenic inflammation alters the phenotype of glia and immune cells to provoke the generation of inflammatory mediators (329). It is possible that incessant neurogenic neuroinflammation overcomes the resolution processes that normally terminate inflammation thereby contributing to the indefinite persistence of neuropathic pain.

In addition, the injury-induced structural changes in peripheral afferent (29) and sympathetic nerves (44) and in higher brain structures may be irreversible (64, 335). These enduring changes also contribute to the chronic nature of neuropathic pain (170).

## Spinal release of secondary mediators and their actions on spinal microglia and astrocytes

As mentioned already, nerve injury, via the action of primary mediators, upregulates mRNA for a variety of proteins and their receptors in primary afferent neurons (68, 351, 352). These include the secreted proteins CSF-1 (72, 110, 111, 353), CCL-2 (224, 354–356), TNF-α (357), IL-1β and IL-10 (354, 358), CXCL-12 (103, 104), CCL-21 (175, 352), Wnt5a (124) as well as neuropeptides such as CGRP (315) and NPY (351). These act as **secondary mediators** (68) that alert spinal microglia and astrocytes to the presence of peripheral nerve injury (Table 1 and Figure 1).

### Secondary mediators and sex-dependence of central sensitization

The best characterized **secondary mediators** include the cytokine CSF-1, the chemokines CCL-21 and CXCL-12 as well as Wnt5 and CGRP**.** Secondary mediators affect the properties of spinal microglia and astrocytes which in turn release **tertiary mediators** (68) (Table 1 and Figure 1). As will be described below, glial-derived tertiary mediators such as IL-1β and BDNF (117) act on neurons to bring about misprocessing of sensory information and increased activity and excitability leading to central sensitisation (359) (Table 1 and Figure 1).

Although microglia play a predominant role in central sensitization in males, invading macrophages and T-lymphocytes are predominant in females (360–362). Spinal signalling mechanisms invoked in males are therefore very different from those invoked in females (10, 352, 361). Lines of investigation initiated over 20 years ago have been directed towards understanding the numerous cellular and molecular processes that underlie this difference (68, 360, 362–369) and relevant and important differences will be outlined in the succeeding sections.

### Injury-induced signaling between primary afferent neurons and spinal microglia and/or astrocytes

#### Secondary mediator role of colony stimulating factor (CSF-1)

**Figure 3:**
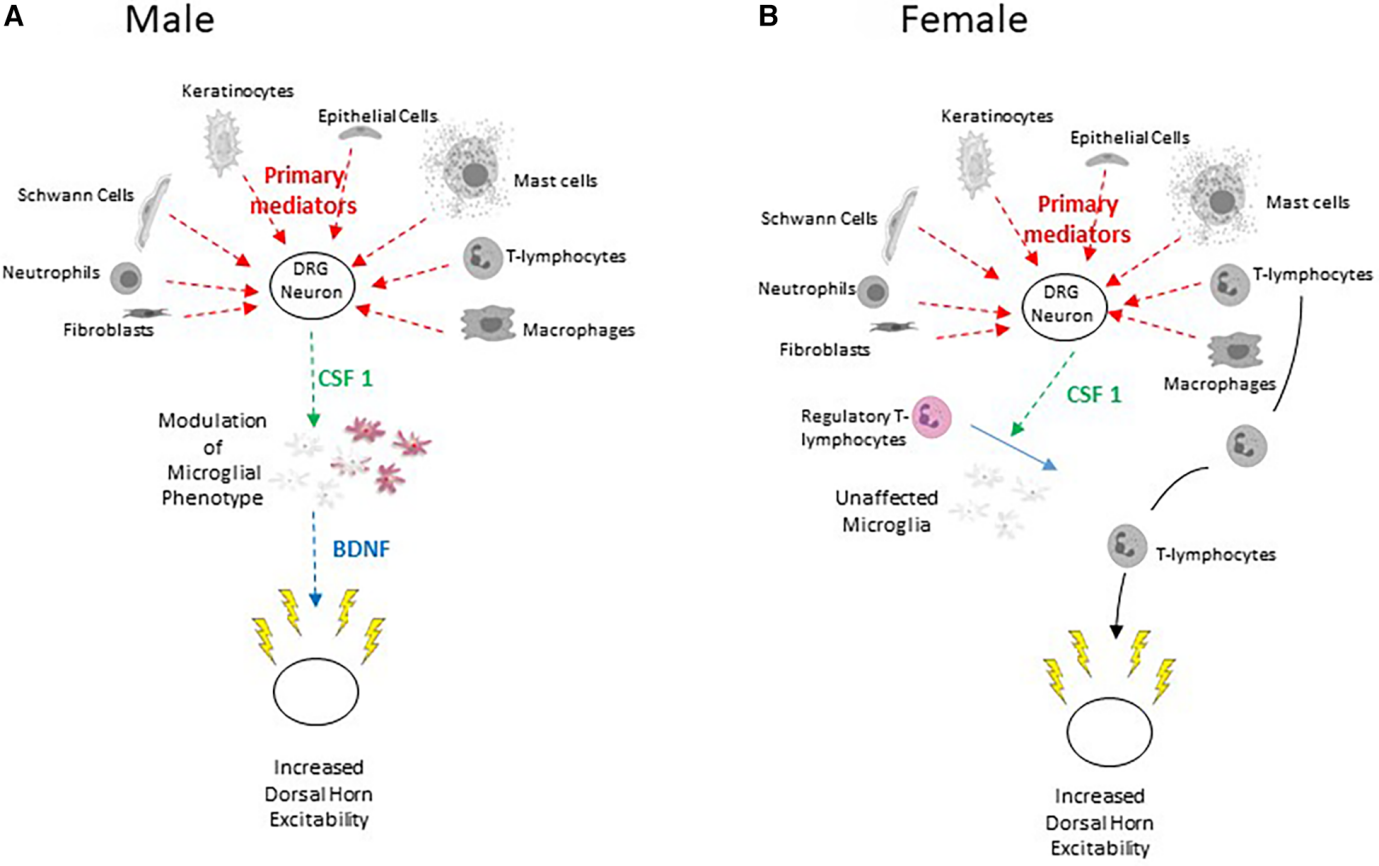
(**A**) Scheme to illustrate the sensory neuron – CSF-1 – microglia – BDNF pathway characterized in male rodents. Lightly coloured microglia represent those in the resting state and the purple colour represents their transformation to the P2X4 expressing phenotype (**B**) Scheme to illustrate train of events leading to injury-induced increased dorsal horn excitability in females.

Injury-induced release of inflammatory primary mediators such as interleukin 1β from macrophages and satellite glial cells in DRG promote *de novo* synthesis of CSF-1 in primary afferent neurons (68, 71, 72, 353, 370) (Table 1 and Figures 1, 3).

CSF-1 induces phenotypic modulation of spinal microglia and stmulates their proliferation and renewal. Intrathecal injection of this cytokine promotes mechanical allodynia in naïve male rodents but not in females (110, 112, 371, 372). Selective depletion of the *Csf1* gene from sensory neurons abrogates nerve injury-induced mechanical hypersensitivity and attenuates proliferation and phenotypic modulation of spinal microglial (71). Nerve-injury also increases mRNA for the CSF-1 receptor in microglia (112, 373) of male rodents. This activation persists for more than 6 weeks after injury (353).

As a corollary of this, it has been shown that alleviation of neuropathic pain by spinal cord stimulation involves a reduction in CSF-1 levels in DRG and spinal cord (373). Other work showed that following injury, the spinal invasion of regulatory T-lymphocytes (suppressor T-cells) attenuate modulation of microglial phenotype in females only. This is supported by the observation that female mice engineered to lack regulatory T-lymphocytes show increased injury induced CSF1-induced microglial modulation and pain hypersensitivity similar to that seen in males (374) (Figure 3B).

In male mice, a major consequence of the release of CSF-1 from primary afferent terminals is promotion of the expression of the ionotropic ATP receptor, P2X4 in spinal microglia (110, 112, 113). ATP-derived from dorsal horn neurons activates these receptors, promoting Ca^2+^ influx and release of the tertiary mediator BDNF (Figure 3A) (22, 118–120, 375–379). This mechanism is crucial to microglial signalling and the development of central sensitization in males (376, 380) but not in females (362, 381).

Taken together with the observation that exposure of dorsal horn neurons to CSF-1 increases their excitability via a BDNF-dependent process (113), these data strongly support the role of CSF-1 as a secondary mediator signalling between injured primary afferents and microglia (68) (Figure 3A).

#### Secondary mediator role for CXCL-12

In addition to CSF-1, several lines of evidence support the role of CXCL-12 (C-X-C motif chemokine 12) in signalling between injured sensory neurons and astrocytes (102, 104). CXCL-12 and its cognate receptor, CXCR-4 are constitutively expressed in spinal astrocytes and microglia of male rodents (102, 382).

Peripheral nerve injury upregulates CXCL-12 in DRG and CXCR-4 in spinal cord astrocytes (103, 104, 382–384) as a possible consequence of miR-130a-5p downregulation (385) and/or the action of TNF-α (103). As already mentioned, intrathecal administration of CXCL-12 induces hypersensitivity in naive male mice (382). In addition, CXCL-12 antagonists transiently reverse allodynia after DRG crush in male mice (104).

CXCL-12 is thus involved in signaling from injured primary afferents to astrocytes (385). In addition, by virtue of the presence CXCR-4 on microglia, it is also involved in signalling between astrocytes and microglia (382). The CXCL-12/CXCR4 system may also be involved in hyperalgesic priming (386). Hyperalgesic priming describes enhancement of responses to potentially painful stimuli following repetitive stimulation (369, 387, 388). CXCL-12 thus functions as both a secondary mediator between primary afferents and spinal glial cells as a primary mediator between activated immune cells and primary afferents (see Figure 1, Table 1 and above).

#### Secondary mediator role for CCL-21

Intrathecal administration of CCL-21 (chemokine C-C motif ligand 21) produces pain-like behaviour in naive male mice and CCL-21 neutralizing antibodies or blockade of its cognate CXCR-3 receptor attenuates nerve injury-induced pain (114). The failure of CCL-21 deficient male mice to display tactile allodynia following nerve injury (389) is attributed to failure of microglia to upregulate the purinergic P2X4 receptor (115, 175). CCL-21 is upregulated in DRG following nerve injury, vesicles containing CCL-21 are preferentially transported into axons (390), and it can be released from terminals of injured neurons (116, 391). These findings identify CCL-21 as a third, pro-inflammatory secondary mediator between injured primary afferents and microglia in male mice (68, 175, 383).

CCL-21 also signals to astrocytes where it triggers intracellular Ca^2+^ transients (385, 392). Despite these findings which were made in male rodents, RNA profiling of the DRGs of humans with neuropathic pain suggests, that CCL-21 may only be involved in female patients (352). These findings underline the importance of both sex and species dependencies of pain etiology.

#### Secondary mediator role for CGRP

Stimulation of primary afferents with capsaicin promotes CGRP release in the spinal dorsal horn and this release is increased following nerve injury (315). Since CGRP also affects microglia function (330) it, like other secondary messengers, alerts microglia to the presence of peripheral injury.

In the spared nerve injury (SNI) model, there is a transient effect of CGRP antagonists on mechanical hypersensitivity in female mice only. Consistent with these findings, intrathecally administered CGRP causes a long-lasting, mechanical hypersensitivity in female mice but more transient effects in males. In addition, hyperalgesic priming in female, but not in male rodents is blocked and reversed by intrathecal injection of CGRP antagonists. Systemic administration of a CGRP antibody, blocks hyperalgesic priming specifically in female rodents yet fails to reverse it once it is established. As will be mentioned below, part of the action of CGRP may involve direct modulation of spinal neurons without the intervention of microglia or astrocytes (323).

### What is the role of IL-6 in spinal hyperactivity?

Unilateral CCI (chronic constriction injury) increases IL-6 mRNA and protein bilaterally in both neurons and satellite glial cells of the DRG (83). IL-6 promotes hyperalgesic priming in rodents (323) and conditional knockout of its cognate gp130 receptor in nociceptors abrogates pain in inflammatory and tumor-induced pain models (393). Although these results are consistent with a secondary mediator role for IL-6, other work suggests that it may have an anti-nociceptive action both in the periphery and at the spinal level following SNI (spared nerve injury) in rodents (349, 350) and may be capable of inducing a desensitized microglial phenotype (394).

### What is the role of interferon gamma in spinal hyperexcitability?

IFN-γ alters spinal microglial function and induces tactile allodynia. Genetic ablation of the interferon receptor (IFN-γR) impairs nerve injury-evoked allodynia and prevents phenotypic modulation of spinal microglia (395). The P2X4 receptor is upregulated in IFN-γ stimulated - microglia and, as mentioned already, these purinergic receptors play a crucial role in the onset of neuropathic pain in males (118, 120, 375, 377, 378). IFN-γ also increases dorsal horn excitability (396, 397) and facilitates synaptic transmission between C-fibres and Lamina 1 neurons via a microglial-dependent mechanism (132). Although IFN-γ is found in DRG neurons (398) and the level of IFN-γ is increased in spinal cord following peripheral nerve injury (399) this may originate from invading T-lymphocytes. However, given the role of T-lymphocytes in females (361, 362), IFN-γ may be important in pain aetiology in women.

### What is the role of cathepsin and fractalkine (FKN; CX3CL-1) in spinal hyperexcitability?

The lysosomal cysteine protease, cathepsin S is released from microglia by a P2X7-dependent mechanism (400). Cathepsin S, as well as the metalloproteinase ADAM_10_ and TNF-α converting enzyme liberate the soluble form of the chemokine, fractalkine (FKN; CX3CL-1) from dorsal horn neurons (125, 126, 401) (Figure 2B).

The transmembrane form of FKN and its cognate receptor (CX3CR-1) are expressed constitutively in spinal cord neurons (402, 403). CX3CR-1 which is strongly expressed in dorsal horn microglia (125, 403, 404), is upregulated after nerve injury. In naïve animals, intrathecal injection of FKN produces mechanical allodynia and thermal hyperalgesia whereas injection of an antibody raised against CX3CR-1 attenuates signs of neuropathic pain in animal models (404). This is consistent with the observation that peripheral nerve injury fails to provoke allodynia in mice lacking CX3CR-1 (405).

Spinal nerve ligation (SNL) also increases the level of the soluble form of FKN in cerebrospinal fluid (401) and such release appears to be obligatory for the expression of neuropathic pain (127, 383, 406). Soluble FKN modulates microglial phenotype leading to the generation of tertiary mediators such as TNF and IL-1β (404, 407).

Antibodies raised against CX3CR-1 reduce nociceptive responses when administered as long as 5–7 days after CCI suggesting that the prolonged release of FKN contributes to the maintenance as opposed to the onset of neuropathic pain. This may also relate to the observation that SNL provokes *de novo* expression of FKN in dorsal horn astrocytes (403).

### Modulatory role of glutamate

In addition to producing synaptic potentials in almost all CNS neurons, glutamate affects astrocytes, T-cells, endothelial cells, microglia and vascular cells by interaction with mGluRs (329, 408, 409). These actions are predominantly anti-inflammatory (410). For example, mGluR5 activation in spinal microglia inhibits the release of inflammatory mediators both *in vitro* (410) and *in vivo* (411). Also, activation of group I mGluRs in astrocytes leads to increased glutamate and potassium uptake (412). These actions may thus be associated with offset of neuroinflammation rather than its onset.

## Release of tertiary mediators from astrocytes and microglia

### Release of BDNF in the spinal dorsal horn

The secondary mediator CSF-1 interacts with CSF-1R on spinal microglia (353). This leads to increased expression of the **tertiary mediator** BDNF as a result of up regulation of the *Bndf* gene (413). As illustrated in Figure 3, the release of BDNF plays an indispensable role in the onset and maintenance of neuropathic pain in male but not in female rodents (22, 68, 113, 117, 119, 121–123, 414–420). BDNF acts primarily via TrkB to increase dorsal horn excitability (113).

Exposure of dorsal horn neurons to CSF-1 also increases the frequency and amplitude of sEPSC's (spontaneous excitatory postsynaptic currents) and this effect is abrogated by the BDNF binding protein TrkB-fc (113). These findings underline the importance of the sensory neuron- CSF-1 -microglia - BDNF signalling process in the aetiology of neuropathic pain (14, 22, 68, 110, 183, 421) (Figure 3A).

### Role of ATP in BDNF release from microglia

ATP activation of microglial P2X4 receptors leading to the release of BDNF is involved in the aetiology of neuropathic pain in males, but not in females. This is congruent with the absence of functional P2X receptors on microglia of female rodents (364). There is also evidence for a role of microglial metabotropic P2Y6, 11, 12, 13 and 14 receptors in the onset of neuropathic pain (68, 422–427). Primary afferent neurons are not the primary source of ATP following peripheral nerve injury. It may rather derive from neurons in the superficial dorsal horn itself (428) as well as from microglia themselves (429). BDNF release from neurons is vesicular and dependent on extracellular Ca^2+^ (118, 119, 375, 377, 378).

### Wnt signalling and release of BDNF from microglia

The action of ATP on microglia is not the sole mechanism for promoting BDNF release. Wnt proteins that are upregulated in the spinal cord in various pain models (50, 124, 429–431) activate “frizzled” receptors (432) on microglia to increase expression of BDNF and promote its release (420, 433). This phenomenon has been examined in models of HIV pain which involve exposure of sensory neurons to toxic viral coat proteins such as Vpr1 (49) or gp120 (433, 434). The latter promotes allodynia and increases glutamatergic neuronal activity leading to NMDA receptor activation and increases the level of intraneuronal Ca^2+^. This, in turn promotes Wnt protein synthesis and release (435, 436).

### Time course of BDNF release in the dorsal horn

Phenotypic modulation of microglial function in rodent dorsal horn persists for more than 3 months after injury (437). Thus sequestration of BDNF with TrkBFc (438) or selective depletion of spinal microglia with the targeted immunotoxin Mac1-saporin almost completely reverses mechanical and thermal allodynia up to 3 months after injury. By contrast, intrathecal injection of a cocktail of antibodies against IL-1β, TNF-α, and IL-6 significantly attenuates tactile and cold allodynia at 2 weeks but not at 3 months after injury. These findings suggest that different mediators should be targeted in the short vs. long term management of neuropathic pain (437).

### Release of IL-β in the spinal dorsal horn

The tertiary mediator, IL-1β is produced and released from macrophages, astrocytes and microglia (18, 439, 440). Release of IL-1β from microglia is a consequence of activation of P2X7 receptors (164, 380, 441, 442) and may be provoked by the action of FKN (407). In agreement with this, it has been reported that the Ca_v_1 channel blocker, cilnidipine which also blocks microglial P2X7 receptors, impairs IL-1β release and reverses SNL-induced mechanical hypersensitivity (142). It has also been suggested that P2X4 receptors interact intracellularly with P2X7 receptors to augment P2X7 receptor-mediated IL-1β release (442).

### Role of exosomes

In addition to the extracellular actions of BDNF and IL-1β, cell-to-cell transport of material via exosomes or extracellular vesicles is now believed to contribute to the development of central sensitisation (183, 443–449). Extracellular vesicles are released from both microglia (450) and astrocytes (451) and are taken up by neurons (447). They may serve as a conduit for the transfer of microRNA's between cell types (452). For example, Na_v_1.7 protein may be transported from primary afferents to the dendrites of lamina II neurons; a process which may be effected by transfer of exosomes (449).

## Actions of BDNF in the dorsal horn

The cellular mechanisms that are involved in actions of microglial-derived BDNF include enhancement of excitatory processes and attenuation of inhibition (22, 170). In addition to actions on neurons, BDNF also activates astrocytes (453) which release additional mediators such as FKN (403) and IL-1β (18).

### Increased excitatory drive to excitatory neurons and decreased drive to inhibitory neurons

Exposure of rat *substantia gelatinosa* neurons to BDNF increases excitatory synaptic drive to excitatory neurons and decreases excitatory drive to inhibitory neurons (122, 414). In mice, effects of BDNF are dominated by increased excitatory drive to excitatory neurons (113). Whilst resting potential, rheobase, input resistance and excitability are little affected in rat neurons (113, 122, 414), the altered synaptic activity increases spontaneous AP discharge in excitatory neurons whilst reducing it in inhibitory neurons (414).

Several observations show that these actions of BDNF are relevant to injury-CSF-1-microglia-BDNF evoked central sensitization (Figure 3A). Firstly BDNF-induced changes in synaptic transmission and its lack of effect on the intrinsic excitability the cell bodies of lamina II neurons very much parallel those invoked by peripheral nerve injury (122, 454–456). Secondly, Ca^2+^ responses evoked by neuronal depolarization are enhanced by BDNF and by conditioned medium from lipopolysaccharide-activated microglia. The effect of this conditioned medium is attenuated by sequestering BDNF with TrkBd5 (122). Thirdly, the secondary mediator CSF-1 increases synaptic excitation of excitatory lamina II neurons in mice and this effect is abrogated by sequestering BDNF with TrkBfc (113).

It should be noted however that mitochondrial dysfunction following peripheral nerve injury and the resultant high levels of superoxide may also contribute to increased excitatory synaptic strength in dorsal horn neurons and neuropathic mechanical hypersensitivity (457).

### BDNF disinhibition by perturbation of chloride gradients

**Figure 4:**
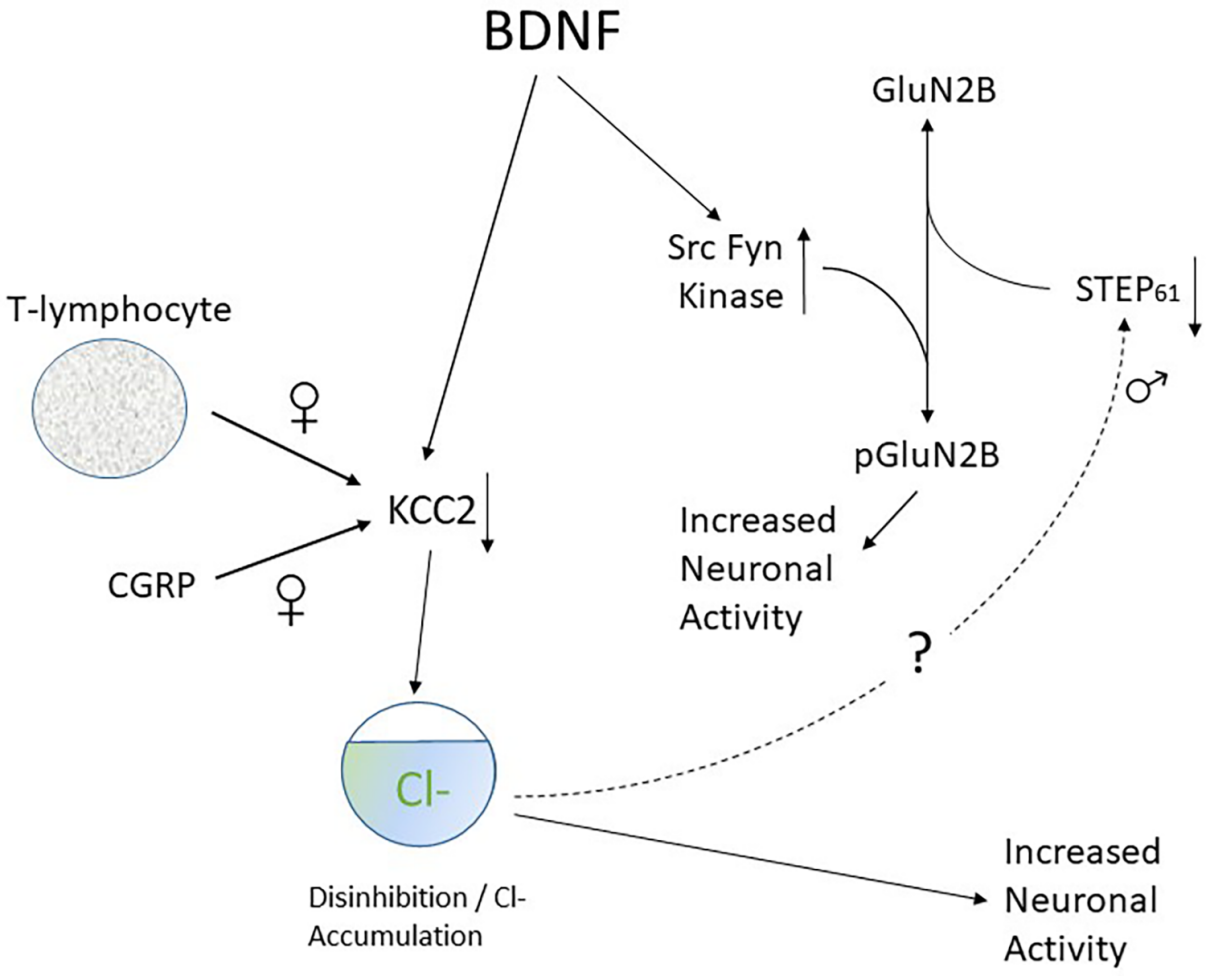
Scheme to illustrate connection between KCC2 downregulation and GluN2B phosphorylation and its sex dependence.

CCI of the sciatic nerve reduces expression of the potassium-chloride exporter (KCC2) in lamina 1 neurons of the dorsal horn (458, 459). The resulting intracellular accumulation of Cl^−^ reverses the Cl^−^ concentration gradient such that normally outward, inhibitory GABAergic synaptic currents mediated by Cl^−^ influx become inward excitatory currents mediated by Cl^−^ efflux (458–460). Knockdown of KCC2 in uninjured rats reduces pain thresholds and induces neuropathic pain-like behaviors. By contrast, rescue of KCC2 expression abrogates signs of neuropathic pain in nerve injured animals (461, 462). Taken together and as illustrated in Figure 4, these findings strongly implicate perturbation of the Cl^−^ gradient and the phenomenon of disinhibition in the pathophysiology of central sensitization (121, 458).

BDNF is responsible for downregulation of KCC2 protein levels in male rats (121, 419). Thus, administration of ATP-activated microglia reproduces the shift in anion gradient seen after nerve injury in the same way as BDNF. Also, blocking TrkB or using interfering RNA against BDNF reverses both injury-induced pain behaviors and the shift in Cl^−^ gradient (121). Changes in KCC2 expression in deep dorsal horn neurons are confined to nociceptive neurons that project via the spinothalamic tract whereas wide dynamic range (WDR) neurons that are activated by a variety of sensory modalities are unaffected (461). It has also been shown that neurons in lamina I are more susceptible to changes in Cl^−^ gradient than those in lamina II (459) and biophysical and modelling analysis shows this loss is especially effective in promoting increased neuronal firing (463). These are important observations as lamina I and deep dorsal horn nociceptive neurons are the primary site for relay of nociceptive information to the brain (464–466). Loss of GABAergic inhibition enables non-noxious Aβ fiber-mediated excitatory transmission to access and excite the pain transmitting neurons of the superficial spinal dorsal horn. Thus, as already mentioned, tactile activation of Aβ fibres is perceived as pain and this process plays a role in the establishment of mechanical allodynia (467–469).

Descending serotonergic inhibition of nociceptive processing from the *nucleus raphe magnus* becomes excitatory and proalgesic in rats subject to spared nerve injury (SNI). This change is also dependent on collapse of the Cl^−^ gradient following KCC2 hypofunction in the dorsal horn as the KCC2 enhancer CLP290 restores both 5-HT–mediated descending inhibition and analgesia (470).

KCC2 downregulation also contributes to pain hypersensitivity in females (363). Whereas this is mediated by release of BDNF from microglia in males, it involves activation and invasion of adaptive immune cells such as T-lymphocytes in females (362, 381) (Figure 3) as well as downregulation of KCC2 expression by CGRP (323).

### BDNF and function of spinal NMDA receptors

BDNF enhances excitatory responses to NMDA in rat spinal cord *in vitro* (471). In male rodents, this potentiation is dependent on BDNF-mediated GABA disinhibition. By processes yet to be discovered, KCC2-dependent disinhibition promotes downregulation of the tyrosine phosphatase STEP_61_. Loss of function of STEP_61_ phosphatase then clears the way for phosphorylation of GluN2B subunits by the Src family kinase Fyn (472). As illustrated in Figure 4, synaptic NMDAR responses are therefore enhanced and neuronal excitability is increased. Decreased activity of STEP_61_ is both necessary and sufficient to affect GluN2B function (473), This sequence of events is supported by the observation that blocking of KCC2-mediated disinhibition with acetazolamide (474) reverses the downregulation of STEP_61_ and attenuates behavioural hypersensitivity generated by chronic inflammation.

In female rats however BDNF fails to downregulate KCC2 and STEP_61_ and to upregulate pFyn, GluN2B and its phosphorylated form GluN2B. This means that BDNF fails to affect synaptic NMDAR responses in lamina I neurons of females. Ovariectomy recapitulates the male pathological pain neuronal phenotype in female rats, with BDNF driving coupling between disinhibition and NMDAR potentiation in lamina I neurons following the elimination of sex hormones (475).

This sex difference in spinal pain processing in rodents is conserved in humans. Thus *ex vivo* spinal treatment with BDNF downregulates KCC2 and STEP_61_ and upregulates markers of facilitated excitation in superficial dorsal horn neurons from male but not female human organ donors (475).

In addition to the postsynaptic effects described above (121, 473, 475), BDNF activation of TrkB increases the function of presynaptic NMDA receptors on primary afferent terminals (476). This leads to the potentiation of glutamate release from primary afferents that is observed after SNL (477) and may account for the increased frequency of sEPSC's seen in some dorsal horn neurons in the presence of BDNF(414). Functional upregulation of GluN2B subunits of NMDA receptors (478) may also account for the observation that long term potentiation (LTP) of synaptic transmission of C-fibre responses is enhanced by BDNF (479).

## Actions of other tertiary mediators in the dorsal horn

### Effects of interleukin 1β in the dorsal horn

The level of IL-1β is elevated in the cerebrospinal fluid (CSF) of patients with complex regional pain syndrome (480) and in spinal cords obtained post-mortem from patients with painful HIV related neuropathy (50). As already mentioned, activation of P2X7 receptors promotes release of IL-1β from microglia (142, 164, 380, 441) and this is amplified by the action of FKN (407).

Microglial derived IL-1β stimulates astrocytic production of TNF-α well as IL-1β itself (440, 481) thereby amplifying the overall IL-1β signal. IL-1β promotes internalization of the astrocytic glutamate transporter (EAAT2) thereby reducing the capacity of astrocytes to take up glutamate (482, 483). Loss of EAAT2 function thus augments excitatory synaptic transmission and induces hyperalgesia and increased sensitivity of dorsal horn neurons to primary afferent stimulation (484, 485). Activated astrocytes also release CSF-1 (187) thereby amplifying signaling via the CSF-1-microglia-BDNF cascade (Figure 3A). Astrocytes also release the NMDA receptor co-agonist D-serine (486) thereby further augmenting overall dorsal horn excitability.

### Effects of IL-1β on synaptic transmission in the spinal dorsal horn

In a similar fashion to BDNF, IL-1β increases glutamate release from primary afferents and augments excitatory synaptic transmission between primary afferent C-fibres and lamina 1 neurons. It also amplifies Ca^2+^ responses evoked by exposure of neurons to 20 mM K^+^ (143, 407, 483).

Like BDNF, IL-1β also does not affect the membrane potential or rheobase of lamina II neurons, suggesting that most of its effect on dorsal horn excitability can be ascribed to changes in synaptic transmission (143, 144). Exposure of rat spinal cord to IL-1β for 6–8 d increases the amplitude of spontaneous EPSC's (sEPSC) in putative excitatory ‘delay’ neurons, and decreases the frequency of spontaneous IPSC's (sIPSC). These actions are similar but not identical to those seen with BDNF or peripheral nerve injury (414, 454, 455). Acute application of IL-1β increases the amplitude of AMPA and NMDA currents dorsal horn neurons (487). Its effect on glutamate release can be ascribed to augmentation of presynaptic NMDA receptor function (483) where signaling between IL-1r and NMDA involves the sphingomyelinase/ceramide pathway (477, 483).

Taken together, all of these actions of IL-1β would be expected to increase dorsal horn excitability and to facilitate the transfer of nociceptive information.

### Effects of tumor necrosis factor-α in the dorsal horn

TNF-α decreases the excitability of a subset of spinal GABAergic neurons by suppression of current through HCN channels (488). These effects diminish with time suggesting TNF-α may be primarily involved with the induction rather than the persistence of neuropathic pain (489). As might be expected, blockade of TNF receptor 1 attenuates signs of neuropathic pain in the CCI model but this only occurs in males and not in female rodents (150).

Although FKN action on microglia and potentiation of synaptic transmission in the dorsal horn involves IL-1β but not TNF-α (407), it does appear to be inolved in the generation of a phenomenon named “gliomic LTP” (151, 490). By contrast with classical LTP which is highly localized, “gliomic LTP” spreads extensivly throughout the dorsal horn by the action of TNF-α and of the NMDA co-agonist D-serine (490).

### Effects of interleukin-17 in the dorsal horn

IL-17 is expressed in spinal astrocytes and its cognate receptor is expressed in neurons, especially by those expressing somatostatin (133). SNI-induced static and dynamic allodynia are prevented by intrathecal injection of IL-17 neutralizing antibody and attenuated in IL-17a mutant mice. IL-17 neutralizing antibodies supress LTP of C-fiber evoked field potentials in spinal cord and intrathecal injection of IL-17 or its overexpression in astrocytes produces mechanical allodynia and facilitates spinal LTP (134). IL-17 also supresses inhibitory transmission and enhances excitatory transmission in spinal lamina II_o_ (133). It may thus serve both as a primary and tertiary mediator (Table 1) but the mechanism of its release from astrocytes is yet to be determined.

## Role of supra-spinal structures in pain etiology

Inasmuch as injury-increased peripheral hyperexcitability leads to enduring changes in the dorsal horn, increased dorsal excitability contributes to alterations in supraspinal structures.

### Changes in sensory pathways in supra-spinal brain regions

Several detailed reviews address supra-spinal changes associated with neuropathic pain (491, 492).

Blood borne inflammatory mediators (38, 493) generated at the site of injury open tight junctions between capillary endothelial cells leading to increased permeability of the blood-brain barrier (192). This allows supra-spinal neurons to interact with blood cells and respond to the cytokines and chemokines they produce (195). Following peripheral injury, afferent information is modulated in various thalamic nuclei (494), somatosensory cortex (495), insular and anterior cingulate cortex (491, 496), nucleus accumbens, and amygdala (497–500). Ascending pathways also interact with the mesolimbic dopamine system (501),

Peripheral nerve injury changes the properties of microglia in the contralateral thalamus, sensory cortex and amygdala as might be expected from the known anatomical arrangement of ascending sensory fibres. Brain regions not directly involved in either sensory or affective aspects of pain such as the motor cortex, do not display altered microglial function (497). This selective modulation of microglia and immune cells in nociceptive pathways (497) may be a consequence of localized neurogenic neuroinflammation as a result of enduring intense activity (329).

Cortico-cortical or cortico-subcortical interactions contribute to the co-morbidies seen in some patients. For example, one form of long-term potentiation (LTP) in the anterior cingulate cortex (ACC) which is triggered by the activation of NMDA receptors and expressed by an increase in AMPA-receptor function, sustains the affective component of the pain state. Another form of LTP in the ACC, which is triggered by the activation of kainate receptors and expressed by an increase in glutamate release, may contribute to pain-related anxiety (491).

There are several parallels between injury-induced cellular changes in higher centres and those seen in the periphery or spinal cord. For example, peripheral neuropathy induces HCN channel dysfunction in medial prefrontal cortex (502) and thalamus (503, 504) and Na_v_1.3 function is altered in thalamic neurons (505, 506). Both channel types are similarly affected by peripheral nerve injury (208). These findings are fortuitous in terms of drug action and identification of therapeutic targets; drugs developed to act peripherally may also exert beneficial effects as a result of similar central actions.

### Descending control of nociception

Cortical structures modulate nociception through descending control of spinal circuitry (507). This occurs by direct corticospinal projections as well as activation of structures in the brainstem such as the periaqueductal grey matter, *locus coeruleus, raphe nuclei* and rostroventral medulla (492). Descending inhibition of spinal nociceptive processing is mediated via 5HT_7_ receptors and α_2_ adrenoceptors whereas serotonergic activation of metabotropic 5HT_2_ receptors and ionotropic 5HT_3_ receptors facilitates transmission (508–512). This explains the effectiveness of noradrenaline-serotonin reuptake inhibitors (SNRI) in pain management (16) and the limited efficacy of selective serotonin reuptake inhibitors.

There is normally a balance between descending inhibition and excitation but after peripheral nerve injury the excitatory processes gain the upper hand (470, 513). These changes have been associated with the persistence as opposed to the onset of pain (514, 515).

### Role of mesolimbic reward circuitry in pain etiology

Peripheral nerve injury impairs dopamine release in the reward circuitry associated with the mesolimbic system (497, 501). This may also relate to the changes in affect (anxiety, depression) experienced by neuropathic pain patients (516). Peripheral nerve injury selectively increases excitability of the *nucleus accumbens* indirect pathway spiny projection neurons and alters their synaptic connectivity. In addition, tactile allodynia can be reversed by inhibiting and exacerbated by exciting these neurons. This suggests that neurons in the *nucleus accumbens* not only participate in the central representation of pain, but that they may gate activity in ascending pathways associated with expression of pain in higher centres (517).

## Why are there no new drugs? What can we do about it?

Management of neuropathic pain in the clinic involves serotonin-noradrenaline reuptake inhibitors (SNRI), gabapentinoids, capsaicin patches, classical tricyclic antidepressants such as amityptyline, high dose opioids as well as tramadol and botulinum toxin (12, 14, 16, 22). Although the effectiveness of these drugs is limited, extensive preclinical research as outlined above has failed to reveal any effective therapies since the approval of tramadol, a mild opioid with SNRI properties, in the mid 1990′s. To put this into perspective, hundreds of drug targets have been identified over the years; a perfunctory examination of publications appearing in the first 4 months of 2023, identified about 650 papers that dealt with neuropathic pain. Of these, 28 studies identified a “magic molecule”, that was implicated pain etiology in an animal model. Despite this proliferation of potential drug targets, no new drugs have appeared.

What can be done? How can the data gap between animal studies and clinical practice be bridged?

## Improved basic science approaches

### Addressing different mechanisms evoked by different types of injury

Classical rodent pain models such as SNI (spared nerve injury), CCI (chronic constriction injury), SNL (spinal nerve ligation) or CCDRG (chronic constriction of DRG) have revealed general principles that help to explain the aetilogy of neuropathic pain. These include the identification of various chemokines, cytokines, neuropeptides and growth factors as primary, secondary or tertiary mediators, the concept of neuroinflammation and bidirectional signalling between neurons and immune cells, alterations in synaptic transmission, ion channels and descending modulation, the roles of microglia and astrocytes, central sensitization and role of peripheral spontaneous activity (12, 15, 20, 22, 27, 47, 68, 183, 208, 210). These findings fall short of addressing the multiplicity of chronic pain presentations in the clinic (8, 47) as even in animal models, different types of nerve injury provoke distinct behavioral, physiological and cellular responses.

For example, mechanical allodynia produced by CCI is short-lived and recovery is seen in about 4 weeks whereas that produced by SNI persists for 7 weeks or more (61, 72). Similarly, changes in synaptic transmission in lamina II neurons are more robust after sciatic CCI than after complete sciatic nerve section (axotomy) (455). These findings relate to the observation that CCI promotes stronger and more long lasting upregulation of the inflammatory mediators IL-1β, TNF-α, IL-10, MCP-1/CCL-2 in nerve stumps than nerve crush (354), Recent work has also shown that glycine inputs onto radial neurons in spinal lamina II are reduced following partial nerve ligation (PNL) of the sciatic nerve, this finding was not seen in animals subject to CCI (63).

Whilst neuropathic pain associated with multiple sclerosis is characterized by loss of spinal neurons (371), this is not seen with CCI (518, 519). Although the NGF binding antibody tanezumab is effective in some pain patients (238), studies in animal models suggest that NGF itself may be effective in management of pain and neuropathy associated with HIV infection (520).

The nature of peripheral injury also dictates the precise spinal circuitry involved in the generation of mechanical allodynia (521). Thus nerve injuries generate allodynia by activation of excitatory neurons that express protein kinase C gamma (PKCγ) (522) whereas mechanical allodynia induced by inflammation involves excitatory neurons that are calretinin positive (523). Cholecystokinin (CCK) positive neurons are important in both situations. Punctate allodynia as produced by Von Frey filaments is distinct from dynamic allodynia that is produced by brushing a cotton swab across the hindpaw skin (521). A subset of CCK positive neurons are primarily involved in conveying dynamic rather than punctate allodynia.

Work using knockout mice has shown that deficiency of CCL19/21 attenuates nerve injury evoked pain but not the hyperalgesia observed in an animal model of multiple sclerosis (116).

This issue of injury-specific mechanisms is starting to be resolved as basic scientists have increasingly turned their attention to disease models rather than classical neuropathic pain models such as CCI and SNI. There are now reliable animal models for diabetic neuropathy (524), multiple sclerosis (34), phantom limb pain (30), chemotherapy induced pain (53, 129, 525), spinal cord injury (31) and trigeminal neuralgia (526).

In the situation of inflammatory as opposed to neuropathic pain, it has recently been reported that nociceptor-neuroimmune interactomes reveal cell type- and injury-specific pathways in three different inflammatory models (527). The availability of a similar database in the neuropathic pain field would be of great advantage to developing specific treatments.

### Objective and non-invasive assessment of pain in animal models

Another major step forward from the basic science perspective is the ongoing improvement in pain assessment in animal models. Regardless of the type of nerve injury used, preclinical effectiveness of therapeutic interventions has classically been assessed in rodent models by examining drugs’ ability to attenuate withdrawal responses to stimuli that would normally be innocuous (47, 60, 61). This typically involves measurements of mechanical or thermal withdrawal thresholds to quantify hyperalgesia or allodynia. Such responses are difficult to quantify as they may be influenced by the subjective impressions of the investigator as well as the olfactory signals they emit. For example, male investigators promote analgesia in female mice (528). In addition, withdrawal responses to innocuous stimuli in injured animal may simply reflect activation of spinal reflexes (529, 530) rather than *bona fide* manifestations of pain. This may help to explain why classical rodent models have limited ability to predict clinical efficacy (17, 47, 529, 531). In view of this, non-invasive models for objective assessment of chronic pain have been developed. These involve assessment of hypersonic vocalisation, facial grimace score, quantification of social interaction, rearing and nest-building (47, 532–537) and the use of operant models in which the animal is required to make a decision based on the cortical processing of a noxious stimulus (538–540).

The use of operant and non-invasive protocols to effect translation between preclinical observations and development of effective therapeutic approaches may be further refined by combining findings from as many as 6 operant protocols (534).

### Use of human nerves in the laboratory

Advances in technology now permit the use of human nerves in the laboratory (475). Because this has identified the cellular basis for differences in nociceptive processing between humans and rodents (541), the use of such models may be a way forward for identification of more relevant therapeutic targets. Human nociceptors are more heterogeneous than those in rodents and there are also differences in ion channel function and expression leading to differences in cellular excitability (19, 542, 543). Most human DRG neurons exhibit TRPV1 receptor channels but these are expressed exclusively in peptidergic nociceptors in rodents (544). A subpopulation of human DRG neurons display a relatively large constitutive Ca^2+^ channel current and although HVA Ca^2+^ current density is significantly smaller in human than in rodent DRG, the proportion of nifedipine-sensitive (Ca_v_1.2) currents is much greater (543). Although this identifies dihydropyridines as a potential therapeutic approach to some types of neuropathic pain, their further development is limited by their propensity to produce postural hypotension (545).

Contemporary methodologies that allow the collection of data from human nerves include observation of nociceptor morphology in skin biopsy samples (546) and use of explant cultures of DRG neurons from aborted fetuses (49). Human DRG's have also been acutely isolated from organ donors or cadavers or from patients undergoing surgical treatment for spinal reconstruction (475, 543, 547).

The use of human induced pluripotent stem cells (hiPSC) differentiated into nociceptive sensory neurons may provide a means to address the limited availability of human DRG neurons (548–554). The use of hiPSC has the advantage of providing large numbers of human neurons, glia and immunocompetent cells (555). This in turn allows the application of high throughput technologies to screen small molecule therapeutic agents to modify nociceptor function (556).

### Recognition of differences in pain processing in female versus male rodents

In recent years, considerable attention has been paid to analysis of molecular mechanisms of pain in male vs. female rodents (10, 362, 381, 475). As already emphasized, microglia are not required for mechanical sensitivity to pain in female mice as they require activation of adaptive immune cells such as T-lymphocytes (362, 381). The difference may result from a lack of P2X4 receptors in the microglia of females (364, 376). Despite this, behavioral responses to nerve injury in female rats are similar to those seen in males and both involve downregulation of KCC2 and perturbation of Cl- gradients (363). Because BDNF is not necessary for the development of allodynia in females (362), the mediator released from adaptive immune cells remains to be determined. The possible involvement of IFN-γ has already been alluded to.

Numerous differences in pain mechanisms in males vs. females have emerged over recent years (10). For example loss of GABA_A_ receptors containing the α6 subunit plays a predominant role in female rodents (557). The relative importance of CGRP in females (323), the role of macrophage derived IL-23 (368), and the absence of functional P2X receptors on microglia of female rodents have already been alluded to (364).

This realization has obvious implications for the design of clinical trials (10); potential new therapies must be evaluated in women and men as separate subgroups of patients.

### Clinical approaches

#### Recognition and appreciation of different pain aetiologies in the clinic

As mentioned in the introduction, patients with neuropathic pain are heterogeneous in clinical presentation, pathophysiology, aetilogy, causative injury, genetics and prior life experience (5). This is reflected in a large variability in their response to treatment (8, 25).

One way forward from the clinical perspective is the quantitative, phenotypical stratification of patent types in order to delineate responders from non-responders. This statistical subgrouping of patients can have a role in determining treatment (12, 25, 558, 559). Several tools are available for patient stratification.

Firstly, quantitative sensory testing (QST) enables identification of various subtypes of neuropathic pain by formalization and quantification of an existing battery of neurological tests, such as response to von Frey filaments, vibration, heat, pressure and cold as well as wind-up ratio and dynamic allodynia (5, 25, 559, 560). By comparing responses with large datasets that represent normal responses to sensory tests, neuropathic pain patients can be grouped into clusters based on their sensory profiles (5). The validity of this type of approach is supported by the observation that post-hoc analysis of responders to treatments in clinical trials suggest that clinical effectiveness may cluster according to pain phenotype (559).

Secondly, human microneurography techniques can now distinguish mechanosensitive C-fibres from non-mechanoceptive fibres in a given patient (542). It can also be used to detect spontaneous activity in nocceptors (561).

The multidimensional expereince of pain is a result of spatiotempotral patterns of brain activity wherein afferent nociceptive information is modulated by cognitive factors and from which descending modulatory systems control spinal level processing (25, 508). Analysis of electroencephalographic patterns (562) and non-invasive neuroimaging techniques such as paradigm free functional magnetic resonance imaging (fMRI) therefore offer the opportunity to examine the experience and chronification of pain in individual patients. Similarly, the use of positron emission tomography (PET) with radioligands targeting the 18 kDa translocator protein SPO has recently emerged as a technique for observing neuroinflammation and glial activation in patients *in vivo* (27).

Lastly, examination of expression of miRNAs in epidermis strongly discriminating pain patients from healthy individuals (563). In addition to providing a means to stratify patients prior to treatment, the above techniques also provide information concerning the effectiveness of therapeutic intervention.

#### Recognition of the importance of sex differences in human pain processing

The different mechanisms underlying neuropathic pain in females vs. males his has obvious therapeutic implications; drugs which are effective in men may be less effective in women and *vice-versa* (10, 361, 362, 365, 366, 564–569).

In support of this, recent work using resting-state magnetoencephalography has identified differential changes in patterns of brain oscillatory activity in males vs. females (570). Also a genome wide association study identified 123 single nucleotide polymorphisms (SNP) at 5 independent loci that are significantly associated with chronic pain in men. By contrast, 286 SNP's at 10 independent loci were identified in women (571). Gene-level analyses revealed sex-specific associations with chronic pain with 31 genes in females and 37 genes in males. All 37 chronic pain associated genes in men and 30/31 genes in women were expressed in DRG (571). In an extension of this, analysis of altered mRNA expression in the DRG of neuropathic pain patients also revealed profound sex differences in differentially expressed genes. Thus, message for IL-1β, TNF, CXCL-14 and OSM (Oncostatin M) were increased in males whereas CCL-1, CCL-21, PENK (proenkephalin A) and TLR3 (toll-like receptor 3) were increased in females. Cytokine signalling pathways associated with neuropathic pain in males included OSM, LIF, and SOCS1 (suppressor of cytokine signalling 1) whereas CCL-1, CCL-19 and CCL-21 were involved in females. Moreover,components of the JUN-FOS signalling pathway were enriched in males whereas genes coding centromere proteins were enriched in females (352).

### Molecular genetic techniques

Given the drawbacks of classical therapeutic approaches; drug toxicity, off target effects, drug interactions and in some cases drug dependence, there is a movement in all fields of medicine towards genetic rather than pharmacological approaches to disease management. Pain management is no exception to this trend (572).

By way of demonstration of the principle, CRISPR (clustered regularly interspaced short palindromic repeats) technology has been used in a mouse model to prevent expression of Na_v_1.7 by editing a regulatory sequence (573). This technology might have therapeutic potential in management of persistent pain states.in the clinic.

## Concluding statements

This review underlines the difficulty in translation between animal studies and the treatment of pain in the clinic. Although classical animal models have revealed many of the essential biological mechanisms that underlie neuropathic pain, such as peripheral and central sensitization and some of the molecular and cellular mechanisms involved, animal models do not adequately model the multiplicity of disease states or injuries that may bring about pain in humans. In terms of pharmacological management “one size does not fit all”, perhaps there will never be a panacea for neuropathic pain in the same way as opioids serve for most forms of nociceptive pain. Despite this, there is some cause for optimising treatment for individual patients by careful evaluation of the pain phenotype and delivering treatment accordingly.

## References

[1] Mogil JS. Sources of individual differences in pain. Ann Rev Neurosci. (2021) 44(1):1–25. 10.1146/annurev-neuro-092820-105941 34236890

[2] Moriarty O Tu Y Sengar AS Salter MW Beggs S Walker SM. Priming of adult incision response by early-life injury: neonatal microglial inhibition has persistent but sexually dimorphic effects in adult rats. J Neurosci. (2019) 39(16):3081–93. 10.1523/JNEUROSCI.1786-18.2019 30796159 PMC6468109

[3] Dworsky-Fried Z Kerr BJ Taylor AMW. Microbes, microglia, and pain. Neurobiol Pain. (2020) 7:100045. 10.1016/j.ynpai.2020.100045 32072077 PMC7016021

[4] Fitzgerald M McKelvey R. Nerve injury and neuropathic pain—A question of age. Exp Neurol. (2016) 275(Pt 2):296–302. 10.1016/j.expneurol.2015.07.013 26220898 PMC4691235

[5] Baron R Maier C Attal N Binder A Bouhassira D Cruccu G Peripheral neuropathic pain: a mechanism-related organizing principle based on sensory profiles. Pain. (2017) 158(2):261–72. 10.1097/j.pain.0000000000000753 27893485 PMC5266425

[6] Hastie BA Riley JL III Kaplan L Herrera DG Campbell CM Virtusio K Ethnicity interacts with the OPRM1 gene in experimental pain sensitivity. Pain. (2012) 153(8):1610–9. 10.1016/j.pain.2012.03.022 22717102 PMC3683662

[7] Ding W You Z Chen Q Yang L Doheny J Zhou X Gut Microbiota influences neuropathic pain through modulating proinflammatory and anti-inflammatory T cells. Anesth Analg. (2021) 132(4):1146–55. 10.1213/ANE.0000000000005155 32889847

[8] Edwards RR Schreiber KL Dworkin RH Turk DC Baron R Freeman R Optimizing and accelerating the development of precision pain treatments for chronic pain: IMMPACT review and recommendations. J Pain. (2023) 24(2):204–25. 10.1016/j.jpain.2022.08.010 36198371 PMC10868532

[9] Lee J Lee G Ko G Joong LS. Nerve injury-induced gut dysbiosis contributes to spinal cord TNF-alpha expression and nociceptive sensitization. Brain Behav Immun. (2023) 110:155–61. 10.1016/j.bbi.2023.03.005 36893921

[10] Ghazisaeidi S Muley MM Salter MW. Neuropathic pain: mechanisms, sex differences, and potential therapies for a global problem. Annu Rev Pharmacol Toxicol. (2023) 63:565–83. 10.1146/annurev-pharmtox-051421-112259 36662582

[11] Fiore NT Debs SR Hayes JP Duffy SS Moalem-Taylor G. Pain-resolving immune mechanisms in neuropathic pain. Nat Rev Neurol. (2023) 19(4):199–220. 10.1038/s41582-023-00777-3 36859719

[12] Bannister K Sachau J Baron R Dickenson AH. Neuropathic pain: mechanism-based therapeutics. Annu Rev Pharmacol Toxicol (2020) 60:257–74. 10.1146/annurev-pharmtox-010818-021524 31914896

[13] Wall PD Devor M Inbal R Scadding JW Schonfeld D Seltzer Z Autotomy following peripheral nerve lesions: experimental anaesthesia dolorosa. Pain. (1979) 7:103–13. 10.1016/0304-3959(79)90002-2 574931

[14] Finnerup NB Kuner R Jensen TS. Neuropathic pain: from mechanisms to treatment. Physiol Rev. (2021) 101(1):259–301. 10.1152/physrev.00045.2019 32584191

[15] Colloca L Ludman T Bouhassira D Baron R Dickenson AH Yarnitsky D Neuropathic pain. Nat Rev Dis Primers. (2017) 3:17002. 10.1038/nrdp.2017.2 28205574 PMC5371025

[16] Finnerup NB Attal N Haroutounian S McNicol E Baron R Dworkin RH Pharmacotherapy for neuropathic pain in adults: a systematic review and meta-analysis. Lancet Neurol. (2015) 14:162–73. 10.1016/S1474-4422(14)70251-0 25575710 PMC4493167

[17] Yekkirala AS Roberson DP Bean BP Woolf CJ. Breaking barriers to novel analgesic drug development. Nat Rev Drug Discov. (2017) 16:545–64. 10.1038/nrd.2017.87 28596533 PMC5675565

[18] Scholz J Woolf CJ. The neuropathic pain triad: neurons, immune cells and glia. Nat Neurosci. (2007) 10(11):1361–8. 10.1038/nn1992 17965656

[19] Walters ET Crook RJ Neely GG Price TJ Smith ESJ. Persistent nociceptor hyperactivity as a painful evolutionary adaptation. Trends Neurosci. (2023) 46(3):211–27. 10.1016/j.tins.2022.12.007 36610893 PMC9974896

[20] Costigan M Scholz J Woolf CJ. Neuropathic pain: a maladaptive response of the nervous system to damage. Annu Rev Neurosci. (2009) 32:1–32. 10.1146/annurev.neuro.051508.135531 19400724 PMC2768555

[21] Iadarola MJ Caudle RM. Good pain, bad pain. Science. (1997) 278:239–40. 10.1126/science.278.5336.239 9340772

[22] Alles SRA Smith PA. The etiology and pharmacology of neuropathic pain. Pharmacol Rev. (2018) 70:315–47. 10.1124/pr.117.014399 29500312

[23] Gold MS Gebhart GF. Nociceptor sensitization in pain pathogenesis. Nat Med. (2010) 16(11):1248–57. 10.1038/nm.2235 20948530 PMC5022111

[24] Schwartz SM Barpujari A Finnerup NB Raja SN. Pharmacologic therapies for neuropathic pain: an assessment of reporting biases in randomized controlled trials. Pain. (2022) 163(4):795–804. 10.1097/j.pain.0000000000002426 34348355 PMC8810900

[25] Soliman N Kersebaum D Lawn T Sachau J Sendel M Vollert J. Improving neuropathic pain treatment - by rigorous stratification from bench to bedside. J Neurochem. (2023). 10.1111/jnc.15798. [Epub ahead of print] 36852505

[26] Gormsen L Rosenberg R Bach FW Jensen TS. Depression, anxiety, health-related quality of life and pain in patients with chronic fibromyalgia and neuropathic pain. Eur J Pain. (2010) 14(2):127–8. 10.1016/j.ejpain.2009.03.010 19473857

[27] Grace PM Tawfik VL Svensson CI Burton MD Loggia ML Hutchinson MR. The neuroimmunology of chronic pain: from rodents to humans. J Neurosci. (2021) 41(5):855. 10.1523/JNEUROSCI.1650-20.2020 33239404 PMC7880288

[28] Waxman SG. Peripheral afferents and the pain experience. Pain. (2019) 160(7):1487–8. 10.1097/j.pain.0000000000001527 30939586

[29] Gangadharan V Zheng H Taberner FJ Landry J Nees TA Pistolic J Neuropathic pain caused by miswiring and abnormal end organ targeting. Nature. (2022) 606(7912):137–45. 10.1038/s41586-022-04777-z 35614217 PMC9159955

[30] Vaso A Adahan HM Gjika A Zahaj S Zhurda T Vyshka G Peripheral nervous system origin of phantom limb pain. Pain. (2014) 155(7):1384–91. 10.1016/j.pain.2014.04.018 24769187

[31] Carlton SM Du J Tan HY Nesic O Hargett GL Bopp AC Peripheral and central sensitization in remote spinal cord regions contribute to central neuropathic pain after spinal cord injury. Pain. (2009) 147(1–3):265–76. 10.1016/j.pain.2009.09.030 19853381 PMC2787843

[32] Wu J Renn CL Faden AI Dorsey SG. TrkB.T1 contributes to neuropathic pain after spinal cord injury through regulation of cell cycle pathways. J Neurosci. (2013) 33(30):12447–63. 10.1523/JNEUROSCI.0846-13.2013 23884949 PMC3721848

[33] Yousuf MS Noh MC Friedman TN Zubkow K Johnson JC Tenorio G Sensory neurons of the dorsal root ganglia become hyperexcitable in a T-cell-mediated MOG-EAE model of multiple sclerosis. eNeuro. (2019) 6(2):ENEURO.0024–19.2019. 10.1523/ENEURO.0024-19.2019 30957012 PMC6449162

[34] Olechowski CJ Truong JJ Kerr BJ. Neuropathic pain behaviours in a chronic-relapsing model of experimental autoimmune encephalomyelitis (EAE). Pain. (2009) 141(1–2):156–64. 10.1016/j.pain.2008.11.002 19084337

[35] Haroutounian S Ford AL Frey K Nikolajsen L Finnerup NB Neiner A How central is central poststroke pain? The role of afferent input in poststroke neuropathic pain: a prospective, open-label pilot study. Pain. (2018) 159(7):1317–24. 10.1097/j.pain.0000000000001213 29570507

[36] Staud R. Fibromyalgia pain: do we know the source? Curr Opin Rheumatol. (2004) 16(2):157–63. 10.1097/00002281-200403000-00016 14770104

[37] Sumpton JE Moulin DE. Fibromyalgia. Handb Clin Neurol. (2014) 119:513–27. 10.1016/B978-0-7020-4086-3.00033-3 24365316

[38] Kress L Egenolf N Sommer C Uceyler N. Cytokine expression profiles in white blood cells of patients with small fiber neuropathy. BMC Neurosci. (2023) 24(1):1. 10.1186/s12868-022-00770-4 36604634 PMC9817338

[39] Zak-Prelich M McKenzie RC Sysa-Jedrzejowska A Norval M. Local immune responses and systemic cytokine responses in zoster: relationship to the development of postherpetic neuralgia. Clin Exp Immunol. (2003) 131(2):318–23. 10.1046/j.1365-2249.2003.02061.x 12562395 PMC1808626

[40] Cregg R Momin A Rugiero F Wood JN Zhao J. Pain channelopathies. J Physiol. (2010) 588(Pt 11):1897–904. 10.1113/jphysiol.2010.187807 20142270 PMC2901978

[41] Lu VB Smith PA Rashiq S. The excitability of dorsal horn neurons is affected by cerebrospinal fluid from humans with osteoarthritis. Can J Physiol Pharmacol. (2012) 90(6):783–90. 10.1139/y2012-014 22506885

[42] Rahman W Patel R Dickenson AH. Electrophysiological evidence for voltage-gated calcium channel 2 (Cav2) modulation of mechano- and thermosensitive spinal neuronal responses in a rat model of osteoarthritis. Neuroscience. (2015) 305:76–85. 10.1016/j.neuroscience.2015.07.073 26247695 PMC4564012

[43] Jensen MP Chodroff MJ Dworkin RH. The impact of neuropathic pain on health-related quality of life: review and implications. Neurology. (2007) 68(15):1178–82. 10.1212/01.wnl.0000259085.61898.9e 17420400

[44] McLachlan EM Janig W Michalis M. Peripheral nerve injury triggers noradrenergic sprouting within dorsal root ganglia. Nature. (1993) 363:543–6. 10.1038/363543a0 8505981

[45] Rifbjerg-Madsen S Christensen AW Christensen R Hetland ML Bliddal H Kristensen LE Pain and pain mechanisms in patients with inflammatory arthritis: a danish nationwide cross-sectional DANBIO registry survey. PLoS One. (2017) 12(7):e0180014. 10.1371/journal.pone.0180014 28686639 PMC5501437

[46] Zochodne DW. Neurotrophins and other growth factors in diabetic neuropathy. Semin Neurol. (1996) 16(2):153–61. 10.1055/s-2008-1040971 8987129

[47] Bouali-Benazzouz R Landry M Benazzouz A Fossat P. Neuropathic pain modeling: focus on synaptic and ion channel mechanisms. Prog Neurobiol. (2021) 201:102030. 10.1016/j.pneurobio.2021.102030 33711402

[48] Mifflin KA Kerr BJ. Pain in autoimmune disorders. J Neurosci Res. (2017) 95(6):1282–94. 10.1002/jnr.23844 27448322

[49] Acharjee S Noorbakhsh F Stemkowski PL Olechowski C Cohen EA Ballanyi K HIV-1 viral protein R causes peripheral nervous system injury associated with in vivo neuropathic pain. FASEB J. (2010) 24(11):4343–53. 10.1096/fj.10-162313 20628092

[50] Shi Y Gelman BB Lisinicchia JG Tang SJ. Chronic-pain-associated astrocytic reaction in the spinal cord dorsal horn of human immunodeficiency virus-infected patients. J Neurosci. (2012) 32(32):10833–40. 10.1523/JNEUROSCI.5628-11.2012 22875918 PMC3470811

[51] Ru W Liu X Bae C Shi Y Walikonis R Mo CJ Microglia mediate HIV-1 gp120-induced synaptic degeneration in spinal pain neural circuits. J Neurosci. (2019) 39(42):8408–21. 10.1523/JNEUROSCI.2851-18.2019 31471472 PMC6794928

[52] Attal N Martinez V Bouhassira D. Potential for increased prevalence of neuropathic pain after the COVID-19 pandemic. Pain Rep. (2021) 6(1):e884. 10.1097/PR9.0000000000000884 33537521 PMC7850724

[53] Descoeur J Pereira V Pizzoccaro A Francois A Ling B Maffre V Oxaliplatin-induced cold hypersensitivity is due to remodelling of ion channel expression in nociceptors. EMBO Mol Med. (2011) 3(5):266–78. 10.1002/emmm.201100134 21438154 PMC3377073

[54] Xiao W Boroujerdi A Bennett GJ Luo ZD. Chemotherapy-evoked painful peripheral neuropathy: analgesic effects of gabapentin and effects on expression of the alpha-2-delta type-1 calcium channel subunit. Neuroscience. (2007) 144(2):714–20. 10.1016/j.neuroscience.2006.09.044 17084535 PMC1805704

[55] Flatters SJL Bennett GJ. Studies of peripheral sensory nerves in paclitaxel-induced painful peripheral neuropathy: evidence for mitochondrial dysfunction. Pain. (2006) 122(3):245–57. 10.1016/j.pain.2006.01.037 16530964 PMC1805481

[56] Gibson CA. Review of posttraumatic stress disorder and chronic pain: the path to integrated care. J Rehabil Res Dev. (2012) 49(5):753–76. 10.1682/JRRD.2011.09.0158 23015584

[57] Cummins TR Dib-Hajj SD Waxman SG. Electrophysiological properties of mutant Nav1.7 sodium channels in a painful inherited neuropathy. J Neurosci. (2004) 24(38):8232–6. 10.1523/JNEUROSCI.2695-04.2004 15385606 PMC6729696

[58] Dib-Hajj SD Rush AM Cummins TR Hisama FM Novella S Tyrrell L Gain-of-function mutation in Nav1.7 in familial erythromelalgia induces bursting of sensory neurons. Brain. (2005) 128(Pt 8):1847–54. 10.1093/brain/awh514 15958509

[59] Dib-Hajj SD Waxman SG. Sodium channels in human pain disorders: genetics and pharmacogenomics. Annu Rev Neurosci. (2019) 42:87–106. 10.1146/annurev-neuro-070918-050144 30702961

[60] Stemkowski PL Smith PA. An overview of animal models of neuropathic pain. In: Toth C Moulin DE, editors. Neuropathic pain, causes, management and understanding. Cambridge: Cambridge University Press (2013). p. 33–50.

[61] Decosterd I Woolf CJ. Spared nerve injury: an animal model of persistent peripheral neuropathic pain. Pain. (2000) 87(2):149–58. 10.1016/S0304-3959(00)00276-1 10924808

[62] Kim KJ Yoon YW Chung JM. Comparison of three rodent models of neuropathic pain. Exp Brain Res. (1997) 113:200–6. 10.1007/BF02450318 9063706

[63] Natale CA Christie MJ Aubrey KR. Spinal glycinergic currents are reduced in a rat model of neuropathic pain following partial nerve ligation but not chronic constriction injury. J Neurophysiol. (2023) 129(2):333–41. 10.1152/jn.00451.2022 36541621

[64] Price TJ Basbaum AI Bresnahan J Chambers JF De KY Edwards RR Transition to chronic pain: opportunities for novel therapeutics. Nat Rev Neurosci. (2018) 19(7):383–4. 10.1038/s41583-018-0012-5 29765159 PMC6237656

[65] Rice ASC Finnerup NB Kemp HI Currie GL Baron R. Sensory profiling in animal models of neuropathic pain: a call for back-translation. Pain. (2018) 159(5):819–24. 10.1097/j.pain.0000000000001138 29300280 PMC5911154

[66] Schulte A Lohner H Degenbeck J Segebarth D Rittner HL Blum R Unbiased analysis of the dorsal root anglion after peripheral nerve injury: no neuronal loss, no gliosis, but satellite glial cell plasticity. Pain. (2023) 164(4):728–40. 10.1097/j.pain.0000000000002758 35969236 PMC10026836

[67] Radtke C Vogt PM Devor M Kocsis JD. Keratinocytes acting on injured afferents induce extreme neuronal hyperexcitability and chronic pain. Pain. (2010) 148(1):94–102. 10.1016/j.pain.2009.10.014 19932564

[68] Boakye PA Tang SJ Smith PA. Mediators of neuropathic pain; focus on spinal microglia, CSF-1, BDNF, CCL21, TNF-alpha, Wnt Ligands, and Interleukin 1-beta. Front Pain Res. (2021) 2:698157. 10.3389/fpain.2021.698157 PMC8915739 35295524

[69] McMahon SB Cafferty WB Marchand F. Immune and glial cell factors as pain mediators and modulators. Exp Neurol. (2005) 192(2):444–62. 10.1016/j.expneurol.2004.11.001 15755561

[70] Moalem G Tracey DJ. Immune and inflammatory mechanisms in neuropathic pain. Brain Res Rev. (2006) 51(2):240–64. 10.1016/j.brainresrev.2005.11.004 16388853

[71] Yu X Liu H Hamel KA Morvan MG Yu S Leff J Dorsal root ganglion macrophages contribute to both the initiation and persistence of neuropathic pain. Nat Commun. (2020) 11(1):264. 10.1038/s41467-019-13839-2 31937758 PMC6959328

[72] Noh MC Mikler B Joy T Smith PA. Time course of inflammation in dorsal root ganglia correlates with differential reversibility of mechanical allodynia. Neuroscience. (2020) 428:199–216. 10.1016/j.neuroscience.2019.12.040 31918012

[73] Sommer C Petrausch S Lindenlaub T Toyka KV. Neutralizing antibodies to interleukin 1-receptor reduce pain associated behavior in mice with experimental neuropathy. Neurosci Lett. (1999) 270(1):25–8. 10.1016/S0304-3940(99)00450-4 10454137

[74] Zelenka M Schafers M Sommer C. Intraneural injection of interleukin-1beta and tumor necrosis factor-alpha into rat sciatic nerve at physiological doses induces signs of neuropathic pain. Pain. (2005) 116(3):257–63. 10.1016/j.pain.2005.04.018 15964142

[75] Wolf G Gabay E Tal M Yirmiya R Shavit Y. Genetic impairment of interleukin-1 signaling attenuates neuropathic pain, autotomy, and spontaneous ectopic neuronal activity, following nerve injury in mice. Pain. (2006) 120(3):315–24. 10.1016/j.pain.2005.11.011 16426759

[76] Gabay E Wolf G Shavit Y Yirmiya R Tal M. Chronic blockade of interleukin-1 (IL-1) prevents and attenuates neuropathic pain behavior and spontaneous ectopic neuronal activity following nerve injury. Eur J Pain. (2011) 15(3):242–8. 10.1016/j.ejpain.2010.07.012 20801063

[77] Kleinschnitz C Hofstetter HH Meuth SG Braeuninger S Sommer C Stoll G. T cell infiltration after chronic constriction injury of mouse sciatic nerve is associated with interleukin-17 expression. Exp Neurol. (2006) 200(2):480–5. 10.1016/j.expneurol.2006.03.014 16674943

[78] Gomez-Nicola D Valle-Argos B Suardiaz M Taylor JS Nieto-Sampedro M. Role of IL-15 in spinal cord and sciatic nerve after chronic constriction injury: regulation of macrophage and T-cell infiltration. J Neurochem. (2008) 107(6):1741–52. 10.1111/j.1471-4159.2008.05746.x 19014377

[79] Kim CF Moalem-Taylor G. Interleukin-17 contributes to neuroinflammation and neuropathic pain following peripheral nerve injury in mice. J Pain. (2011) 12(3):370–83. 10.1016/j.jpain.2010.08.003 20889388

[80] Vasudeva K Vodovotz Y Azhar N Barclay D Janjic JM Pollock JA. In vivo and systems biology studies implicate IL-18 as a central mediator in chronic pain. J Neuroimmunol. (2015) 283:43–9. 10.1016/j.jneuroim.2015.04.012 26004155 PMC4465386

[81] Shamash S Reichert F Rotshenker S. The cytokine network of wallerian degeneration: tumor necrosis factor-alpha, interleukin-1alpha, and interleukin-1beta. J Neurosci. (2002) 22(8):3052–60. 10.1523/JNEUROSCI.22-08-03052.2002 11943808 PMC6757534

[82] Khan J Hassun H Zusman T Korczeniewska O Eliav E. Interleukin-8 levels in rat models of nerve damage and neuropathic pain. Neurosci Lett. (2017) 657:106–12. 10.1016/j.neulet.2017.07.049 28789985

[83] Dubovy P Klusakova I Svizenska I Brazda V. Satellite glial cells express IL-6 and corresponding signal-transducing receptors in the dorsal root ganglia of rat neuropathic pain model. Neuron Glia Biol. (2010) 6(1):73–83. 10.1017/S1740925X10000074 20519054

[84] Day YJ Liou JT Lee CM Lin YC Mao CC Chou AH Lack of interleukin-17 leads to a modulated micro-environment and amelioration of mechanical hypersensitivity after peripheral nerve injury in mice. Pain. (2014) 155(7):1293–302. 10.1016/j.pain.2014.04.004 24721689

[85] Leung L Cahill CM. TNF-alpha and neuropathic pain–a review. J Neuroinflammation. (2010) 7:27. 10.1186/1742-2094-7-27 20398373 PMC2861665

[86] Bastien D Lacroix S. Cytokine pathways regulating glial and leukocyte function after spinal cord and peripheral nerve injury. Exp Neurol. (2014) 258:62–77. 10.1016/j.expneurol.2014.04.006 25017888

[87] Thompson SW Dray A Urban L. Leukemia inhibitory factor induces mechanical allodynia but not thermal hyperalgesia in the juvenile rat. Neuroscience. (1996) 71(4):1091–4. 10.1016/0306-4522(95)00537-4 8684613

[88] Mwirigi J Franco-Enzastiga UM Sankaranarayanan I Tavares-Ferreira D Shiers SI Ray P Oncostatin M induces nociceptive signaling in human dorsal root ganglia. J Pain. (2023) 24(4, Supplement):16. 10.1016/j.jpain.2023.02.061

[89] Pezet S McMahon SB. Neurotrophins: mediators and modulators of pain. Ann Rev Neurosci. (2006) 29(1):507–38. 10.1146/annurev.neuro.29.051605.112929 16776595

[90] Tanaka T Okuda H Isonishi A Terada Y Kitabatake M Shinjo T Dermal macrophages set pain sensitivity by modulating the amount of tissue NGF through an SNX25-Nrf2 pathway. Nat Immunol. (2023) 24(3):439–51. 10.1038/s41590-022-01418-5 36703006 PMC9977679

[91] Kaur G Singh N Jaggi AS. Mast cells in neuropathic pain: an increasing spectrum of their involvement in pathophysiology. Rev Neurosci. (2017) 28(7):759–66. 10.1515/revneuro-2017-0007 28688228

[92] Obara I Telezhkin V Alrashdi I Chazot PL. Histamine, histamine receptors, and neuropathic pain relief. Br J Pharmacol. (2020) 177(3):580–99. 10.1111/bph.14696 31046146 PMC7012972

[93] Khalilzadeh E Azarpey F Hazrati R Vafaei SG. Evaluation of different classes of histamine H1 and H2 receptor antagonist effects on neuropathic nociceptive behavior following tibial nerve transection in rats. Eur J Pharmacol. (2018) 834:221–9. 10.1016/j.ejphar.2018.07.011 30009812

[94] Wei T Guo TZ Li WW Hou S Kingery WS Clark JD. Keratinocyte expression of inflammatory mediators plays a crucial role in substance P-induced acute and chronic pain. J Neuroinflammation. (2012) 9:181. 10.1038/s41590-022-01418-5 22824437 PMC3458986

[95] Simonetti M Agarwal N Stosser S Bali KK Karaulanov E Kamble R Wnt-Fzd signaling sensitizes peripheral sensory neurons via distinct noncanonical pathways. Neuron. (2014) 83(1):104–21. 10.1016/j.neuron.2014.05.037 24991956

[96] van Vliet AC Lee J van der Poel M Mason MRJ Noordermeer JN Fradkin LG Coordinated changes in the expression of wnt pathway genes following human and rat peripheral nerve injury. PLoS One. (2021) 16(4):e0249748. 10.1371/journal.pone.0249748 33848304 PMC8043392

[97] White FA Wilson NM. Chemokines as pain mediators and modulators. Curr Opin Anaesthesiol. (2008) 21(5):580–5. 10.1097/ACO.0b013e32830eb69d 18784482 PMC2702665

[98] Pawlik K Piotrowska A Kwiatkowski K Ciapala K Popiolek-Barczyk K Makuch W The blockade of CC chemokine receptor type 1 influences the level of nociceptive factors and enhances opioid analgesic potency in a rat model of neuropathic pain. Immunology. (2020) 159(4):413–28. 10.1111/imm.13172 31919846 PMC7078003

[99] Xian H Jiang Y Zhang H Ma SB Zhao R Cong R. CCL2-CCR2 Axis potentiates NMDA receptor signaling to aggravate neuropathic pain induced by brachial Plexus avulsion. Neuroscience. (2020) 425:29–38. 10.1016/j.neuroscience.2019.11.012 31805255

[100] Moalem G Grafe P Tracey DJ. Chemical mediators enhance the excitability of unmyelinated sensory axons in normal and injured peripheral nerve of the rat. Neuroscience. (2005) 134(4):1399–411. 10.1016/j.neuroscience.2005.05.046 16039795

[101] Jayaraj ND Bhattacharyya BJ Belmadani AA Ren D Rathwell CA Hackelberg S Reducing CXCR4-ediated nociceptor hyperexcitability reverses painful diabetic neuropathy. J Clin Invest. (2018) 128(6):2205–25. 10.1172/JCI92117 29533926 PMC5983349

[102] Dubovy P Klusakova I Svizenska I Brazda V. Spatio-temporal changes of SDF1 and its CXCR4 receptor in the dorsal root ganglia following unilateral sciatic nerve injury as a model of neuropathic pain. Histochem Cell Biol. (2010) 133(3):323–37. 10.1007/s00418-010-0675-0 20127490

[103] Bai L Wang X Li Z Kong C Zhao Y Qian JL Upregulation of chemokine CXCL12 in the dorsal root ganglia and spinal cord contributes to the development and maintenance of neuropathic pain following spared nerve injury in rats. Neurosci Bull. (2016) 32(1):27–40. 10.1007/s12264-015-0007-4 26781879 PMC5563752

[104] Yu Y Huang X Di Y Qu L Fan N. Effect of CXCL12/CXCR4 signaling on neuropathic pain after chronic compression of dorsal root ganglion. Sci Rep. (2017) 7(1):5707. 10.1038/s41598-017-05954-1 28720830 PMC5515923

[105] Kawano T Zoga V Kimura M Liang MY Wu HE Gemes G Nitric oxide activates ATP-sensitive potassium channels in mammalian sensory neurons: action by direct S-nitrosylation. Mol Pain. (2009) 5:12. 10.1186/1744-8069-5-12 19284878 PMC2673211

[106] Renganathan M Cummins TR Waxman SG. Nitric oxide blocks fast, slow, and persistent na+ channels in C-type DRG neurons by S-nitrosylation. J Neurophysiol. (2002) 87(2):761–75. 10.1152/jn.00369.2001 11826045

[107] Renganathan M Cummins TR Hormuzdiar WN Black JA Waxman SG. Nitric oxide is an autocrine regulator of na(+) currents in axotomized C-type DRG neurons. J Neurophysiol. (2000) 83(4):2431–42. 10.1152/jn.2000.83.4.2431 10758144

[108] Grace PM Gaudet AD Staikopoulos V Maier SF Hutchinson MR Salvemini D Nitroxidative signaling mechanisms in pathological pain. Trends Neurosci. (2016) 39(12):862–79. 10.1016/j.tins.2016.10.003 27842920 PMC5148691

[109] George DS Hackelberg S Jayaraj ND Ren D Edassery SL Rathwell CA Mitochondrial calcium uniporter deletion prevents painful diabetic neuropathy by restoring mitochondrial morphology and dynamics. Pain. (2022) 163(3):560–78. 10.1097/j.pain.0000000000002391 34232927 PMC8720329

[110] Guan Z Kuhn JA Wang X Colquitt B Solorzano C Vaman S Injured sensory neuron-derived CSF1 induces microglial proliferation and DAP12-dependent pain. Nat Neurosci. (2016) 19(1):94–101. 10.1038/nn.4189 26642091 PMC4703328

[111] Chitu V Gokhan S Nandi S Mehler MF Stanley ER. Emerging roles for CSF-1 receptor and its ligands in the nervous system. Trends Neurosci. (2016) 39(6):378–93. 10.1016/j.tins.2016.03.005 27083478 PMC4884457

[112] Okubo M Yamanaka H Kobayashi K Dai Y Kanda H Yagi H Macrophage-Colony stimulating factor derived from injured primary afferent induces proliferation of spinal microglia and neuropathic pain in rats. PLoS One. (2016) 11(4):e0153375. 10.1371/journal.pone.0153375 27071004 PMC4829214

[113] Boakye PA Rancic V Whitlock KH Simmons D Longo FM Ballanyi K Receptor dependence of BDNF actions in superficial dorsal horn: relation to central sensitization and actions of macrophage colony stimulating factor 1. J Neurophysiol. (2019) 121(6):2308–22. 10.1152/jn.00839.2018 30995156

[114] Piotrowska A Rojewska E Pawlik K Kreiner G Ciechanowska A Makuch W Pharmacological blockade of CXCR3 by (+/-)-NBI-74330 reduces neuropathic pain and enhances opioid effectiveness - evidence from in vivo and in vitro studies. Biochim Biophys Acta Mol Basis Dis. (2018) 1864(10):3418–37. 10.1016/j.bbadis.2018.07.032 30076959

[115] Biber K Tsuda M Tozaki-Saitoh H Tsukamoto K Toyomitsu E Masuda T Neuronal CCL21 up-regulates microglia P2X4 expression and initiates neuropathic pain development. EMBO J. (2011) 30(9):1864–73. 10.1038/emboj.2011.89 21441897 PMC3101996

[116] Schmitz K Pickert G Wijnvoord N Haussler A Tegeder I. Dichotomy of CCL21 and CXCR3 in nerve injury-evoked and autoimmunity-evoked hyperalgesia. Brain Behav Immun. (2013) 32:186–200. 10.1016/j.bbi.2013.04.011 23643685

[117] Biggs JE Lu VB Stebbing MJ Balasubramanyan S Smith PA. Is BDNF sufficient for information transfer between microglia and dorsal horn neurons during the onset of central sensitization? Mol Pain. (2010) 6:44. 10.1186/1744-8069-6-44 20653959 PMC2918544

[118] Trang T Beggs S Wan X Salter MW. P2X4-receptor-mediated synthesis and release of brain-derived neurotrophic factor in microglia is dependent on calcium and p38-mitogen-activated protein kinase activation. J Neurosci. (2009) 29(11):3518–28. 10.1523/JNEUROSCI.5714-08.2009 19295157 PMC3589565

[119] Trang T Beggs S Salter MW. Brain-derived neurotrophic factor from microglia: a molecular substrate for neuropathic pain. Neuron Glia Biol. (2011) 7(1):99–108. 10.1017/S1740925X12000087 22613083 PMC3748035

[120] Ulmann L Hatcher JP Hughes JP Chaumont S Green PJ Conquet F Up-regulation of P2X4 receptors in spinal microglia after peripheral nerve injury mediates BDNF release and neuropathic pain. J Neurosci. (2008) 28(44):11263–8. 10.1523/JNEUROSCI.2308-08.2008 18971468 PMC6671487

[121] Coull JA Beggs S Boudreau D Boivin D Tsuda M Inoue K BDNF From microglia causes the shift in neuronal anion gradient underlying neuropathic pain. Nature. (2005) 438(7070):1017-21. 10.1038/nature04223 16355225

[122] Lu VB Biggs JE Stebbing MJ Balasubramanyan S Todd KG Lai AY BDNF Drives the changes in excitatory synaptic transmission in the rat superficial dorsal horn that follow sciatic nerve injury. J Physiol (Lond). (2009) 587:1013–32. 10.1113/jphysiol.2008.166306 19124536 PMC2673772

[123] Smith PA. BDNF: no gain without pain? Neuroscience. (2014) 283:107–23. 10.1016/j.neuroscience.2014.05.044 24887639

[124] Simonetti M Kuner R. Spinal Wnt5a plays a key role in spinal dendritic spine remodeling in neuropathic and inflammatory pain models and in the proalgesic effects of peripheral Wnt3a. J Neurosci. (2020) 40(35):6664. 10.1523/JNEUROSCI.2942-19.2020 32616667 PMC7455212

[125] Silva R Malcangio M. Fractalkine/CX3CR1 pathway in neuropathic pain: an update. Front Pain Res. (2021) 2:35. 10.3389/fpain.2021.684684 PMC8915718 35295489

[126] Clark AK Malcangio M. Microglial signalling mechanisms: cathepsin S and fractalkine. Exp Neurol. (2012) 234(2):283–92. 10.1016/j.expneurol.2011.09.012 21946268

[127] Clark AK Malcangio M. Fractalkine/CX3CR1 signaling during neuropathic pain. Front Cell Neurosci (2014)8:121. 10.3389/fncel.2014.00121 24847207 PMC4019858

[128] Jung H Bhangoo S Banisadr G Freitag C Ren D White FA Visualization of chemokine receptor activation in transgenic mice reveals peripheral activation of CCR2 receptors in states of neuropathic pain. J Neurosci. (2009) 29(25):8051–62. 10.1523/JNEUROSCI.0485-09.2009 19553445 PMC3097108

[129] Al-Mazidi S Alotaibi M Nedjadi T Chaudhary A Alzoghaibi M Djouhri L. Blocking of cytokines signalling attenuates evoked and spontaneous neuropathic pain behaviours in the paclitaxel rat model of chemotherapy-induced neuropathy. Eur J Pain. (2018) 22(4):810–21. 10.1002/ejp.1169 29282807

[130] Luo X Tai WL Sun L Qiu Q Xia Z Chung SK Central administration of C-X-C chemokine receptor type 4 antagonist alleviates the development and maintenance of peripheral neuropathic pain in mice. PLoS One. (2014) 9(8):e104860. 10.1371/journal.pone.0104860 25119456 PMC4132096

[131] Barragan-Iglesias P Franco-Enzastiga U Jeevakumar V Shiers S Wangzhou A Granados-Soto V Type I interferons act directly on nociceptors to produce pain sensitization: implications for viral infection-induced pain. J Neurosci. (2020) 40(18):3517–32. 10.1523/JNEUROSCI.3055-19.2020 32245829 PMC7189756

[132] Reischer G Heinke B Sandkuhler J. Interferon gamma facilitates the synaptic transmission between primary afferent C-fibres and lamina I neurons in the rat spinal dorsal horn via microglia activation. Mol Pain. (2020) 16:1744806920917249. 10.1177/1744806920917249 32264753 PMC7144669

[133] Luo H Liu HZ Zhang WW Matsuda M Lv N Chen G Interleukin-17 regulates neuron-glial communications, synaptic transmission, and neuropathic pain after chemotherapy. Cell Rep. (2019) 29(8):2384–97. 10.1016/j.celrep.2019.10.085 31747607

[134] Sun JL Dai WJ Shen XY Lu N Zhang YQ. Interleukin-17 is involved in neuropathic pain and spinal synapse plasticity on mice. J Neuroimmunol. (2023) 377:578068. 10.1016/j.jneuroim.2023.578068 36948094

[135] Binshtok AM Wang H Zimmermann K Amaya F Vardeh D Shi L Nociceptors are interleukin-1{beta} sensors. J Neurosci. (2008) 28(52):14062–73. 10.1523/JNEUROSCI.3795-08.2008 19109489 PMC2690713

[136] Stemkowski PL Smith PA. Long-term IL-1beta exposure causes subpopulation-dependent alterations in rat dorsal root ganglion neuron excitability. J Neurophysiol. (2012) 107(6):1586–97. 10.1152/jn.00587.2011 22170966

[137] Stemkowski PL Noh MC Chen Y Smith PA. Increased excitability of medium-sized dorsal root ganglion neurons by prolonged interleukin-1beta exposure is K(+) channel dependent and reversible. J Physiol. (2015) 593(16):3739–55. 10.1113/JP270905 26110238 PMC4560594

[138] Noh MC Stemkowski PL Smith PA. Long-term actions of interleukin-1beta on K(+), Na(+) and Ca(2+) channel currents in small, IB4-positive dorsal root ganglion neurons; possible relevance to the etiology of neuropathic pain. J Neuroimmunol. (2019) 332:198–211. 10.1016/j.jneuroim.2019.05.002 31077855

[139] Nadeau S Filali M Zhang J Kerr BJ Rivest S Soulet D Functional recovery after peripheral nerve injury is dependent on the pro-inflammatory cytokines IL-1beta and TNF: implications for neuropathic pain. J Neurosci. (2011) 31(35):12533–42. 10.1523/JNEUROSCI.2840-11.2011 21880915 PMC6703268

[140] Stemkowski PL Garcia-Caballero A Gadotti VM MΓÇÖDahoma S Chen L Souza IA Identification of interleukin-1 beta as a key mediator in the upregulation of Cav3.2*Γ*ÇôUSP5 interactions in the pain pathway. Mol Pain. (2017) 13:1744806917724698. 10.1177/1744806917724698 28741432 PMC5560507

[141] Webster CI Hatcher J Burrell M Thom G Thornton P Gurrell I Enhanced delivery of IL-1 receptor antagonist to the central nervous system as a novel anti-transferrin receptor-IL-1RA fusion reverses neuropathic mechanical hypersensitivity. Pain. (2017) 158(4):660–8. 10.1097/j.pain.0000000000000810 28009628 PMC5359788

[142] Yamashita T Kamikaseda S Tanaka A Tosaki-Saitoh H Caaveiro J Inoue K New inhibitory effects of cilnidipine on microglial P2X7 receptors and IL-1 beta release: an involvement in its alleviating effect on neuropathic pain. Cells. (2021) 10:434. 33670748 10.3390/cells10020434 PMC7922706

[143] Gustafson-Vickers SL Lu VB Lai AY Todd KG Ballanyi K Smith PA. Long-term actions of interleukin-1beta on delay and tonic firing neurons in rat superficial dorsal horn and their relevance to central sensitization. Mol Pain. (2008) 4:63. 10.1186/1744-8069-4-63 19091115 PMC2625335

[144] Kawasaki Y Zhang L Cheng JK Ji RR. Cytokine mechanisms of central sensitization: distinct and overlapping role of interleukin-1beta, interleukin-6, and tumor necrosis factor-alpha in regulating synaptic and neuronal activity in the superficial spinal cord. J Neurosci. (2008) 28(20):5189–94. 10.1523/JNEUROSCI.3338-07.2008 18480275 PMC2408767

[145] Thompson SW Majithia AA. Leukemia inhibitory factor induces sympathetic sprouting in intact dorsal root ganglia in the adult rat in vivo. J Physiol (Lond). (1998) 506 (Pt 3):809–16. 10.1111/j.1469-7793.1998.809bv.x 9503339 PMC2230752

[146] Lopez ER Carbajal AG Tian JB Bavencoffe A Zhu MX Dessauer CW Serotonin enhances depolarizing spontaneous fluctuations, excitability, and ongoing activity in isolated rat DRG neurons via 5-HT4 receptors and cAMP-dependent mechanisms. Neuropharmacology. (2021) 184:108408. 10.1016/j.neuropharm.2020.108408 33220305 PMC7856035

[147] Ma C Greenquist KW LaMotte RH. Inflammatory mediators enhance the excitability of chronically compressed dorsal root ganglion neurons. J Neurophysiol. (2006) 95(4):2098–107. 10.1152/jn.00748.2005 16381809

[148] Abdulla FA Stebbing MJ Smith PA. Effects of substance P on excitability and ionic currents of normal and axotomized rat dorsal root ganglion neurons. Eur J Neurosci. (2001) 13:545–52. 10.1046/j.0953-816x.2000.01429.x 11168562

[149] Gudes S Barkai O Caspi Y Katz B Lev S Binshtok AM. The role of slow and persistent TTX-resistant sodium currents in acute tumor necrosis factor-alpha-mediated increase in nociceptors excitability. J Neurophysiol. (2015) 113(2):601–19. 10.1152/jn.00652.2014 25355965 PMC4297796

[150] del Rivero T Fischer R Yang F Swanson KA Bethea JR. Tumor necrosis factor receptor 1 inhibition is therapeutic for neuropathic pain in males but not in females. Pain. (2019) 160(4):922–31. 10.1097/j.pain.0000000000001470 30586024

[151] Gruber-Schoffnegger D Drdla-Schutting R Honigsperger C Wunderbaldinger G Gassner M Sandkuhler J. Induction of thermal hyperalgesia and synaptic long-term potentiation in the spinal cord Lamina I by TNF-alpha and IL-1beta is mediated by glial cells. J Neurosci. (2013) 33(15):6540–51. 10.1523/JNEUROSCI.5087-12.2013 23575851 PMC6619063

[152] Kanda H Kobayashi K Yamanaka H Okubo M Noguchi K. Microglial TNFalpha induces COX2 and PGI2 synthase expression in spinal endothelial cells during neuropathic pain. eNeuro. (2017) 4(2):ENEURO.0064–17.2017. 10.1523/ENEURO.0064-17.2017 28451639 PMC5399753

[153] Liu S Liu YP Huang ZJ Zhang YK Song AA Ma PC Wnt/Ryk signaling contributes to neuropathic pain by regulating sensory neuron excitability and spinal synaptic plasticity in rats. Pain. (2015) 156(12):2572–84. 10.1097/j.pain.0000000000000366 26407042

[154] Yuan Y Zhao Y Shen M Wang C Dong B Xie K Spinal NLRP3 inflammasome activation mediates IL-1beta release and contributes to remifentanil-induced postoperative hyperalgesia by regulating NMDA receptor NR1 subunit phosphorylation and GLT-1 expression in rats. Mol Pain. (2022) 17448069221093016. 10.1177/17448069221093016 35322721 PMC9703502

[155] Sun X Cao L Ge JL Ge JY Yang XF Du BX The NLRP3-related inflammasome modulates pain behavior in a rat model of trigeminal neuropathic pain. Life Sci. (2021) 277:119489. 10.1016/j.lfs.2021.119489 33862118

[156] Jia M Wu C Gao F Xiang H Sun N Peng P Activation of NLRP3 inflammasome in peripheral nerve contributes to paclitaxel-induced neuropathic pain. Mol Pain. (2017) 13:1744806917719804. 10.1177/1744806917719804 28714351 PMC5562344

[157] Lee MS Kim YJ. Pattern-recognition receptor signaling initiated from extracellular, membrane, and cytoplasmic space. Mol Cells. (2007) 23(1):1–10. 17464205

[158] Albiger B Dahlberg S Henriques-Normark B Normark S. Role of the innate immune system in host defence against bacterial infections: focus on the toll-like receptors. J Intern Med. (2007) 261(6):511–28. 10.1111/j.1365-2796.2007.01821.x 17547708

[159] Grace PM Strand KA Galer EL Rice KC Maier SF Watkins LR. Protraction of neuropathic pain by morphine is mediated by spinal damage associated molecular patterns (DAMPs) in male rats. Brain Behav Immun. (2018) 72:45–50. 10.1016/j.bbi.2017.08.018 28860068 PMC5832500

[160] Heilig R Dick MS Sborgi L Meunier E Hiller S Broz P. The gasdermin-D pore acts as a conduit for IL-1beta secretion in mice. Eur J Immunol. (2018) 48(4):584–92. 10.1002/eji.201747404 29274245

[161] Kawasaki Y Xu ZZ Wang X Park JY Zhuang ZY Tan PH Distinct roles of matrix metalloproteases in the early- and late-phase development of neuropathic pain. Nat Med. (2008) 14(3):331–6. 10.1038/nm1723 18264108 PMC2279180

[162] Evavold CL Ruan J Tan Y Xia S Wu H Kagan JC. The pore-forming protein gasdermin D regulates interleukin-1 secretion from living macrophages. Immunity. (2018) 48(1):35–44. 10.1016/j.immuni.2017.11.013 29195811 PMC5773350

[163] Orning P Lien E Fitzgerald KA. Gasdermins and their role in immunity and inflammation. J Exp Med. (2019) 216(11):2453–65. 10.1084/jem.20190545 31548300 PMC6829603

[164] Mousseau M Burma NE Lee KY Leduc-Pessah H Kwok CHT Reid AR Microglial pannexin-1 channel activation is a spinal determinant of joint pain. Sci Adv. (2018) 4(8):eaas9846. 10.1126/sciadv.aas9846 30101191 PMC6082646

[165] Sommer C Schmidt C George A Toyka KV. A metalloprotease-inhibitor reduces pain associated behavior in mice with experimental neuropathy. Neurosci Lett. (1997) 237(1):45–8. 10.1016/S0304-3940(97)00813-6 9406876

[166] Kucharczyk M Kurek A Detka J Slusarczyk J Papp M Tota K Chronic mild stress influences nerve growth factor through a matrix metalloproteinase-dependent mechanism. Psychoneuroendocrinology. (2016) 66:11–21. 10.1016/j.psyneuen.2015.12.019 26771945

[167] DiSabato DJ Quan N Godbout JP. Neuroinflammation: the devil is in the details. J Neurochem. (2016) 139(S2):136–53. 10.1111/jnc.13607 26990767 PMC5025335

[168] Milatovic D Zaja-Milatovic S Breyer RM Aschner M Montine TJ. Chapter 55 - neuroinflammation and oxidative injury in developmental neurotoxicity. In: Gupta RC, editor. Reproductive and developmental toxicology. 2nd edn. Cambridge, Massachusetts: Academic Press (2017). p. 1051–61.

[169] Saab CY Waxman SG Hains BC. Alarm or curse? The pain of neuroinflammation. Brain Res Rev. (2008) 58(1):226–35. 10.1016/j.brainresrev.2008.04.002 18486228

[170] Smith PA. The known biology of neuropathic pain and its relevance to pain management. Can J Neurol Sci. (2023) 1–8. [Epub ahead of print] 10.1017/cjn.2023.10 36799022

[171] Bogacka J Ciapala K Pawlik K Kwiatkowski K Dobrogowski J Przeklasa-Muszynska A CCR4 Antagonist (C021) administration diminishes hypersensitivity and enhances the analgesic potency of morphine and buprenorphine in a mouse model of neuropathic pain. Front Immunol. (2020) 11:1241. 10.3389/fimmu.2020.01241 32760393 PMC7372009

[172] Bogacka J Ciapala K Pawlik K Dobrogowski J Przeklasa-Muszynska A Mika J. Blockade of CCR4 diminishes hypersensitivity and enhances opioid analgesia - evidence from a mouse model of diabetic neuropathy. Neuroscience. (2020) 441:77–92. 10.1016/j.neuroscience.2020.06.025 32592824

[173] Schafers M Lee DH Brors D Yaksh TL Sorkin LS. Increased sensitivity of injured and adjacent uninjured rat primary sensory neurons to exogenous tumor necrosis factor-alpha after spinal nerve ligation. J Neurosci. (2003) 23(7):3028–38. 10.1523/JNEUROSCI.23-07-03028.2003 12684490 PMC6742101

[174] Zhang X Xiao HS. Gene array analysis to determine the components of neuropathic pain signaling. Curr Opin Mol Ther. (2005) 7(6):532–7. 16370375

[175] Biber K Boddeke E. Neuronal CC chemokines: the distinct roles of CCL21 and CCL2 in neuropathic pain. Front Cell Neurosci. (2014) 8:210. 10.3389/fncel.2014.00210 25147499 PMC4124792

[176] Baskozos G Dawes JM Austin JS Antunes-Martins A McDermott L Clark AJ Comprehensive analysis of long noncoding RNA expression in dorsal root ganglion reveals cell-type specificity and dysregulation after nerve injury. Pain. (2019) 160(2):463–85. 10.1097/j.pain.0000000000001416 30335683 PMC6343954

[177] Favereaux A Thoumine O Bouali-Benazzouz R Roques V Papon MA Salam SA Bidirectional integrative regulation of Cav1.2 calcium channel by microRNA miR-103: role in pain. EMBO J. (2011) 30(18):3830–41. 10.1038/emboj.2011.249 21804529 PMC3173784

[178] Su S Shao J Zhao Q Ren X Cai W Li L MiR-30b attenuates neuropathic pain by regulating voltage-gated sodium channel Nav1.3 in rats. Front Mol Neurosci. (2017) 10:126. 10.3389/fnmol.2017.00126 28529474 PMC5418349

[179] Ye G Zhang Y Zhao J Chen Y Kong L Sheng C Mir-384-5p ameliorates neuropathic pain by targeting SCN3A in a rat model of chronic constriction injury. Neurol Res. (2020) 42(4):299–307. 10.1080/01616412.2020.1723313 32098588

[180] Sakai A Saitow F Miyake N Miyake K Shimada T Suzuki H. miR-7a alleviates the maintenance of neuropathic pain through regulation of neuronal excitability. Brain. (2013) 136(Pt 9):2738–50. 10.1093/brain/awt191 23861446

[181] Sakai A Suzuki H. Emerging roles of microRNAs in chronic pain. Neurochem Int. (2014) 77:58–67. 10.1016/j.neuint.2014.05.010 24905749

[182] Sakai A Saitow F Maruyama M Miyake N Miyake K Shimada T MicroRNA cluster miR-17-92 regulates multiple functionally related voltage-gated potassium channels in chronic neuropathic pain. Nat Commun. (2017) 8:16079. 10.1093/brain/awt191 28677679 PMC5504285

[183] Malcangio M. Role of the immune system in neuropathic pain. Scand J Pain. (2020) 20(1):33–7. 10.1515/sjpain-2019-0138 31730538

[184] Gada Y Pandey A Jadhav N Ajgaonkar S Mehta D Nair S. New vistas in microRNA regulatory interactome in neuropathic pain. Front Pharmacol. (2021) 12:778014. 10.3389/fphar.2021.778014 35280258 PMC8914318

[185] Chen HP Zhou W Kang LM Yan H Zhang L Xu BH Intrathecal miR-96 inhibits Nav1.3 expression and alleviates neuropathic pain in rat following chronic construction injury. Neurochem Res. (2014) 39(1):76–83. 10.1007/s11064-013-1192-z 24234845

[186] Qiu S Liu B Mo Y Wang X Zhong L Han X MiR-101 promotes pain hypersensitivity in rats with chronic constriction injury via the MKP-1 mediated MAPK pathway. J Cell Mol Med. (2020) 24(16):8986–97. 10.1111/jcmm.15532 32656992 PMC7417728

[187] Liu L Xu D Wang T Zhang Y Yang X Wang X Epigenetic reduction of miR-214-3p upregulates astrocytic colony-stimulating factor-1 and contributes to neuropathic pain induced by nerve injury. Pain. (2020) 161(1):96–108. 10.1097/j.pain.0000000000001681 31453981

[188] Zhao YY Wu ZJ Zhu LJ Niu TX Liu B Li J. Emerging roles of miRNAs in neuropathic pain: from new findings to novel mechanisms. Front Mol Neurosci. (2023) 16:1110975. 10.3389/fnmol.2023.1110975 36873108 PMC9981676

[189] Kalpachidou T Kummer KK Kress M. Non-coding RNAs in neuropathic pain. Neuronal Signal. (2020) 4(1):NS20190099. 10.1042/NS20190099 32587755 PMC7306520

[190] Ikuma Y Sakai A Sakamoto A Suzuki H. Increased extracellular release of microRNAs from dorsal root ganglion cells in a rat model of neuropathic pain caused by peripheral nerve injury. PLoS One. (2023) 18(1):e0280425. 10.1371/journal.pone.0280425 36662897 PMC9858844

[191] Baba H Doubell TP Woolf CJ. Peripheral inflammation facilitates aβ fiber-mediated synaptic input to the substantia gelatinosa of the adult rat spinal cord. J Neurosci. (1999) 19:859–67. 10.1523/JNEUROSCI.19-02-00859.1999 9880605 PMC6782212

[192] Xanthos DN Pungel I Wunderbaldinger G Sandkuhler J. Effects of peripheral inflammation on the blood-spinal cord barrier. Mol Pain. (2012) 8:44. 10.1186/1744-8069-8-44 22713725 PMC3407004

[193] Lim TKY Shi XQ Martin HC Huang H Luheshi G Rivest S Blood-nerve barrier dysfunction contributes to the generation of neuropathic pain and allows targeting of injured nerves for pain relief. Pain. (2014) 155(5):954–67. 10.1016/j.pain.2014.01.026 24502843

[194] DeLeo JA Sorkin LS Watkins LR. Immune and glial regulation of pain. Seattle: IASP Press (2007).

[195] Greenhalgh AD David S Bennett FC. Immune cell regulation of glia during CNS injury and disease. Nat Rev Neurosci. (2020) 21(3):139–52. 10.1038/s41583-020-0263-9 32042145

[196] Hefti F. Pharmacology of nerve growth factor and discovery of tanezumab, an anti-nerve growth factor antibody and pain therapeutic. Pharmacol Res. (2020) 154:104240. 10.1016/j.phrs.2019.04.024 31026504

[197] North RY Odem MA Li Y Tatsui CE Cassidy RM Dougherty PM Electrophysiological alterations driving pain-associated spontaneous activity in human sensory neuron somata parallel alterations described in spontaneously active rodent nociceptors. J Pain. (2022) 23(8):1343–57. 10.1016/j.jpain.2022.02.009 35292377 PMC9357108

[198] Haroutounian S Nikolajsen L Bendtsen TF Finnerup NB Kristensen AD Hasselstrom JB Primary afferent input critical for maintaining spontaneous pain in peripheral neuropathy. Pain. (2014) 155(7):1272–9. 10.1016/j.pain.2014.03.022 24704366

[199] Pitcher GM Henry JL. Governing role of primary afferent drive in increased excitation of spinal nociceptive neurons in a model of sciatic neuropathy. Exp Neurol. (2008) 214(2):219–28. 10.1016/j.expneurol.2008.08.003 18773893 PMC5132624

[200] Devor M. Centralization, central sensitization and neuropathic pain. Focus on “sciatic chronic constriction injury produces cell-type-specific changes in the electrophysiological properties of rat substantia gelatinosa neurons”. J Neurophysiol. (2006) 96(2):522–3. 10.1152/jn.00365.2006 16835360

[201] Devor M Vaso A Adahan HM Vyshka G. PNS Origin of phantom limb sensation and pain: reply to letter to the editor regarding foell et al., peripheral origin of phantom limb pain: is it all resolved? Pain (2014) 155(10):2207–8. 10.1016/j.pain.2014.08.018 25168666

[202] Koplovitch P Devor M. Dilute lidocaine suppresses ectopic neuropathic discharge in dorsal root ganglia without blocking axonal propagation: a new approach to selective pain control. Pain. (2018) 159(7):1244–56. 10.1097/j.pain.0000000000001205 29533387

[203] Liu CN Raber P Ziv-Sefer S Devor M. Hyperexcitability in sensory neurons of rats selected for high versus low neuropathic pain phenotype. Neuroscience. (2001) 105(1):265–75. 10.1016/S0306-4522(01)00161-0 11483317

[204] Sukhotinsky I Ben Dor E Raber P Devor M. Key role of the dorsal root ganglion in neuropathic tactile hypersensibility. Eur J Pain. (2004) 8(2):135–43. 10.1016/S1090-3801(03)00086-7 14987623

[205] Yatziv SL Devor M. Suppression of neuropathic pain by selective silencing of dorsal root ganglion ectopia using nonblocking concentrations of lidocaine. Pain. (2019) 160(9):2105–14. 10.1097/j.pain.0000000000001602 31095098

[206] Chen C Sun L Adler A Zhou H Zhang L Zhang L Synchronized activity of sensory neurons initiates cortical synchrony in a model of neuropathic pain. Nat Commun. (2023) 14(1):689. 10.1038/s41467-023-36093-z 36755026 PMC9908980

[207] Daou I Beaudry H Ase AR Wieskopf JS Ribeiro-da-Silva A Mogil JS Optogenetic silencing of Nav1.8-positive afferents alleviates inflammatory and neuropathic pain. eNeuro. (2016) 3(1):ENEURO.0140–15.2016. 10.1523/ENEURO.0140-15.2016 27022626 PMC4794527

[208] Alles SRA Smith PA. Peripheral voltage-gated cation channels in neuropathic pain and their potential as therapeutic targets. Front Pain Res. (2021) 2:750583. 10.3389/pain.2021.750583 PMC8915663 35295464

[209] Waxman SG Zamponi GW. Regulating excitability of peripheral afferents: emerging ion channel targets. Nat Neurosci. (2014) 17(2):153–63. 10.1038/nn.3602 24473263

[210] Smith PA. K^+^ channels in primary afferents and their role in pain produced by peripheral nerev injury. Front Cell Neurosci. (2020) 14:294. 10.3389/fncel.2020.566418 PMC7528628 33093824

[211] Basso L Altier C. Transient receptor potential channels in neuropathic pain. Curr Opin Pharmacol. (2017) 32:9–15. 10.1016/j.coph.2016.10.002 27835802

[212] Du X Gamper N. Potassium channels in peripheral pain pathways: expression, function and therapeutic potential. Curr Neuropharmacol. (2013) 11(6):621–40. 10.2174/1570159X113119990042 24396338 PMC3849788

[213] Chaplan SR Guo HQ Lee DH Luo L Liu C Kuei C Neuronal hyperpolarization-activated pacemaker channels drive neuropathic pain. J Neurosci. (2003) 23(4):1169–78. 10.1523/JNEUROSCI.23-04-01169.2003 12598605 PMC6742242

[214] Amir R Michaelis M Devor M. Burst discharge in primary sensory neurons: triggered by subthreshold oscillations, maintained by depolarizing afterpotentials. J Neurosci. (2002) 22(3):1187–98. 10.1523/JNEUROSCI.22-03-01187.2002 11826148 PMC6758504

[215] Bennett GJ Doyle T Salvemini D. Mitotoxicity in distal symmetrical sensory peripheral neuropathies. Nat Rev Neurol. (2014) 10(6):326–36. 10.1038/nrneurol.2014.77 24840972 PMC4870000

[216] Stemkowsi PL Bukhanova-Schulz N Baldwin T de Chaves E Posse Smith P.A. Are sensory neurons exquisitely sensitive to interleukin 1β? J Neuroimmunol. (2021) 354:577529. 10.1016/j.jneuroim.2021.577529 33676084

[217] Sorkin LS Xiao WH Wagner R Myers RR. Tumour necrosis factor-alpha induces ectopic activity in nociceptive primary afferent fibres. Neuroscience. (1997) 81(1):255–62. 10.1016/S0306-4522(97)00147-4 9300418

[218] Jin X Gereau RW IV. Acute p38-mediated modulation of tetrodotoxin-resistant sodium channels in mouse sensory neurons by tumor necrosis factor-{alpha}. J Neurosci. (2006) 26(1):246–55. 10.1523/JNEUROSCI.3858-05.2006 16399694 PMC6674296

[219] de Macedo FHP Aires RD Fonseca EG Ferreira RCM Machado DPD Chen L TNF-alpha mediated upregulation of NaV1.7 currents in rat dorsal root ganglion neurons is independent of CRMP2 SUMOylation. Mol Brain. (2019) 12(1):117. 10.1186/s13041-019-0538-0 31888677 PMC6937926

[220] White FA Feldman P Miller RJ. Chemokine signaling and the management of neuropathic pain. Mol Interv. (2009) 9(4):188–95. 10.1124/mi.9.4.7 19720751 PMC2861804

[221] Oh SB Tran PB Gillard SE Hurley RW Hammond DL Miller RJ. Chemokines and glycoprotein120 produce pain hypersensitivity by directly exciting primary nociceptive neurons. J Neurosci. (2001) 21(14):5027–35. 10.1523/JNEUROSCI.21-14-05027.2001 11438578 PMC6762869

[222] Sun JH Yang B Donnelly DF Ma C LaMotte RH. MCP-1 enhances excitability of nociceptive neurons in chronically compressed dorsal root ganglia. J Neurophysiol. (2006) 96(5):2189–99. 10.1152/jn.00222.2006 16775210

[223] White FA Sun J Waters SM Ma C Ren D Ripsch M Excitatory monocyte chemoattractant protein-1 signaling is up-regulated in sensory neurons after chronic compression of the dorsal root ganglion. Proc Natl Acad Sci U S A. (2005) 102(39):14092–7. 10.1073/pnas.0503496102 16174730 PMC1236537

[224] Jung H Toth PT White FA Miller RJ. Monocyte chemoattractant protein-1 functions as a neuromodulator in dorsal root ganglia neurons. J Neurochem. (2008) 104(1):254–63. 10.1111/j.1471-4159.2007.04969.x 17944871 PMC2186066

[225] Gold MS Reichling DB Shuster MJ Levine JD. Hyperalgesic agents increase a tetrodotoxin-resistant na+ current in nociceptors. Proc Natl Acad Sci U S A. (1996) 93(3):1108–12. 10.1073/pnas.93.3.1108 8577723 PMC40039

[226] Zamponi GW Striessnig J Koschak A Dolphin AC. The Physiology, Pathology, And pharmacology of voltage-gated calcium channels and their future therapeutic potential. Pharmacol Rev. (2015) 67(4):821–70. 10.1124/pr.114.009654 26362469 PMC4630564

[227] Du X Gao H Jaffe D Zhang H Gamper N. M-type K(+) channels in peripheral nociceptive pathways. Br J Pharmacol. (2018) 175(12):2158–72. 10.1111/bph.13978 28800673 PMC5980636

[228] Jones F Gamper N Gao H. Kv7 channels and excitability disorders. Handb Exp Pharmacol. (2021) 267:185–230. 10.1007/164_2021_457 33860384

[229] Laumet G Garriga J Chen SR Zhang Y Li DP Smith TM G9a is essential for epigenetic silencing of K^+^ channel genes in acute-to-chronic pain transition. Nat Neurosci. (2015) 18(12):1746–55. 10.1038/nn.4165 26551542 PMC4661086

[230] Liang L Zhao JY Kathryn T Bekker A Tao YX. BIX01294, A G9a inhibitor, alleviates nerve injury-induced pain hypersensitivities during both development and maintenance periods. Transl Perioper Pain Med. (2019) 6(4):106–14. 10.31480/2330-4871/097 31497620 PMC6730651

[231] Wang X Shen X Ma S Liu Y Xu S Wu S Threshold effect of G9a/glp on peripheral nerve injury induced hypersensitivity. Mol Pain. (2017) 13:1744806917729305. 10.1177/1744806917729305 28814147 PMC5588794

[232] Rugo HS Jacobs I Sharma S Scappaticci F Paul TA Jensen-Pergakes K The promise for histone methyltransferase inhibitors for epigenetic therapy in clinical oncology: a narrative review. Adv Ther. (2020) 37(7):3059–82. 10.1007/s12325-020-01379-x 32445185 PMC7467409

[233] Dib-Hajj SD Yang Y Black JA Waxman SG. The na(V)1.7 sodium channel: from molecule to man. Nat Rev Neurosci. (2013) 14(1):49–62. 10.1038/nrn3404 23232607

[234] Dib-Hajj SD Black JA Waxman SG. Nav1.9: a sodium channel linked to human pain. Nat Rev Neurosci. (2015) 16(9):511–9. 10.1038/nrn3977 26243570

[235] Kotecha M Cheshire WP Finnigan H Giblin K Naik H Palmer J Design of phase 3 studies evaluating vixotrigine for treatment of trigeminal neuralgia. J Pain Res. (2020) 13:1601–9. 10.2147/JPR.S247182 32669869 PMC7335847

[236] Zakrzewska JM Palmer J Morisset V Giblin GM Obermann M Ettlin DA Safety and efficacy of a Nav1.7 selective sodium channel blocker in patients with trigeminal neuralgia: a double-blind, placebo-controlled, randomised withdrawal phase 2a trial. Lancet Neurol. (2017) 16(4):291–300. 10.1016/S1474-4422(17)30005-4 28216232

[237] Dib-Hajj SD Black JA Cummins TR Kenney AM Kocsis JD Waxman SG. Rescue of alpha-SNS sodium channel expression in small dorsal root ganglion neurons after axotomy by nerve growth factor in vivo. J Neurophysiol. (1998) 79(5):2668–76. 10.1152/jn.1998.79.5.2668 9582237

[238] Patel MK Kaye AD Urman RD. Tanezumab: therapy targeting nerve growth factor in pain pathogenesis. J Anaesthesiol Clin Pharmacol. (2018) 34(1):111–6. 10.4103/joacp.JOACP_389_15 29643634 PMC5885425

[239] Bagal SK Andrews M Bechle BM Bian J Bilsland J Blakemore DC Discovery of potent, selective, and peripherally restricted pan-trk kinase inhibitors for the treatment of pain. J Med Chem. (2018) 61(15):6779–800. 10.1021/acs.jmedchem.8b00633 29944371

[240] Bagal SK Omoto K Blakemore DC Bungay PJ Bilsland JG Clarke PJ Discovery of Allosteric, Potent, Subtype Selective, And peripherally restricted TrkA kinase inhibitors. J Med Chem. (2019) 62(1):247–65. 10.1021/acs.jmedchem.8b00280 29672039

[241] Tang S Xue Y Dengqi X Shao L. Design, development and evaluation of a prodrug-type TrkA-selective inhibitor with antinociceptive effects in vivo. Eur J Med Chem. (2023) 245(Pt 2):114901. 10.1016/j.ejmech.2022.114901 36423414

[242] Ramachandra R Hassan B McGrew SG Dompor J Farrag M Ruiz-Velasco V Identification of CaV channel types expressed in muscle afferent neurons. J Neurophysiol. (2013) 110(7):1535–43. 10.1152/jn.00069.2013 23843437 PMC4042423

[243] Rose KE Lunardi N Boscolo A Dong X Erisir A Jevtovic-Todorovic V Immunohistological demonstration of CaV3.2 T-type voltage-gated calcium channel expression in soma of dorsal root ganglion neurons and peripheral axons of rat and mouse. Neuroscience. (2013) 250:263–74. 10.1016/j.neuroscience.2013.07.005 23867767 PMC3796369

[244] Talley EM Cribbs LL Lee JH Daud A Perez-Reyes E Bayliss DA. Differential distribution of three members of a gene family encoding low voltage-activated (T-type) calcium channels. J Neurosci. (1999) 19(6):1895–911. 10.1523/JNEUROSCI.19-06-01895.1999 10066243 PMC6782581

[245] Altier C Zamponi GW. Targeting Ca2+ channels to treat pain: t-type versus N-type. Trends Pharmacol Sci. (2004) 25(9):465–70. 10.1016/j.tips.2004.07.004 15559248

[246] Rettig J Sheng ZH Kim DK Hodson CD Snutch TP Catterall WA. Isoform-specific interaction of the alpha1A subunits of brain Ca2+ channels with the presynaptic proteins syntaxin and SNAP-25. Proc Natl Acad Sci U S A. (1996) 93(14):7363–8. 10.1073/pnas.93.14.7363 8692999 PMC38990

[247] Yang J Xie MX Hu L Wang XF Mai JZ Li YY Upregulation of N-type calcium channels in the soma of uninjured dorsal root ganglion neurons contributes to neuropathic pain by increasing neuronal excitability following peripheral nerve injury. Brain Behav Immun. (2018) 71:52–65. 10.1016/j.bbi.2018.04.016 29709527

[248] Alles SR Garcia E Balasubramanyan S Jones K Tyson JR Joy T Peripheral nerve injury increases contribution of L-type calcium channels to synaptic transmission in spinal lamina II: role of alpha2delta-1 subunits. Mol Pain. (2018) 14:1744806918765806. 10.1177/1744806918765806 29580153 PMC5882044

[249] Jagodic MM Pathirathna S Joksovic PM Lee W Nelson MT Naik AK Upregulation of the T-type calcium current in small rat sensory neurons after chronic constrictive injury of the sciatic nerve. J Neurophysiol. (2008) 99(6):3151–6. 10.1152/jn.01031.2007 18417624 PMC2667888

[250] Bourinet E Altier C Hildebrand ME Trang T Salter MW Zamponi GW. Calcium-permeable ion channels in pain signaling. Physiol Rev. (2014) 94(1):81–140. 10.1152/physrev.00023.2013 24382884

[251] Patel R Montagut-Bordas C Dickenson AH. Calcium channel modulation as a target in chronic pain control. Br J Pharmacol. (2018) 175(12):2173–84. 10.1111/bph.13789 28320042 PMC5980588

[252] Tibbs GR Posson DJ Goldstein PA. Voltage-gated ion channels in the PNS: novel therapies for neuropathic pain? Trends Pharmacol Sci. (2016) 37(7):522–42. 10.1016/j.tips.2016.05.002 27233519

[253] Zamponi GW. Targeting voltage-gated calcium channels in neurological and psychiatric diseases. Nat Rev Drug Discov. (2016) 15(1):19–34. 10.1038/nrd.2015.5 26542451

[254] Matis G De NP Dupoiron D Likar R Zuidema X Rasche D. Intrathecal pain management with ziconotide: time for consensus? Brain Behav. (2021)11(Suppl 1):e02055. 10.1002/brb3.2055 33690987 PMC7943290

[255] Abdulla FA Smith PA. Ectopic α_2_-adrenoceptors couple to N-type Ca^2+^ channels in axotomized rat sensory neurons. J Neurosci. (1997) 17(5):1633–41. 10.1523/JNEUROSCI.17-05-01633.1997 9030623 PMC6573393

[256] Zamponi GW Snutch TP. Modulation of voltage-dependent calcium channels by G proteins. Curr Opin Neurobiol. (1998) 8(3):351–6. 10.1016/S0959-4388(98)80060-3 9687363

[257] Kirkpatrick AF Derasari M Glodek JA Piazza PA. Postherpetic neuralgia: a possible application for topical clonidine. Anesthesiology. (1992) 76(6):1065–6. 10.1097/00000542-199206000-00044 1318008

[258] Fink EA Xu J Hubner H Braz JM Seemann P Avet C Structure-based discovery of nonopioid analgesics cting through the alpha2A-adrenergic receptor. Science. (2022) 377(6614):eabn7065. 10.1126/science.abn7065 36173843 PMC10360211

[259] Greene SA Thurmon JC. Xylazine: a review of its pharmacology and use in veterinary medicine. J Vet Pharmacol Ther. (1988) 11(4):295–313. 10.1111/j.1365-2885.1988.tb00189.x 3062194

[260] Friedman J Montero F Bourgois P Wahbi R Dye D Goodman-Meza D Xylazine spreads across the US: a growing component of the increasingly synthetic and polysubstance overdose crisis. Drug Alcohol Depend. (2022) 233:109380. 10.1016/j.drugalcdep.2022.109380 35247724 PMC9128597

[261] Moore RA Wiffen PJ Derry S Toelle T Rice AS. Gabapentin for chronic neuropathic pain and fibromyalgia in adults. Cochrane Database Syst Rev. (2014) 4:CD007938. 10.1002/14651858.CD007938.pub2 PMC6464253 24771480

[262] Dolphin AC. Calcium channel auxiliary alpha2delta and beta subunits: trafficking and one step beyond. Nat Rev Neurosci. (2012) 13(8):542–55. 10.1038/nrn3311 22805911

[263] Eroglu C Allen NJ Susman MW O’Rourke NA Park CY Ozkan E Gabapentin receptor alpha2delta-1 is a neuronal thrombospondin receptor responsible for excitatory CNS synaptogenesis. Cell. (2009) 139(2):380–92. 10.1016/j.cell.2009.09.025 19818485 PMC2791798

[264] Hoppa MB Lana B Margas W Dolphin AC Ryan TA. Alpha2delta expression sets presynaptic calcium channel abundance and release probability. Nature. (2012) 486(7401):122–5. 10.1038/nature11033 22678293 PMC3376018

[265] Field MJ Cox PJ Stott E Melrose H Offord J Su TZ Identification of the {alpha}2-{delta}-1 subunit of voltage-dependent calcium channels as a molecular target for pain mediating the analgesic actions of pregabalin. PNAS. (2006) 103(46):17537–42. 10.1073/pnas.0409066103 17088553 PMC1859964

[266] Li L Chen SR Zhou MH Wang L Li DP Chen H Alpha 2 Delta 1 switches the phenotype of synaptic AMPA receptors by physically disrupting heteromeric subunit assembly. Cell Rep. (2021) 36(3):109396. 10.1016/j.celrep.2021.109396 34289359 PMC8353586

[267] Chen J Li L Chen SR Chen H Xie JD Sirrieh RE The alpha2delta-1-NMDA receptor Complex is critically involved in neuropathic pain development and gabapentin therapeutic actions. Cell Rep. (2018) 22(9):2307–21. 10.1016/j.celrep.2018.02.021 29490268 PMC5873963

[268] Abdulla FA Smith PA. Axotomy- and autotomy-induced changes in Ca^2+^ and K^+^ channel currents of rat dorsal root ganglion neurons. J Neurophysiol. (2001) 85:644–58. 10.1152/jn.2001.85.2.644 11160500

[269] Jagodic MM Pathirathna S Nelson MT Mancuso S Joksovic PM Rosenberg ER Cell-Specific alterations of T-type calcium current in painful diabetic neuropathy enhance excitability of sensory neurons. J Neurosci. (2007) 27(12):3305–16. 10.1523/JNEUROSCI.4866-06.2007 17376991 PMC6672477

[270] Francois A Schuetter N Laffray S Sanguesa J Pizzoccaro A Dubel S The low-threshold calcium channel Cav3.2 determines low-threshold mechanoreceptor function. Cell Rep. (2015) 10(3):370–82. 10.1016/j.celrep.2014.12.042 25600872

[271] Jacus MO Uebele VN Renger JJ Todorovic SM. Presynaptic CaV3.2 channels regulate excitatory neurotransmission in nociceptive dorsal horn neurons. J Neurosci. (2012) 32(27):9374–82. 10.1523/JNEUROSCI.0068-12.2012 22764245 PMC3398424

[272] Garcia-Caballero A Gadotti VM Stemkowski P Weiss N Souza IA Hodgkinson V The deubiquitinating enzyme USP5 modulates neuropathic and inflammatory pain by enhancing Cav3.2 channel activity. Neuron. (2014) 83(5):1144–58. 10.1016/j.neuron.2014.07.036 25189210

[273] Alaklabi AM Gambeta E Zamponi GW. Electrophysiological characterization of a CaV3.1 calcium channel mutation linked to trigeminal neuralgia. Pflugers Arch. (2023) 475:711–8. 10.1007/s00424-023-02808-w 37010626

[274] Choe W Messinger RB Leach E Eckle VS Obradovic A Salajegheh R TTA-P2 is a potent and selective blocker of T-type calcium channels in rat sensory neurons and a novel antinociceptive agent. Mol Pharmacol. (2011) 80(5):900–10. 10.1124/mol.111.073205 21821734 PMC3198916

[275] Francois A Kerckhove N Meleine M Alloui A Barrere C Gelot A State-dependent properties of a new T-type calcium channel blocker enhance ca(V)3.2 selectivity and support analgesic effects. Pain. (2013) 154(2):283–93. 10.1016/j.pain.2012.10.023 23257507

[276] Snutch TP Zamponi GW. Recent advances in the development of T-type calcium channel blockers for pain intervention. Br J Pharmacol. (2017) 175:2375–83. 10.1111/bph.13906 28608534 PMC5980537

[277] Tringham E Powell KL Cain SM Kuplast K Mezeyova J Weerapura M T-type calcium channel blockers that attenuate thalamic burst firing and suppress absence seizures. Sci Transl Med. (2012) 4(121):121ra19. 10.1126/scitranslmed.3003120 22344687

[278] Harding EK Dedek A Bonin RP Salter MW Snutch TP Hildebrand ME. The T-type calcium channel antagonist, Z944, reduces spinal excitability and pain hypersensitivity. Br J Pharmacol. (2021) 178:3517–32. 10.1111/bph.15498 33871884 PMC8453510

[279] Zamponi GW Lewis RJ Todorovic SM Arneric SP Snutch TP. Role of voltage-gated calcium channels in ascending pain pathways. Brain Res Rev. (2009) 60(1):84–9. 10.1016/j.brainresrev.2008.12.021 19162069 PMC2692704

[280] Nam G. T-type calcium channel blockers: a patent review (2012-2018). Null. (2018) 28(12):883–901. 10.1080/13543776.2018.1541982 30372652

[281] Hauser W Petzke F Fitzcharles MA. Efficacy, tolerability and safety of cannabis-based medicines for chronic pain management - an overview of systematic reviews. Eur J Pain. (2018) 22(3):455–70. 10.1002/ejp.1118 29034533

[282] Ross HR Napier I Connor M. Inhibition of recombinant human T-type calcium channels by Delta9-tetrahydrocannabinol and cannabidiol. J Biol Chem. (2008) 283(23):16124–34. 10.1074/jbc.M707104200 18390906 PMC3259625

[283] Bladen C Mirlohi S Santiago M Longworth M Kassiou M Banister S Modulation of human T-type calcium channels by synthetic cannabinoid receptor agonists in vitro. Neuropharmacology. (2021) 187:108478. 10.1016/j.neuropharm.2021.108478 33600843

[284] Mackie K Hille B. Cannabinoids inhibit N-type calcium channels in neuroblastoma-glioma cells. Proc Natl Acad Sci U S A. (1992) 89(9):3825–9. 10.1073/pnas.89.9.3825 1315042 PMC525583

[285] Li Y Zhang L Wu Y Zheng Q Chen M Qian Z Cannabinoids-induced peripheral analgesia depends on activation of BK channels. Brain Res. (2019) 1711:23–8. 10.1016/j.brainres.2019.01.007 30615887

[286] Zhang HXB Bean BP. Cannabidiol inhibition of murine primary nociceptors: tight binding to slow inactivated states of Nav1.8 channels. J Neurosci. (2021) 41(30):6371. 10.1523/JNEUROSCI.3216-20.2021 34131037 PMC8318087

[287] You H Altier C Zamponi GW. CCR2 Receptor ligands inhibit Cav3.2 T-type calcium channels. Mol Pharmacol. (2010) 77(2):211–7. 10.1124/mol.109.059022 19864434

[288] You H Gadotti VM Petrov RR Zamponi GW Diaz P. Functional characterization and analgesic effects of mixed cannabinoid receptor/T-type channel ligands. Mol Pain. (2011) 7:89. 10.1124/mol.109.059022 22093952 PMC3250956

[289] Rangel-Galvan M Castro ME Perez-Aguilar JM Caballero NA Rangel-Huerta A Melendez FJ. Theoretical study of the structural stability, chemical reactivity, and protein interaction for NMP compounds as modulators of the endocannabinoid system. Molecules. (2022) 27(2):414. 10.3390/molecules27020414 35056729 PMC8779749

[290] Tomita S Sekiguchi F Kasanami Y Naoe K Tsubota M Wake H Cav3.2 overexpression in L4 dorsal root ganglion neurons after L5 spinal nerve cutting involves egr-1, USP5 and HMGB1 in rats: An emerging signaling pathway for neuropathic pain. Eur J Pharmacol. (2020) 888:173587. 10.1016/j.ejphar.2020.173587 32971090

[291] Ali MY Gadotti VM Huang S Garcia-Caballero A Antunes FTT Jung HA Icariside II, a prenyl-flavonol, Alleviates Inflammatory and Neuropathic Pain by Inhibiting T-Type Calcium Channels and USP5–Cav3.2 Interactions. ACS Chem Neurosci. (2023) 14(10):1859–69. 10.1021/acschemneuro.3c00083 37116219

[292] Garcia-Caballero A Gadotti VM Ali MY Bladen C Gambeta E Van Humbeck JF A synthetically accessible small-molecule inhibitor of USP5-Cav3.2 calcium channel interactions with analgesic properties. ACS Chem Neurosci. (2022) 13(4):524–36. 10.1021/acschemneuro.1c00765 35113527

[293] Gadotti VM Caballero AG Berger ND Gladding CM Chen L Pfeifer TA Small organic molecule disruptors of Cav3.2 - USP5 interactions reverse inflammatory and neuropathic pain. Mol Pain. (2015) 11:12. 10.1186/s12990-015-0011-8 25889575 PMC4364099

[294] Garcia-Caballero A Gadotti VM Chen L Zamponi GW. A cell-permeant peptide corresponding to the cUBP domain of USP5 reverses inflammatory and neuropathic pain. Mol Pain. (2016) 12:1744806916642444. 10.1177/1744806916642444 27130589 PMC4955966

[295] Smith T Al OM Sathish J Djouhri L. Increased expression of HCN2 channel protein in L4 dorsal root ganglion neurons following axotomy of L5- and inflammation of L4-spinal nerves in rats. Neuroscience. (2015) 295:90–102. 10.1016/j.neuroscience.2015.03.041 25813712

[296] Emery EC Young GT Berrocoso EM Chen L McNaughton PA. HCN2 ion channels play a central role in inflammatory and neuropathic pain. Science. (2011) 333(6048):1462–6. 10.1126/science.1206243 21903816

[297] Djouhri L Smith T Ahmeda A Alotaibi M Weng X. Hyperpolarization-activated cyclic nucleotide-gated channels contribute to spontaneous activity in L4 C-fiber nociceptors, but not abeta-non-nociceptors, after axotomy of L5-spinal nerve in the rat in vivo. Pain. (2018) 159(7):1392–402. 10.1097/j.pain.0000000000001224 29578948

[298] Antal M Papp I Bahaerguli N Veress G Vereb G. Expression of hyperpolarization-activated and cyclic nucleotide-gated cation channel subunit 2 in axon terminals of peptidergic nociceptive primary sensory neurons in the superficial spinal dorsal horn of rats. Eur J Neurosci. (2004) 19(5):1336–42. 10.1111/j.1460-9568.2004.03235.x 15016091

[299] Papp I Szucs P Hollo K Erdelyi F Szabo G Antal M. Hyperpolarization-activated and cyclic nucleotide-gated cation channel subunit 2 ion channels modulate synaptic transmission from nociceptive primary afferents containing substance P to secondary sensory neurons in laminae I-IIo of the rodent spinal dorsal horn. Eur J Neurosci. (2006) 24(5):1341–52. 10.1111/j.1460-9568.2006.05013.x 16987220

[300] Young GT Emery EC Mooney ER Tsantoulas C McNaughton PA. Inflammatory and neuropathic pain are rapidly suppressed by peripheral block of hyperpolarisation-activated cyclic nucleotide-gated ion channels. Pain. (2014) 155(9):1708–19. 10.1016/j.pain.2014.05.021 24861581

[301] Noh S Kumar N Bukhanova N Chen Y Stemkowsi PL Smith PA. The heart-rate-reducing agent, ivabradine, reduces mechanical allodynia in a rodent model of neuropathic pain. Eur J Pain. (2014) 18:1139–47. 10.1002/j.1532-2149.2014.00460.x 24677354

[302] Santoro B Shah MM. Hyperpolarization-Activated cyclic nucleotide-gated channels as drug targets for neurological disorders. Annu Rev Pharmacol Toxicol. (2020) 60:109–31. 10.1146/annurev-pharmtox-010919-023356 31914897

[303] Tsantoulas C Mooney ER McNaughton PA. HCN2 ion channels: basic science opens up possibilities for therapeutic intervention in neuropathic pain. Biochem J. (2016) 473(18):2717–36. 10.1042/BCJ20160287 27621481

[304] Bernard Healey SA Scholtes I Abrahams M McNaughton PA Menon DK Lee MC. Role of hyperpolarization-activated cyclic nucleotide-gated ion channels in neuropathic pain: a proof-of-concept study of ivabradine in patients with chronic peripheral neuropathic pain. Pain Rep. (2021) 6(4):e967. 10.1097/PR9.0000000000000967 34712888 PMC8547924

[305] Vilceanu D Honore P Hogan QH Stucky CL. Spinal nerve ligation in mouse upregulates TRPV1 heat function in injured IB4-positive nociceptors. J Pain. (2010) 11(6):588–99. 10.1016/j.jpain.2009.09.018 20015699 PMC2879455

[306] Iftinca M Defaye M Altier C. TRPV1-Targeted Drugs in development for human pain conditions. Drugs. (2020) 81:7–27. 10.1007/s40265-020-01429-2 33165872

[307] Binshtok AM Bean BP Woolf CJ. Inhibition of nociceptors by TRPV1-mediated entry of impermeant sodium channel blockers. Nature. (2007) 449(7162):607–10. 10.1038/nature06191 17914397

[308] Landry M Holmberg K Zhang X Hokfelt T. Effect of axotomy on expression of NPY, galanin, and NPY Y1 and Y2 receptors in dorsal root ganglia and the superior cervical ganglion studied with double-labeling in situ hybridization and immunohistochemistry. Exp Neurol. (2000) 162(2):361–84. 10.1006/exnr.1999.7329 10739642

[309] Wakisaka S Kajander KC Bennett GJ. Increased neuropeptide Y (NPY)-like immunoreactivity in rat sensory neurons following peripheral axotomy. Neurosci Lett. (1991) 124:200–3. 10.1016/0304-3940(91)90093-9 1712437

[310] Noguchi K De Leon M Nahin RL Senba E Ruda MA. Quantification of axotomy-induced alteration of neuropeptide mRNAs in dorsal root ganglion neurons with special reference to neuropeptide Y mRNA and the effects of neonatal capsaicin treatment. J Neurosci Res. (1993) 35(1):54–66. 10.1002/jnr.490350108 7685398

[311] Abdulla FA Smith PA. Nerve injury increases an excitatory action of neuropeptide Y and Y2- agonists on dorsal root ganglion neurons. Neuroscience. (1999) 89(1):43–60. 10.1016/S0306-4522(98)00443-6 10051216

[312] Huang LY Neher E. Ca(2+)-dependent exocytosis in the somata of dorsal root ganglion neurons. Neuron. (1996) 17(1):135–45. 10.1016/S0896-6273(00)80287-1 8755485

[313] Eberhardt M Hoffmann T Sauer SK Messlinger K Reeh PW Fischer MJ. Calcitonin gene-related peptide release from intact isolated dorsal root and trigeminal ganglia. Neuropeptides. (2008) 42(3):311–7. 10.1016/j.npep.2008.01.002 18328558

[314] Sauer SK Reeh PW Bove GM. Noxious heat-induced CGRP release from rat sciatic nerve axons in vitro. Eur J Neurosci. (2001) 14(8):1203–8. 10.1046/j.0953-816x.2001.01741.x 11703449

[315] Gardell LR Vanderah TW Gardell SE Wang R Ossipov MH Lai J Enhanced evoked excitatory transmitter release in experimental neuropathy requires descending facilitation. J Neurosci. (2003) 23(23):8370–9. 10.1523/JNEUROSCI.23-23-08370.2003 12967999 PMC6740686

[316] Dray A Pinnock RD. Effects of substance P on adult rat sensory ganglion neurones in vitro. Neurosci Lett. (1982) 33(1):61–6. 10.1016/0304-3940(82)90130-6 6185885

[317] Natura G von Banchet GS Schaible HG. Calcitonin gene-related peptide enhances TTX-resistant sodium currents in cultured dorsal root ganglion neurons from adult rats. Pain. (2005) 116(3):194–204. 10.1016/j.pain.2005.04.002 15927395

[318] McMahon SB La Russa F. Bennett DL. Crosstalk between the nociceptive and immune systems in host defence and disease. Nat Rev Neurosci. (2015) 16(7):389–402. 10.1038/nrn3946 26087680

[319] Matsuka Y Afroz S Dalanon JC Iwasa T Waskitho A Oshima M. The role of chemical transmitters in neuron-glia interaction and pain in sensory ganglion. Neurosci Biobehav Rev. (2020) 108:393–9. 10.1016/j.neubiorev.2019.11.019 31785264

[320] Goldstein DJ Wang O Gitter BD Iyengar S. Dose-response study of the analgesic effect of lanepitant in patients with painful diabetic neuropathy. Clin Neuropharmacol. (2001) 24(1):16–22. 10.1097/00002826-200101000-00004 11290877

[321] Parascandolo E Levinson K Rizzoli P Sharon R. Efficacy of erenumab in the treatment of trigeminal neuralgia: a retrospective case series. Neurol Clin Pract. (2021) 11(3):227–31. 10.1212/CPJ.0000000000001075 34484889 PMC8382339

[322] Schott Andersen AS Maarbjerg S Noory N Heinskou TB Forman JL Cruccu G Safety and efficacy of erenumab in patients with trigeminal neuralgia in Denmark: a double-blind, randomised, placebo-controlled, proof-of-concept study. Lancet Neurol. (2022) 21(11):994–1003. 10.1016/S1474-4422(22)00294-0 36113495

[323] Paige C Plasencia-Fernandez I Kume M Papalampropoulou-Tsiridou M Lorenzo LE David ET A female-specific role for calcitonin gene-related peptide (CGRP) in rodent pain models. J Neurosci. (2022) 42(10):1930–44. 10.1523/JNEUROSCI.1137-21.2022 35058371 PMC8916765

[324] Ahlstrom FHG Matlik K Viisanen H Blomqvist KJ Liu X Lilius TO Spared nerve injury causes sexually dimorphic mechanical allodynia and differential gene expression in spinal cords and dorsal root ganglia in rats. Mol Neurobiol. (2021) 58(10):5396–419. 10.1007/s12035-021-02447-1 34331199 PMC8497331

[325] Chiu IM von Hehn CA Woolf CJ. Neurogenic inflammation and the peripheral nervous system in host defense and immunopathology. Nat Neurosci. (2012) 15(8):1063–7. 10.1038/nn.3144 22837035 PMC3520068

[326] Talbot S Foster SL Woolf CJ. Neuroimmunity: physiology and pathology. Annu Rev Immunol. (2016) 34:421–47. 10.1146/annurev-immunol-041015-055340 26907213

[327] Tracey KJ. Reflex control of immunity. Nat Rev Immunol. (2009) 9(6):418–28. 10.1038/nri2566 19461672 PMC4535331

[328] Chavan SS Pavlov VA Tracey KJ. Mechanisms and therapeutic relevance of neuro-immune communication. Immunity. (2017) 46(6):927–42. 10.1016/j.immuni.2017.06.008 28636960 PMC5578398

[329] Xanthos DN Sandkuhler J. Neurogenic neuroinflammation: inflammatory CNS reactions in response to neuronal activity. Nat Rev Neurosci. (2014) 15(1):43–53. 10.1038/nrn3617 24281245

[330] An Q Sun C Li R Chen S Gu X An S Calcitonin gene-related peptide regulates spinal microglial activation through the histone H3 lysine 27 trimethylation via enhancer of zeste homolog-2 in rats with neuropathic pain. J Neuroinflammation. (2021) 18(1):117. 10.1186/s12974-021-02168-1 34020664 PMC8139106

[331] Shi X Wang L Li X Sahbaie P Kingery WS Clark JD. Neuropeptides contribute to peripheral nociceptive sensitization by regulating interleukin-1beta production in keratinocytes. Anesth Analg. (2011) 113(1):175–83. 10.1213/ANE.0b013e31821a0258 21596883 PMC3123433

[332] Kremer M Yalcin I Goumon Y Wurtz X Nexon L Daniel D A dual noradrenergic mechanism for the relief of neuropathic allodynia by the antidepressant drugs duloxetine and amitriptyline. J Neurosci. (2018) 38(46):9934–54. 10.1523/JNEUROSCI.1004-18.2018 30249798 PMC6596240

[333] Damo E Agarwal A Simonetti M. Activation of beta2-adrenergic receptors in microglia alleviates neuropathic hypersensitivity in mice. Cells. (2023) 12(2):284. 10.3390/cells12020284 36672219 PMC9856373

[334] Bailey AL Ribeiro-da-Silva A. Transient loss of terminals from non-peptidergic nociceptive fibers in the substantia gelatinosa of spinal cord following chronic constriction injury of the sciatic nerve. Neuroscience. (2006) 138(2):675–90. 10.1016/j.neuroscience.2005.11.051 16413131

[335] Yousefpour N Locke S Deamond H Wang C Marques L St-Louis M Time-dependent and selective microglia-mediated removal of spinal synapses in neuropathic pain. Cell Rep. (2023) 42(1):112010. 10.1016/j.celrep.2023.112010 36656715

[336] Cheng CF Cheng JK Chen CY Rau RH Chang YC Tsaur ML. Nerve growth factor-induced synapse-like structures in contralateral sensory ganglia contribute to chronic mirror-image pain. Pain. (2015) 156(11):2295–309. 10.1097/j.pain.0000000000000280 26121254

[337] Smithson LJ Krol KM Kawaja MD. Neuronal degeneration associated with sympathosensory plexuses in the trigeminal ganglia of aged mice that overexpress nerve growth factor. Neurobiol Aging. (2014) 35(12):2812–21. 10.1016/j.neurobiolaging.2014.06.014 25037287

[338] Xie W Strong JA Li H Zhang JM. Sympathetic sprouting near sensory neurons after nerve injury occurs preferentially on spontaneously active cells and is reduced by early nerve block. J Neurophysiol. (2007) 97(1):492–502. 10.1152/jn.00899.2006 17065247 PMC1774587

[339] Yen LD Bennett GJ Ribeiro-da-Silva A. Sympathetic sprouting and changes in nociceptive sensory innervation in the glabrous skin of the rat hind paw following partial peripheral nerve injury. J Comp Neurol. (2006) 495(6):679–90. 10.1002/cne.20899 16506190

[340] Ramer MS Bisby MA. Sympathetic axons surround neuropeptide-negative axotomized sensory neurons. Neuroreport. (1998) 9(13):3109–13. 10.1097/00001756-199809140-00035 9804325

[341] Devor M Janig W Michaelis M. Modulation of activity in dorsal root ganglion neurons by sympathetic activation in nerve-injured rats. J Neurophysiol. (1994) 71:38–47. 10.1152/jn.1994.71.1.38 8158237

[342] Pertin M Allchorne AJ Beggah AT Woolf CJ Decosterd I. Delayed sympathetic dependence in the spared nerve injury (SNI) model of neuropathic pain. Mol Pain. (2007) 3:21. 10.1186/1744-8069-3-21 17672895 PMC1950869

[343] Rho RH Brewer RP Lamer TJ Wilson PR. Complex regional pain syndrome. Mayo Clin Proc. (2002) 77(2):174–80. 10.1016/S0025-6196(11)62332-X 11838651

[344] Ji RR Xu ZZ Strichartz G Serhan CN. Emerging roles of resolvins in the resolution of inflammation and pain. Trends Neurosci. (2011) 34(11):599–609. 10.1016/j.tins.2011.08.005 21963090 PMC3200462

[345] Ji RR. Specialized pro-resolving mediators as resolution pharmacology for the control of pain and itch. Annu Rev Pharmacol Toxicol. (2022) 63:273–93. 10.1146/annurev-pharmtox-051921-084047 36100219 PMC10290889

[346] Buckley CD Gilroy DW Serhan CN. Proresolving lipid mediators and mechanisms in the resolution of acute inflammation. Immunity. (2014) 40(3):315–27. 10.1016/j.immuni.2014.02.009 24656045 PMC4004957

[347] Buckley CD Gilroy DW Serhan CN Stockinger B Tak PP. The resolution of inflammation. Nat Rev Immunol. (2013) 13(1):59–66. 10.1038/nri3362 23197111

[348] Sankaranarayanan I Tavares-Ferreira D Mwirigi JM Mejia GL Burton MD Price TJ. Inducible co-stimulatory molecule (ICOS) alleviates paclitaxel-induced neuropathic pain via an IL-10-mediated mechanism in female mice. J Neuroinflammation. (2023) 20(1):32. 10.1186/s12974-023-02719-8 36774519 PMC9922469

[349] Flatters SJ Fox AJ Dickenson AH. Nerve injury alters the effects of interleukin-6 on nociceptive transmission in peripheral afferents. Eur J Pharmacol. (2004) 484(2–3):183–91. 10.1016/j.ejphar.2003.11.013 14744602

[350] Flatters SJ Fox AJ Dickenson AH. Spinal interleukin-6 (IL-6) inhibits nociceptive transmission following neuropathy. Brain Res. (2003) 984(1–2):54–62. 10.1016/S0006-8993(03)03092-0 12932839

[351] Chalaki M Cruz LJ van Neerven SGA Verhaagen J Dahan A Malessy MJA. Molecular changes in the dorsal root ganglion during the late phase of peripheral nerve injury-induced pain in rodents: a systematic review. Anesthesiology. (2022) 136(2):362–88. 10.1097/ALN.0000000000004092 34965284

[352] Ray PR Shiers S Caruso JP Tavares-Ferreira D Sankaranarayanan I Uhelski ML RNA profiling of human dorsal root ganglia reveals sex-differences in mechanisms promoting neuropathic pain. Brain. (2023) 146(2):749–66. 10.1093/brain/awac266 35867896 PMC10169414

[353] Yu X Basbaum A Guan Z. Contribution of colony-stimulating factor 1 to neuropathic pain. Pain Rep. (2021) 6(1):e883. 10.1097/PR9.0000000000000883 33981926 PMC8108585

[354] Kleinschnitz C Brinkhoff J Zelenka M Sommer C Stoll G. The extent of cytokine induction in peripheral nerve lesions depends on the mode of injury and NMDA receptor signaling. J Neuroimmunol. (2004) 149(1–2):77–83. 10.1016/j.jneuroim.2003.12.013 15020067

[355] Chun S Kwon YB. The CCL2 elevation in primary afferent fibers produces zymosan-induced hyperalgesia through microglia-mediated neuronal activation in the spinal dorsal horn. Brain Res Bull. (2019) 149:53–9. 10.1016/j.brainresbull.2019.04.014 31005664

[356] Hamed EA Mohamed Farghaly HS Abdel Mola AF Fahmi MK Makhlouf MM Balfas MA. Role of monocyte chemoattractant protein-1, stromal derived factor-1 and retinoic acid in pathophysiology of neuropathic pain in rats. J Basic Clin Physiol Pharmacol. (2016) 27(4):411–24. 10.1515/jbcpp-2015-0105 26974138

[357] Sacerdote P Franchi S Trovato AE Valsecchi AE Panerai AE Colleoni M. Transient early expression of TNF-alpha in sciatic nerve and dorsal root ganglia in a mouse model of painful peripheral neuropathy. Neurosci Lett. (2008) 436(2):210–3. 10.1016/j.neulet.2008.03.023 18394803

[358] Lee HL Lee KM Son SJ Hwang SH Cho HJ. Temporal expression of cytokines and their receptors mRNAs in a neuropathic pain model. Neuroreport. (2004) 15(18):2807–11. 15597059

[359] Woolf CJ. Evidence for a central component of post-injury pain hypersensitivity. Nature. (1983) 306(5944):686–8. 6656869 10.1038/306686a0

[360] Halievski K Ghazisaeidi S Salter MW. Sex-Dependent mechanisms of chronic pain: a focus on microglia and P2X4R. J Pharmacol Exp Ther. (2020) 375(1):202–9. 10.1124/jpet.120.265017 32114512

[361] Mapplebeck JC Beggs S Salter MW. Molecules in pain and sex: a developing story. Mol Brain. (2017) 10(1):9. 10.1186/s13041-017-0289-8 28270169 PMC5341415

[362] Sorge RE Mapplebeck JC Rosen S Beggs S Taves S Alexander JK Different immune cells mediate mechanical pain hypersensitivity in male and female mice. Nat Neurosci. (2015) 18(8):1081–3. 10.1038/nn.4053 26120961 PMC4772157

[363] Mapplebeck JCS Lorenzo LE Lee KY Gauthier C Muley MM De KY Chloride dysregulation through downregulation of KCC2 mediates neuropathic pain in both sexes. Cell Rep. (2019) 28(3):590–6. 10.1016/j.celrep.2019.06.059 31315039

[364] Mapplebeck JCS Dalgarno R Tu Y Moriarty O Beggs S Kwok CHT Microglial P2X4R-evoked pain hypersensitivity is sexually dimorphic in rats. Pain. (2018) 159(9):1752–63. 10.1097/j.pain.0000000000001265 29927790

[365] Mogil JS. Sex differences in pain and pain inhibition: multiple explanations of a controversial phenomenon. Nat Rev Neurosci. (2012) 13(12):859–66. 10.1038/nrn3360 23165262

[366] Mogil JS. Qualitative sex differences in pain processing: emerging evidence of a biased literature. Nat Rev Neurosci. (2020) 21(7):353–65. 10.1038/s41583-020-0310-6 32440016

[367] Mifflin KA Frieser E Benson C Baker G Kerr BJ. Voluntary wheel running differentially affects disease outcomes in male and female mice with experimental autoimmune encephalomyelitis. J Neuroimmunol. (2017) 305:135–44. 10.1016/j.jneuroim.2017.02.005 28284334

[368] Luo X Chen O Wang Z Bang S Ji J Lee SH IL-23/IL-17A/TRPV1 axis produces mechanical pain via macrophage-sensory neuron crosstalk in female mice. Neuron. (2021) 109:2691–706. 10.1016/j.neuron.2021.06.015 34473953 PMC8425601

[369] Paige C Barba-Escobedo PA Mecklenburg J Patil M Goffin V Grattan DR Neuroendocrine mechanisms governing sex differences in hyperalgesic priming involve prolactin receptor sensory neuron signaling. J Neurosci. (2020) 40(37):7080–90. 10.1523/JNEUROSCI.1499-20.2020 32801151 PMC7480243

[370] Lim H Lee H Noh K Lee SJ. IKK/NF-kappaB-dependent satellite glia activation induces spinal cord microglia activation and neuropathic pain after nerve injury. Pain. (2017) 158(9):1666–77. 10.1097/j.pain.0000000000000959 28722693

[371] Gushchina S Pryce G Yip PK Wu D Pallier P Giovannoni G Increased expression of colony-stimulating factor-1 in mouse spinal cord with experimental autoimmune encephalomyelitis correlates with microglial activation and neuronal loss. Glia. (2018) 66(10):2108–25. 10.1002/glia.23464 30144320

[372] Lee J Hwang H Lee SJ. Distinct roles of GT1b and CSF-1 in microglia activation in nerve injury-induced neuropathic pain. Mol Pain. (2021) 17:17448069211020918. 10.1177/17448069211020918 34056970 PMC8168050

[373] Sun C Tao X Wan C Zhang X Zhao M Xu M Spinal cord stimulation alleviates neuropathic pain by attenuating microglial activation via reducing colony-stimulating factor 1 levels in the spinal cord in a rat model of chronic constriction injury. Anesth Analg. (2022) 135(1):178–90. 10.1213/ANE.0000000000006016 35709447 PMC9172898

[374] Kuhn JA Vainchtein ID Braz J Hamel K Bernstein M Craik V Regulatory T-cells inhibit microglia-induced pain hypersensitivity in female mice. Elife. (2021) 10:e69056. 10.7554/eLife.69056 34652270 PMC8639143

[375] Beggs S Trang T Salter MW. P2x4r+ microglia drive neuropathic pain. Nat Neurosci. (2012) 15(8):1068–73. 10.1038/nn.3155 22837036 PMC5023423

[376] Tam TH Salter MW. Purinergic signalling in spinal pain processing. Purinergic Signal. (2021) 17(1):49–54. 10.1007/s11302-020-09748-5 33169292 PMC7954904

[377] Trang T Beggs S Salter MW. ATP receptors gate microglia signaling in neuropathic pain. Exp Neurol. (2012) 234(2):354–61. 10.1016/j.expneurol.2011.11.012 22116040 PMC3748033

[378] Trang T Salter MW. P2x4 purinoceptor signaling in chronic pain. Purinergic Signal. (2012) 8(3):621–8. 10.1007/s11302-012-9306-7 22528681 PMC3360095

[379] Tsuda M Shigemoto-Mogami Y Koizumi S Mizokoshi A Kohsaka S Salter MW P2x(4) receptors induced in spinal microglia gate tactile allodynia after nerve injury. Nature. (2003) 424:778–83. 10.1038/nature01786 12917686

[380] Malcangio M. Spinal mechanisms of neuropathic pain: is there a P2X4-BDNF controversy? Neurobiol Pain. (2017) 1:1–5. 10.1016/j.ynpai.2017.04.001 30272037 PMC6148335

[381] Sorge RE Totsch SK. Sex differences in pain. J Neurosci Res. (2016) 95:1271–81. 10.1002/jnr.23841 27452349

[382] Luo X Tai WL Sun L Pan Z Xia Z Chung SK Crosstalk between astrocytic CXCL12 and microglial CXCR4 contributes to the development of neuropathic pain. Mol Pain. (2016) 12:1744806916636385. 10.1177/1744806916636385 27030717 PMC4956184

[383] Old EA Malcangio M. Chemokine mediated neuron-glia communication and aberrant signalling in neuropathic pain states. Curr Opin Pharmacol. (2012) 12(1):67–73. 10.1016/j.coph.2011.10.015 22056024

[384] Liu ZY Song ZW Guo SW He JS Wang SY Zhu JG CXCL12/CXCR4 signaling contributes to neuropathic pain via central sensitization mechanisms in a rat spinal nerve ligation model. CNS Neurosci Ther. (2019) 25(9):922–36. 10.1111/cns.13128 30955244 PMC6698967

[385] Dong J Xu C Xia R Zhang Z. Upregulating miR-130a-5p relieves astrocyte over activation-induced neuropathic pain through targeting C-X-C motif chemokine receptor 12/C-X-C motif chemokine receptor 4 axis. Neuroreport. (2021) 32(2):135–43. 10.1097/WNR.0000000000001573 33395188

[386] Yang F Sun W Luo WJ Yang Y Yang F Wang XL SDF1-CXCR4 signaling contributes to the transition from acute to chronic pain state. Mol Neurobiol. (2017) 54(4):2763–75. 10.1007/s12035-016-9875-5 27011380

[387] Hendrich J Alvarez P Joseph EK Chen X Bogen O Levine JD. Electrophysiological correlates of hyperalgesic priming in vitro and in vivo. Pain. (2013) 154(10):2207–15. 10.1016/j.pain.2013.07.004 23831864 PMC3838101

[388] Ferrari LF Bogen O Levine JD. Nociceptor subpopulations involved in hyperalgesic priming. Neuroscienc. (2010) 165(3):896–901. 10.1016/j.neuroscience.2009.11.029 PMC2815163 19931357

[389] Honjoh K Nakajima H Hirai T Watanabe S Matsumine A. Relationship of inflammatory cytokines from M1-type microglia/macrophages at the injured site and lumbar enlargement with neuropathic pain after spinal cord injury in the CCL21 knockout (plt) mouse. Front Cell Neurosci. (2019) 13:525. 10.3389/fncel.2019.00525 31824269 PMC6881269

[390] de Jong EK Vinet J Stanulovic VS Meijer M Wesseling E Sjollema K Expression, transport, and axonal sorting of neuronal CCL21 in large dense-core vesicles. FASEB J. (2008) 22(12):4136–45. 10.1096/fj.07-101907 18697841

[391] de Jong EK Dijkstra IM Hensens M Brouwer N van Amerongen M Liem RS Vesicle-mediated transport and release of CCL21 in endangered neurons: a possible explanation for microglia activation remote from a primary lesion. J Neurosci. (2005) 25(33):7548–57. 10.1523/JNEUROSCI.1019-05.2005 16107642 PMC6725403

[392] van Weering HR de Jong AP de Haas AH Biber KP Boddeke HW. CCL21-induced Calcium transients and proliferation in primary mouse astrocytes: cXCR3-dependent and independent responses. Brain Behav Immun. (2010) 24(5):768–75. 10.1016/j.bbi.2009.04.007 19401230

[393] Andratsch M Mair N Constantin CE Scherbakov N Benetti C Quarta S A key role for gp130 expressed on peripheral sensory nerves in pathological pain. J Neurosci. (2009) 29(43):13473–83. 10.1523/JNEUROSCI.1822-09.2009 19864560 PMC6664994

[394] Recasens M Almolda B Perez-Clausell J Campbell IL Gonzalez B Castellano B. Chronic exposure to IL-6 induces a desensitized phenotype of the microglia. J Neuroinflammation. (2021) 18(1):31. 10.1186/s12974-020-02063-1 33482848 PMC7821504

[395] Tsuda M Masuda T Kitano J Shimoyama H Tozaki-Saitoh H Inoue K. IFN-gamma receptor signaling mediates spinal microglia activation driving neuropathic pain. Proc Natl Acad Sci U S A. (2009) 106(19):8032–7. 10.1073/pnas.0810420106 19380717 PMC2683100

[396] Vikman KS Siddall PJ Duggan AW. Increased responsiveness of rat dorsal horn neurons in vivo following prolonged intrathecal exposure to interferon-[gamma]. Neuroscience. (2005) 135(3):969–77. 10.1016/j.neuroscience.2005.06.059 16125859

[397] Vikman KS Hill RH Backstrom E Robertson B Kristensson K. Interferon-gamma induces characteristics of central sensitization in spinal dorsal horn neurons in vitro. Pain. (2003) 106(3):241–51. 10.1016/S0304-3959(03)00262-8 14659507

[398] Neumann H Schmidt H Wilharm E Behrens L Wekerle H. Interferon gamma gene expression in sensory neurons: evidence for autocrine gene regulation. J Exp Med. (1997) 186(12):2023–31. 10.1084/jem.186.12.2023 9396771 PMC2199162

[399] Costigan M Moss A Latremoliere A Johnston C Verma-Gandhu M Herbert TA T-cell infiltration and signaling in the adult dorsal spinal cord is a major contributor to neuropathic pain-like hypersensitivity. J Neurosci. (2009) 29(46):14415–22. 10.1523/JNEUROSCI.4569-09.2009 19923276 PMC2813708

[400] Clark AK Wodarski R Guida F Sasso O Malcangio M. Cathepsin S release from primary cultured microglia is regulated by the P2X7 receptor. Glia. (2010) 58(14):1710–26. 10.1002/glia.21042 20629190

[401] Clark AK Yip PK Malcangio M. The liberation of fractalkine in the dorsal horn requires microglial cathepsin S. J Neurosci. (2009) 29(21):6945–54. 10.1523/JNEUROSCI.0828-09.2009 19474321 PMC2698289

[402] Verge GM Milligan ED Maier SF Watkins LR Naeve GS Foster AC. Fractalkine (CX3CL1) and fractalkine receptor (CX3CR1) distribution in spinal cord and dorsal root ganglia under basal and neuropathic pain conditions. Eur J Neurosci. (2004) 20(5):1150–60. 10.1111/j.1460-9568.2004.03593.x 15341587

[403] Lindia JA McGowan E Jochnowitz N Abbadie C. Induction of CX3CL1 expression in astrocytes and CX3CR1 in microglia in the spinal cord of a rat model of neuropathic pain. J Pain. (2005) 6(7):434–8. 10.1016/j.jpain.2005.02.001 15993821

[404] Milligan ED Zapata V Chacur M Schoeniger D Biedenkapp J O’connor KA Evidence that exogenous and endogenous fractalkine can induce spinal nociceptive facilitation in rats. Eur J Neurosci. (2004) 20(9):2294–302. 10.1111/j.1460-9568.2004.03709.x 15525271

[405] Staniland AA Clark AK Wodarski R Sasso O Maione F D’Acquisto F Reduced inflammatory and neuropathic pain and decreased spinal microglial response in fractalkine receptor (CX3CR1) knockout mice. J Neurochem. (2010) 114(4):1143–57. 10.1111/j.1471-4159.2010.06837.x 20524966

[406] Clark AK Yip PK Grist J Gentry C Staniland AA Marchand F Inhibition of spinal microglial cathepsin S for the reversal of neuropathic pain. Proc Natl Acad Sci U S A. (2007) 104(25):10655–60. 10.1073/pnas.0610811104 17551020 PMC1965568

[407] Clark AK Gruber-Schoffnegger D Drdla-Schutting R Gerhold KJ Malcangio M Sandkuhler J. Selective activation of microglia facilitates synaptic strength. J Neurosci. (2015) 35(11):4552–70. 10.1523/JNEUROSCI.2061-14.2015 25788673 PMC4363384

[408] Biber K Laurie DJ Berthele A Sommer B Tolle TR Gebicke-Harter PJ Expression and signaling of group I metabotropic glutamate receptors in astrocytes and microglia. J Neurochem. (1999) 72(4):1671–80. 10.1046/j.1471-4159.1999.721671.x 10098876

[409] Byrnes KR Loane DJ Faden AI. Metabotropic glutamate receptors as targets for multipotential treatment of neurological disorders. Neurotherapeutics. (2009) 6(1):94–107. 10.1016/j.nurt.2008.10.038 19110202 PMC2634659

[410] Byrnes KR Stoica B Loane DJ Riccio A Davis MI Faden AI. Metabotropic glutamate receptor 5 activation inhibits microglial associated inflammation and neurotoxicity. Glia. (2009) 57(5):550–60. 10.1002/glia.20783 18816644 PMC2644739

[411] Byrnes KR Stoica B Riccio A Pajoohesh-Ganji A Loane DJ Faden AI. Activation of metabotropic glutamate receptor 5 improves recovery after spinal cord injury in rodents. Ann Neurol. (2009) 66(1):63–74. 10.1002/ana.21673 19670441 PMC3755738

[412] Devaraju P Sun MY Myers TL Lauderdale K Fiacco TA. Astrocytic group I mGluR-dependent potentiation of astrocytic glutamate and potassium uptake. J Neurophysiol. (2013) 109(9):2404–14. 10.1152/jn.00517.2012 23427307

[413] Yang G Tan Q Li Z Liu K Wu J Ye W The AMPK pathway triggers autophagy during CSF1-induced microglial activation and may be implicated in inducing neuropathic pain. J Neuroimmunol. (2020) 345:577261. 10.1016/j.jneuroim.2020.577261 32570135

[414] Lu VB Ballanyi K Colmers WF Smith PA. Neuron type-specific effects of brain-derived neurotrophic factor in rat superficial dorsal horn and their relevance to “central sensitization”. J Physiol. (2007) 584:543–63. 10.1113/jphysiol.2007.141267 17761774 PMC2277149

[415] Ha SO Kim JK Hong HS Kim DS Cho HJ. Expression of brain-derived neurotrophic factor in rat dorsal root ganglia, spinal cord and gracile nuclei in experimental models of neuropathic pain. Neuroscience. (2001) 107(2):301–9. 10.1016/S0306-4522(01)00353-0 11731104

[416] Miletic G Miletic V. Increases in the concentration of brain derived neurotrophic factor in the lumbar spinal dorsal horn are associated with pain behavior following chronic constriction injury in rats. Neurosci Lett. (2002) 319(3):137–40. 10.1016/S0304-3940(01)02576-9 11834312

[417] Garraway SM Petruska JC Mendell LM. BDNF sensitizes the response of lamina II neurons to high threshold primary afferent inputs. Eur J Neurosci. (2003) 18(9):2467–76. 10.1046/j.1460-9568.2003.02982.x 14622147

[418] Bardoni R Ghirri A Salio C Prandini M Merighi A. BDNF-mediated modulation of GABA and glycine release in dorsal horn lamina II from postnatal rats. Dev Neurobiol. (2007) 67(7):960–75. 10.1002/dneu.20401 17506495

[419] Ferrini F De Koninck Y. Microglia control neuronal network excitability via BDNF signalling. Neural Plast. (2013) 2013:429815. 10.1155/2013/429815 24089642 PMC3780625

[420] Zhang W Shi Y Peng Y Zhong L Zhu S Zhang W Neuron activity-induced wnt signaling up-regulates expression of brain-derived neurotrophic factor in the pain neural circuit. J Biol Chem. (2018) 293(40):15641–51. 10.1074/jbc.RA118.002840 30139740 PMC6177598

[421] Biggs JE Boakye PA Ganesan N Stemkowski PL Lantero A Ballanyi K Analysis of the long-term actions of gabapentin and pregabalin in dorsal root ganglia and substantia gelatinosa. J Neurophysiol. (2014) 112(10):2398–412. 10.1152/jn.00168.2014 25122705

[422] Huang D Yang J Liu X He L Luo X Tian H P2y6 receptor activation is involved in the development of neuropathic pain induced by chronic constriction injury of the sciatic nerve in rats. J Clin Neurosci. (2018) 56:156–62. 10.1016/j.jocn.2018.07.013 30045810

[423] Zhang X Li G. P2y receptors in neuropathic pain. Pharmacol Biochem Behav. (2019) 186:172788. 10.1016/j.pbb.2019.172788 31494119

[424] Inoue K Koizumi S Tsuda M. The role of nucleotides in the neuron–glia communication responsible for the brain functions. J Neurochem. (2007) 102(5):1447–58. 10.1111/j.1471-4159.2007.04824.x 17697046

[425] Barragan-Iglesias P Pineda-Farias JB Cervantes-Duran C Bravo-Hernandez M Rocha-Gonzalez HI Murbartian J Role of spinal P2Y6 and P2Y11 receptors in neuropathic pain in rats: possible involvement of glial cells. Mol Pain. (2014) 10:29. 10.1186/1744-8069-10-29 24886406 PMC4039548

[426] Kobayashi K Yamanaka H Fukuoka T Dai Y Obata K Noguchi K. P2y12 receptor upregulation in activated microglia is a gateway of p38 signaling and neuropathic pain. J Neurosci. (2008) 28(11):2892–902. 10.1523/JNEUROSCI.5589-07.2008 18337420 PMC6670682

[427] Kobayashi K Yamanaka H Yanamoto F Okubo M Noguchi K. Multiple P2Y subtypes in spinal microglia are involved in neuropathic pain after peripheral nerve injury. Glia. (2012) 60(10):1529–39. 10.1002/glia.22373 22736439

[428] Masuda T Ozono Y Mikuriya S Kohro Y Tozaki-Saitoh H Iwatsuki K Dorsal horn neurons release extracellular ATP in a VNUT-dependent manner that underlies neuropathic pain. Nat Commun. (2016) 7:12529. 10.1038/ncomms12529 27515581 PMC4990655

[429] Chu J Yang J Zhou Y Chen J Chen KH Zhang C ATP-releasing SWELL1 channel in spinal microglia contributes to neuropathic pain. Sci Adv. (2023) 9(13):eade9931. 10.1126/sciadv.ade9931 36989353 PMC10058245

[430] Shi Y Shu J Gelman BB Lisinicchia JG Tang SJ. Wnt signaling in the pathogenesis of human HIV-associated pain syndromes. J Neuroimmune Pharmacol. (2013) 8(4):956–64. 10.1007/s11481-013-9474-4 23737037 PMC3743252

[431] Zhang YK Huang ZJ Liu S Liu YP Song AA Song XJ. WNT signaling underlies the pathogenesis of neuropathic pain in rodents. J Clin Invest. (2013) 123(5):2268–86. 10.1172/JCI65364 23585476 PMC3635721

[432] Wang HY Liu T Malbon CC. Structure-function analysis of frizzleds. Cell Signal. (2006) 18(7):934–41. 10.1016/j.cellsig.2005.12.008 16480852

[433] Huang J Bloe CB Zhou X Wu S Zhang W. The role of the spinal wnt signaling pathway in HIV-related neuropathic pain. Cell Mol Neurobiol. (2020) 40(7):1075–85. 10.1007/s10571-020-00805-6 32100186 PMC11448846

[434] Zhou X Tao L Zhao M Wu S Obeng E Wang D Wnt/beta-catenin signaling regulates brain-derived neurotrophic factor release from spinal microglia to mediate HIV1 gp120-induced neuropathic pain. Mol Pain. (2020) 16:1744806920922100. 10.1177/1744806920922100 32354292 PMC7227158

[435] Li Y Li B Wan X Zhang W Zhong L Tang SJ. NMDA Receptor activation stimulates transcription-independent rapid wnt5a protein synthesis via the MAPK signaling pathway. Mol Brain. (2012) 5:1. 10.1186/1756-6606-5-1 22217152 PMC3287101

[436] Chen J Park CS Tang SJ. Activity-dependent synaptic wnt release regulates hippocampal long term potentiation. J Biol Chem. (2006) 281(17):11910–6. 10.1074/jbc.M511920200 16501258

[437] Echeverry S Shi XQ Yang M Huang H Wu Y Lorenzo LE Spinal microglia are required for long-term maintenance of neuropathic pain. Pain. (2017) 158(9):1792–801. 10.1097/j.pain.0000000000000982 28746078

[438] Croll SD Chesnutt CR Rudge JS Acheson A Ryan TE Siuciak JA Co-infusion with a TrkB-fc receptor body carrier enhances BDNF distribution in the adult rat brain. Exp Neurol. (1998) 152(1):20–33. 10.1006/exnr.1998.6836 9682009

[439] Clark AK D’Aquisto F Gentry C Marchand F McMahon SB Malcangio M. Rapid co-release of interleukin 1beta and caspase 1 in spinal cord inflammation. J Neurochem. (2006) 99(3):868–80. 10.1111/j.1471-4159.2006.04126.x 16942597

[440] Gajtko A Bakk E Hegedus K Ducza L Hollo K. IL-1beta induced cytokine expression by spinal astrocytes can play a role in the maintenance of chronic inflammatory pain. Front Physiol. (2020) 11:543331. 10.3389/fphys.2020.543331 33304271 PMC7701125

[441] Clark AK Staniland AA Marchand F Kaan TKY McMahon SB Malcangio M. P2X7-dependent release of interleukin-1{beta} and nociception in the spinal cord following lipopolysaccharide. J Neurosci. (2010) 30(2):573–82. 10.1523/JNEUROSCI.3295-09.2010 20071520 PMC2880485

[442] Kohno K Tsuda M. Role of microglia and P2X4 receptors in chronic pain. Pain Rep. (2021) 6(1):e864. 10.1097/PR9.0000000000000864 33981920 PMC8108579

[443] Simeoli R Montague K Jones HR Castaldi L Chambers D Kelleher JH Exosomal cargo including microRNA regulates sensory neuron to macrophage communication after nerve trauma. Nat Commun. (2017) 8(1):1778. 10.1038/s41467-017-01841-5 29176651 PMC5701122

[444] D’Agnelli S Gerra MC Bignami E Arendt-Nielsen L. Exosomes as a new pain biomarker opportunity. Mol Pain. (2020) 16:1744806920957800. 10.1177/1744806920957800 32909507 PMC7493250

[445] Hori N Narita M Yamashita A Horiuchi H Hamada Y Kondo T Changes in the expression of IL-6-mediated MicroRNAs in the dorsal root ganglion under neuropathic pain in mice. Synapse. (2016) 70(8):317–24. 10.1002/syn.21902 26990296

[446] McDonald MK Tian Y Qureshi RA Gormley M Ertel A Gao R Functional significance of macrophage-derived exosomes in inflammation and pain. Pain. (2014) 155(8):1527–39. 10.1016/j.pain.2014.04.029 24792623 PMC4106699

[447] Yu X Abdul M Fan BQ Zhang L Lin X Wu Y The release of exosomes in the medial prefrontal cortex and nucleus accumbens brain regions of chronic constriction injury (CCI) model mice could elevate the pain sensation. Neurosci Lett. (2020) 723:134774. 10.1016/j.neulet.2020.134774 31981720

[448] Morteza BH Ahmadi S Tarighat F Rahbarghazi R Soleimanpour H. Interplay between exosomes and autophagy machinery in pain management: state of the art. Neurobiol Pain. (2022) 12:100095. 10.1016/j.ynpai.2022.100095 35720640 PMC9198378

[449] Alles SRA Nascimento F Lujan R Luiz AP Millet Q Bangash MA Sensory neuron-derived na(V)1.7 contributes to dorsal horn neuron excitability. Sci Adv. (2020) 6(8):eaax4568. 32128393 10.1126/sciadv.aax4568 PMC7030926

[450] Paolicelli RC Bergamini G Rajendran L. Cell-to-cell communication by extracellular vesicles: focus on microglia. Neuroscience. (2019) 405:148–57. 10.1016/j.neuroscience.2018.04.003 29660443

[451] Gosselin RD Meylan P Decosterd I. Extracellular microvesicles from astrocytes contain functional glutamate transporters: regulation by protein kinase C and cell activation. Front Cell Neurosci. (2013) 7:251. 10.3389/fncel.2013.00009 24368897 PMC3857901

[452] Zhang YU Ye G Zhao J Chen Y Kong L Sheng C Exosomes carried miR-181c-5p alleviates neuropathic pain in CCI rat models. An Acad Bras Cienc. (2022) 94(3):e20210564. 10.1590/0001-3765202220210564 35976364

[453] Zhang X Wang J Zhou Q Xu Y Pu S Wu J Brain-derived neurotrophic factor-activated astrocytes produce mechanical allodynia in neuropathic pain. Neuroscience. (2011) 199:452–60. 10.1016/j.neuroscience.2011.10.017 22044922

[454] Balasubramanyan S Stemkowski PL Stebbing MJ Smith PA. Sciatic chronic constriction injury produces cell-type specific changes in the electrophysiological properties of rat *Substantia Gelatinosa* neurons. J Neurophysiol. (2006) 96:579–90. 10.1152/jn.00087.2006 16611846

[455] Chen Y Balasubramanyan S Lai AY Todd KG Smith P.A. Effects of sciatic nerve axotomy on excitatory synaptic transmission in rat substantia Gelatinosa. J Neurophysiol. (2009) 102:3203–15. 10.1152/jn.00296.2009 19793881

[456] Leitner J Westerholz S Heinke B Forsthuber L Wunderbaldinger G Jager T Impaired excitatory drive to spinal GABAergic neurons of neuropathic mice. PLoS One. (2013) 8(8):e73370. 10.1371/journal.pone.0073370 24009748 PMC3751881

[457] Bae C Wang J Shim HS Tang SJ Chung JM La JH. Mitochondrial superoxide increases excitatory synaptic strength in spinal dorsal horn neurons of neuropathic mice. Mol Pain. (2018) 14:1744806918797032. 10.1177/1744806918797032 30152257 PMC6113735

[458] Coull JA Boudreau D Bachand K Prescott SA Nault F Sik A Trans-synaptic shift in anion gradient in spinal lamina I neurons as a mechanism of neuropathic pain. Nature. (2003) 424(6951):938–42. 10.1038/nature01868 12931188

[459] Ferrini F Perez-Sanchez J Ferland S Lorenzo LE Godin AG Plasencia-Fernandez I Differential chloride homeostasis in the spinal dorsal horn locally shapes synaptic metaplasticity and modality-specific sensitization. Nat Commun. (2020) 11(1):3935. 10.1038/s41467-020-17824-y 32769979 PMC7414850

[460] Prescott SA Sejnowski TJ de Koninck Y. Reduction of anion reversal potential subverts the inhibitory control of firing rate in spinal lamina I neurons: towards a biophysical basis for neuropathic pain. Mol Pain. (2006) 2:32. 10.1186/1744-8069-2-32 17040565 PMC1624821

[461] Lavertu G Côté S De Koninck Y. Enhancing K–cl co-transport restores normal spinothalamic sensory coding in a neuropathic pain model. Brain. (2014) 137(3):724–38. 10.1093/brain/awt334 24369380

[462] Yeo M Chen Y Jiang C Chen G Wang K Chandra S Repurposing cancer drugs identifies kenpaullone which ameliorates pathologic pain in preclinical models via normalization of inhibitory neurotransmission. Nat Commun. (2021) 12(1):6208. 10.1038/s41467-021-26270-3 34707084 PMC8551327

[463] Prescott SA de Koninck Y Sejnowski TJ. Biophysical basis for three distinct dynamical mechanisms of action potential initiation. PLoS Comput Biol. (2008) 4:e1000198. 10.1371/journal.pcbi.1000198 18846205 PMC2551735

[464] Todd AJ. Neuronal circuitry for pain processing in the dorsal horn. Nat Rev Neurosci. (2010) 11(12):823–36. 10.1038/nrn2947 21068766 PMC3277941

[465] Peirs C Seal RP. Neural circuits for pain: recent advances and current views. Science. (2016) 354(6312):578–84. 10.1126/science.aaf8933 27811268 PMC11327866

[466] Peirs C Dallel R Todd AJ. Recent advances in our understanding of the organization of dorsal horn neuron populations and their contribution to cutaneous mechanical allodynia. J Neural Transm (Vienna). (2020) 127(4):505–25. 10.1007/s00702-020-02159-1 32239353 PMC7148279

[467] Baba H Ji RR Kohno T Moore KA Ataka T Wakai A Removal of GABAergic inhibition facilitates polysynaptic A fiber-mediated excitatory transmission to the superficial spinal dorsal horn. Mol Cell Neurosci. (2003) 24(3):818–30. 10.1016/S1044-7431(03)00236-7 14664828

[468] Prescott SA Ma Q De KY. Normal and abnormal coding of somatosensory stimuli causing pain. Nat Neurosci. (2014) 17(2):183–91. 10.1038/nn.3629 24473266 PMC4079041

[469] Price TJ Prescott SA. Inhibitory regulation of the pain gate and how its failure causes pathological pain. Pain. (2015) 156(5):789–92. 10.1097/j.pain.0000000000000139 25719614 PMC4589225

[470] Aby F Lorenzo LE Grivet Z Bouali-Benazzouz R Martin H Valerio S Switch of serotonergic descending inhibition into facilitation by a spinal chloride imbalance in neuropathic pain. Sci Adv. (2022) 8(30):eabo0689. 10.1126/sciadv.abo0689 35895817 PMC9328683

[471] Kerr BJ Bradbury EJ Bennett DL Trivedi PM Dassan P French J Brain-Derived neurotrophic factor modulates nociceptive sensory inputs and NMDA-evoked responses in the rat spinal cord. J Neurosci. (1999) 19(12):5138–48. 10.1523/JNEUROSCI.19-12-05138.1999 10366647 PMC6782650

[472] Hildebrand ME Xu J Dedek A Li Y Sengar AS Beggs S Potentiation of synaptic GluN2B NMDAR currents by Fyn kinase is gated through BDNF-mediated disinhibition in spinal pain processing. Cell Rep. (2016) 17(10):2753–65. 10.1016/j.celrep.2016.11.024 27926876

[473] Dedek A Xu J Kandegedara CM Lorenzo LE Godin AG De KY Loss of STEP61 couples disinhibition to N-methyl-d-aspartate receptor potentiation in rodent and human spinal pain processing. Brain. (2019) 142(6):1535–46. 10.1093/brain/awz105 31135041 PMC6536915

[474] Lee KY Prescott SA. Chloride dysregulation and inhibitory receptor blockade yield equivalent disinhibition of spinal neurons yet are differentially reversed by carbonic anhydrase blockade. Pain. (2015) 156(12):2431–7. 10.1097/j.pain.0000000000000301 26186265

[475] Dedek A Xu J Lorenzo LE Godin AG Kandegedara CM Glavina G Sexual dimorphism in a neuronal mechanism of spinal hyperexcitability across rodent and human models of pathological pain. Brain. (2022) 145(3):1124–38. 10.1093/brain/awab408 35323848 PMC9050559

[476] Chen W Walwyn W Ennes HS Kim H McRoberts JA Marvizon JC. BDNF Released during neuropathic pain potentiates NMDA receptors in primary afferent terminals. Eur J Neurosci. (2014) 39(9):1439–54. 10.1111/ejn.12516 24611998 PMC4122572

[477] Yan X Jiang E Gao M Weng HR. Endogenous activation of presynaptic NMDA receptors enhances glutamate release from the primary afferents in the spinal dorsal horn in a rat model of neuropathic pain. J Physiol. (2013) 591(7):2001–19. 10.1113/jphysiol.2012.250522 23359671 PMC3624865

[478] Ding X Cai J Li S Liu XD Wan Y Xing GG. BDNF Contributes to the development of neuropathic pain by induction of spinal long-term potentiation via SHP2 associated GluN2B-containing NMDA receptors activation in rats with spinal nerve ligation. Neurobiol Dis. (2015) 73:428–51. 10.1016/j.nbd.2014.10.025 25447233

[479] Li S Cai J Feng ZB Jin ZR Liu BH Zhao HY BDNF Contributes to spinal long-term potentiation and mechanical hypersensitivity via fyn-mediated phosphorylation of NMDA receptor GluN2B subunit at tyrosine 1472 in rats following spinal nerve ligation. Neurochem Res. (2017) 42(10):2712–29. 10.1007/s11064-017-2274-0 28497343

[480] Alexander GM van Rijn MA van Hilten JJ Perreault MJ Schwartzman RJ. Changes in cerebrospinal fluid levels of pro-inflammatory cytokines in CRPS. Pain. (2005) 116(3):213–9. 10.1016/j.pain.2005.04.013 15964681

[481] Pineau I Lacroix S. Proinflammatory cytokine synthesis in the injured mouse spinal cord: multiphasic expression pattern and identification of the cell types involved. J Comp Neurol. (2007) 500(2):267–85. 10.1002/cne.21149 17111361

[482] Yan X Li F Maixner DW Yadav R Gao M Ali MW Interleukin-1beta released by microglia initiates the enhanced glutamatergic activity in the spinal dorsal horn during paclitaxel-associated acute pain syndrome. Glia. (2019) 67(3):482–97. 10.1002/glia.23557 30578561

[483] Yan X Weng HR. Endogenous interleukin-1beta in neuropathic rats enhances glutamate release from the primary afferents in the spinal dorsal horn through coupling with presynaptic N-methyl-D-aspartic acid receptors. J Biol Chem. (2013) 288(42):30544–57. 10.1074/jbc.M113.495465 24003233 PMC3798525

[484] Weng HR Chen JH Cata JP. Inhibition of glutamate uptake in the spinal cord induces hyperalgesia and increased responses of spinal dorsal horn neurons to peripheral afferent stimulation. Neuroscience. (2006) 138(4):1351–60. 10.1016/j.neuroscience.2005.11.061 16426766

[485] Weng HR Chen JH Pan ZZ Nie H. Glial glutamate transporter 1 regulates the spatial and temporal coding of glutamatergic synaptic transmission in spinal lamina II neurons. Neuroscience. (2007) 149(4):898–907. 10.1016/j.neuroscience.2007.07.063 17935889

[486] Miraucourt LS Peirs C Dallel R Voisin DL. Glycine inhibitory dysfunction turns touch into pain through astrocyte-derived D-serine. Pain. (2011) 152(6):1340–8. 10.1016/j.pain.2011.02.021 21392888

[487] Liu T Jiang CY Fujita T Luo SW Kumamoto E. Enhancement by interleukin-1beta of AMPA and NMDA receptor-mediated currents in adult rat spinal superficial dorsal horn neurons. Mol Pain. (2013) 9:16. 10.1186/1744-8069-9-16 23537341 PMC3622562

[488] Zhang H Nei H Dougherty PM. A p38 mitogen-activated protein kinase-dependent mechanism of disinhibition in spinal synaptic transmission induced by tumor necrosis factor-alpha. J Neurosci. (2010) 30(38):12844–55. 10.1523/JNEUROSCI.2437-10.2010 20861388 PMC2947110

[489] Zhang H Zhang H Dougherty PM. Dynamic effects of TNF-α on synaptic transmission in mice over time following sciatic nerve chronic constriction injury. J Neurophysiol. (2013) 110(7):1663–71. 10.1152/jn.01088.2012 23864372 PMC4042422

[490] Kronschlager MT Drdla-Schutting R Gassner M Honsek SD Teuchmann HL Sandkuhler J. Gliogenic LTP spreads widely in nociceptive pathways. Science. (2016) 354(6316):1144–8. 10.1126/science.aah5715 27934764 PMC6145441

[491] Bliss TV Collingridge GL Kaang BK Zhuo M. Synaptic plasticity in the anterior cingulate cortex in acute and chronic pain. Nat Rev Neurosci. (2016) 17(8):485–96. 10.1038/nrn.2016.68 27307118

[492] Gamal-Eltrabily M Martinez-Lorenzana G Gonzalez-Hernandez A Condes-Lara M. Cortical modulation of nociception. Neuroscience. (2021) 458:256–70. 10.1016/j.neuroscience.2021.01.001 33465410

[493] Sandy-Hindmarch O Bennett DL Wiberg A Furniss D Baskozos G Schmid AB. Systemic inflammatory markers in neuropathic pain, nerve injury, and recovery. Pain. (2022) 163(3):526–37. 10.1097/j.pain.0000000000002386 34224495 PMC7612369

[494] Zhang WT Sha WL Zhu Q Wu XB He C. Plasticity of neuronal excitability and synaptic balance in the anterior nucleus of paraventricular thalamus after nerve injury. Brain Res Bull. (2022) 188:1–10. 10.1016/j.brainresbull.2022.07.008 35850188

[495] Song ZH Song XJ Yang CL Cao P Mao Y Jin Y Up-regulation of microglial chemokine CXCL12 in anterior cingulate cortex mediates neuropathic pain in diabetic mice. Acta Pharmacol Sin. (2023) 44(7):1337–49. 10.1038/s41401-022-01046-7 36697977 PMC10310783

[496] Zhuo M. Contribution of synaptic plasticity in the insular cortex to chronic pain. Neuroscience. (2016) 338:220–9. 10.1016/j.neuroscience.2016.08.014 27530697

[497] Taylor AM Mehrabani S Liu S Taylor AJ Cahill CM. Topography of microglial activation in sensory- and affect-related brain regions in chronic pain. J Neurosci Res. (2016) (2017) 95(6):1330–5. 10.1002/jnr.23883 27574286 PMC5332533

[498] Fiore NT Austin PJ. Peripheral nerve injury triggers neuroinflammation in the medial prefrontal Cortex and ventral hippocampus in a subgroup of rats with coincident affective behavioural changes. Neuroscience. (2019) 416:147–67. 10.1016/j.neuroscience.2019.08.005 31401182

[499] Wu XB Zhu Q Gao YJ. CCL2/CCR2 contributes to the altered excitatory-inhibitory synaptic balance in the nucleus Accumbens shell following peripheral nerve injury-induced neuropathic pain. Neurosci Bull. (2021) 37(7):921–33. 10.1007/s12264-021-00697-6 34003466 PMC8275785

[500] Lu JS Yang L Chen J Xiong FF Cai P Wang XY Basolateral amygdala astrocytes modulate diabetic neuropathic pain and may be a potential therapeutic target for koumine. Br J Pharmacol. (2023) 180(10):1408–28. 10.1111/bph.16011 36519959

[501] Taylor AM Castonguay A Taylor AJ Murphy NP Ghogha A Cook C Microglia disrupt mesolimbic reward circuitry in chronic pain. J Neurosci. (2015) 35(22):8442–50. 10.1523/JNEUROSCI.4036-14.2015 26041913 PMC4452552

[502] Cordeiro MS Zhang Z Seguela P. Peripheral neuropathy induces HCN channel dysfunction in pyramidal neurons of the medial prefrontal Cortex. J Neurosci. (2015) 35(38):13244–56. 10.1523/JNEUROSCI.0799-15.2015 26400952 PMC6605438

[503] Ding W You Z Shen S Chen L Zhu S Mao J. Inhibition of HCN channel activity in the thalamus attenuates chronic pain in rats. Neurosci Lett. (2016) 631:97–103. 10.1016/j.neulet.2016.08.021 27542339 PMC6790005

[504] Yan Y Zhu M Cao X Xu G Shen W Li F Thalamocortical circuit controls neuropathic pain via up-regulation of HCN2 in the ventral posterolateral thalamus. Neurosci Bull. (2023) 39(5):774–92. 10.1007/s12264-022-00989-5 36538279 PMC10169982

[505] Hains BC Saab CY Waxman SG. Alterations in burst firing of thalamic VPL neurons and reversal by na(v)1.3 antisense after spinal cord injury. J Neurophysiol. (2006) 95(6):3343–52. 10.1152/jn.01009.2005 16481457

[506] Hains BC Saab CY Waxman SG. Changes in electrophysiological properties and sodium channel Nav1.3 expression in thalamic neurons after spinal cord injury. Brain. (2005) 128(Pt 10):2359–71. 10.1093/brain/awh623 16109750

[507] Bannister K Dickenson AH. The plasticity of descending controls in pain: translational probing. J Physiol. (2017) 595(13):4159–66. 10.1113/JP274165 28387936 PMC5491855

[508] Bannister K Patel R Goncalves L Townson L Dickenson AH. Diffuse noxious inhibitory controls and nerve injury: restoring an imbalance between descending monoamine inhibitions and facilitations. Pain. (2015) 156(9):1803–11. 10.1097/j.pain.0000000000000240 26010460

[509] Bannister K Dickenson AH. What do monoamines do in pain modulation? Curr Opin Support Palliat Care. (2016) 10(2):143–8. 10.1097/SPC.0000000000000207 27043287 PMC5604728

[510] Bannister K Dickenson AH. What the brain tells the spinal cord. Pain. (2016) 157(10):2148–51. 10.1097/j.pain.0000000000000568 27023423

[511] Bannister K Lockwood S Goncalves L Patel R Dickenson AH. An investigation into the inhibitory function of serotonin in diffuse noxious inhibitory controls in the neuropathic rat. Eur J Pain. (2017) 21(4):750–60. 10.1002/ejp.979 27891703

[512] Bannister K Qu C Navratilova E Oyarzo J Xie JY King T Multiple sites and actions of gabapentin-induced relief of ongoing experimental neuropathic pain. Pain. (2017) 158:2386–95. 10.1097/j.pain.0000000000001040 28832395 PMC5681862

[513] Frezel N Ranucci M Foster E Wende H Pelczar P Mendes R. c-Maf-positive spinal cord neurons are critical elements of a dorsal horn circuit for mechanical hypersensitivity in neuropathy. Cell Rep. (2023) 42(4):112295. 10.1016/j.celrep.2023.112295 36947543 PMC10157139

[514] Bee LA Dickenson AH. Rostral ventromedial medulla control of spinal sensory processing in normal and pathophysiological states. Neuroscience. (2007) 147(3):786–93. 10.1016/j.neuroscience.2007.05.004 17570596

[515] De Felice M Sanoja R Wang R Vera-Portocarrero L Oyarzo J King T Engagement of descending inhibition from the rostral ventromedial medulla protects against chronic neuropathic pain. Pain. (2011) 152(12):2701–9. 10.1016/j.pain.2011.06.008 21745713 PMC3222148

[516] Mitsi V Zachariou V. Modulation of pain, nociception, and analgesia by the brain reward center. Neuroscience. (2016) 338:81–92. 10.1016/j.neuroscience.2016.05.017 27189881 PMC5083150

[517] Ren W Centeno MV Berger S Wu Y Na X Liu X The indirect pathway of the nucleus accumbens shell amplifies neuropathic pain. Nat Neurosci. (2016) 19(2):220–2. 10.1038/nn.4199 26691834 PMC4889808

[518] Polgar E Hughes DI Riddell JS Maxwell DJ Puskar Z Todd AJ. Selective loss of spinal GABAergic or glycinergic neurons is not necessary for development of thermal hyperalgesia in the chronic constriction injury model of neuropathic pain. Pain. (2003) 104(1–2):229–39. 10.1016/S0304-3959(03)00011-3 12855333

[519] Polgar E Gray S Riddell JS Todd AJ. Lack of evidence for significant neuronal loss in laminae I-III of the spinal dorsal horn of the rat in the chronic constriction injury model. Pain. (2004) 111(1–2):144–50. 10.1016/j.pain.2004.06.011 15327818

[520] Webber CA Salame J Luu GL Acharjee S Ruangkittisakul A Martinez JA Nerve growth factor acts through the TrkA receptor to protect sensory neurons from the damaging effects of the HIV-1 viral protein, Vpr. Neuroscience. (2013) 252:512–25. 10.1016/j.neuroscience.2013.07.046 23912036 PMC3829629

[521] Peirs C Williams SG Zhao X Arokiaraj CM Ferreira DW Noh MC Mechanical allodynia circuitry in the dorsal horn is defined by the nature of the injury. Neuron. (2021) 109(1):73–90. 10.1016/j.neuron.2020.10.027 33181066 PMC7806207

[522] Polgar E Fowler JH McGill MM Todd AJ. The types of neuron which contain protein kinase C gamma in rat spinal cord. Brain Res. (1999) 833(1):71–80. 10.1016/S0006-8993(99)01500-0 10375678

[523] Smith KM Browne TJ Davis OC Coyle A Boyle KA Watanabe M Calretinin positive neurons form an excitatory amplifier network in the spinal cord dorsal horn. Elife. (2019) 8:e49190. 10.7554/eLife.49190 31713514 PMC6908433

[524] Malcangio M Tomlinson DR. A pharmacologic analysis of mechanical hyperalgesia in streptozotocin/diabetic rats. Pain. (1998) 76(1–2):151–7. 10.1016/S0304-3959(98)00037-2 9696468

[525] Yilmaz E Gold MS. Paclitaxel-induced increase in NCX activity in subpopulations of nociceptive afferents: a protective mechanism against chemotherapy-induced peripheral neuropathy? Cell Calcium. (2016) 60(1):25–31. 10.1016/j.ceca.2016.04.009 27166151 PMC4907840

[526] Idanpaan-Heikkila JJ Guilbaud G. Pharmacological studies on a rat model of trigeminal neuropathic pain: baclofen, but not carbamazepine, morphine or tricyclic antidepressants, attenuates the allodynia-like behaviour. Pain. (1999) 79(2–3):281–90. 10.1016/S0304-3959(98)00172-9 10068174

[527] Jain A Gyori BM Hakim S Bunga S Taub DG Ruiz-Cantero MC Nociceptor neuroimmune interactomes reveal cell type- and injury-specific inflammatory pain pathways. BioRxiv. (2023) 2023.02.01.526526. 10.1101/2023.02.01.526526. Preprint.

[528] Sorge RE Martin LJ Isbester KA Sotocinal SG Rosen S Tuttle AH Olfactory exposure to males, including men, causes stress and related analgesia in rodents. Nat Methods. (2014) 11(6):629–32. 10.1038/nmeth.2935 24776635

[529] Mogil JS. Animal models of pain: progress and challenges. Nat Rev Neurosci. (2009) 10(4):283–94. 10.1038/nrn2606 19259101

[530] Mogil JS Crager SE. What should we be measuring in behavioral studies of chronic pain in animals? Pain. (2004) 112:12–5. 10.1016/j.pain.2004.09.028 15494180

[531] Sexton JE Cox JJ Zhao J Wood JN. The genetics of pain: implications for therapeutics. Annu Rev Pharmacol Toxicol. (2017) 58:123–42. 10.1146/annurev-pharmtox-010617-052554 28968191

[532] Turner PV Pang DS Lofgren JL. A review of pain assessment methods in laboratory rodents. Comp Med. (2019) 69(6):451–67. 10.30802/AALAS-CM-19-000042 31896391 PMC6935698

[533] Sotocinal SG Sorge RE Zaloum A Tuttle AH Martin LJ Wieskopf JS The rat grimace scale: a partially automated method for quantifying pain in the laboratory rat via facial expressions. Mol Pain. (2011) 7:55. 10.1186/1744-8069-7-55 21801409 PMC3163602

[534] Diester CM Santos EJ Moerke MJ Negus SS. Behavioral battery for testing candidate analgesics in mice. I. validation with positive and negative controls. J Pharmacol Exp Ther. (2021) 377(2):232–41. 33622770 10.1124/jpet.120.000464 PMC8058504

[535] Diester CM Lichtman AH Negus SS. Behavioral battery for testing candidate analgesics in mice. II. Effects of Endocannabinoid Catabolic Enzyme Inhibitors and 9–Tetrahydrocannabinol. J Pharmacol Exp Ther. (2021) 377(2):242–53. 10.1124/jpet.120.000464 33622769 PMC8058502

[536] Andrade-Gonzalez RD Perrusquia-Hernandez E Montes-Angeles CD Castillo-Diaz LA Hernandez Campos ME Perez-Martinez IO. Encoding signs of orofacial neuropathic pain from facial expressions in mice. Arch Oral Biol. (2022) 135:105369. 10.1016/j.archoralbio.2022.105369 35149328

[537] Zylka MJ McCoy ES Park SK Patel RP Ryan DF Mullen ZJ Development and validation of painface, a software platform that simplifies and standardizes mouse grimace analyses. J Pain. (2023) 24(4, Supplement):35–6. 10.1016/j.jpain.2023.02.113

[538] Harte SE Meyers JB Donahue RR Taylor BK Morrow TJ. Mechanical conflict system: a novel operant method for the assessment of nociceptive behavior. PLoS One. (2016) 11(2):e0150164. 10.1371/journal.pone.0150164 26915030 PMC4767889

[539] Mauderli AP Acosta-Rua A Vierck CJ. An operant assay of thermal pain in conscious, unrestrained rats. J Neurosci Methods. (2000) 97(1):19–29. 10.1016/S0165-0270(00)00160-6 10771071

[540] Negus SS Vanderah TW Brandt MR Bilsky EJ Becerra L Borsook D. Preclinical assessment of candidate analgesic drugs: recent advances and future challenges. J Pharmacol Exp Ther. (2006) 319(2):507–14. 10.1124/jpet.106.106377 16751251

[541] Rostock C Schrenk-Siemens K Pohle J Siemens J. Human vs. Mouse nociceptors - similarities and differences. Neuroscience. (2018) 387:13–27. 10.1016/j.neuroscience.2017.11.047 29229553 PMC6150929

[542] Middleton SJ Barry AM Comini M Li Y Ray PR Shiers S Studying human nociceptors: from fundamentals to clinic. Brain. (2021) 144:1312–36. 10.1093/brain/awab048 34128530 PMC8219361

[543] Hartung JE Moy JK Loeza-Alcocer E Nagarajan V Jostock R Christoph T Voltage gated calcium channels in human dorsal root ganglion neurons. Pain. (2021) (2022) 163(6):e774–85. 10.1097/j.pain.0000000000002465 34510139 PMC8882208

[544] Shiers S Klein RM Price TJ. Quantitative differences in neuronal subpopulations between mouse and human dorsal root ganglia demonstrated with RNAscope in situ hybridization. Pain. (2020) 161(10):2410–24. 10.1097/j.pain.0000000000001973 32639368 PMC7899077

[545] Muizelaar JP Kleyer M Hertogs IA DeLange DC. Complex regional pain syndrome (reflex sympathetic dystrophy and causalgia): management with the calcium channel blocker nifedipine and/or the alpha-sympathetic blocker phenoxybenzamine in 59 patients. Clin Neurol Neurosurg. (1997) 99(1):26–30. 10.1016/S0303-8467(96)00594-X 9107464

[546] Cheng C Guo GF Martinez JA Singh V Zochodne DW. Dynamic plasticity of axons within a cutaneous milieu. J Neurosci. (2010) 30(44):14735–44. 10.1523/JNEUROSCI.2919-10.2010 21048132 PMC6633624

[547] Renthal W Chamessian A Curatolo M Davidson S Burton M Dib-Hajj S Human cells and networks of pain: transforming pain target identification and therapeutic development. Neuron. (2021) 109(9):1426–9. 10.1016/j.neuron.2021.04.005 33957072 PMC9208579

[548] McDermott LA Weir GA Themistocleous AC Segerdahl AR Blesneac I Baskozos G Defining the functional role of NaV1.7 in human nociception. Neuron. (2019) 101(5):905–19. 10.1016/j.neuron.2019.01.047 30795902 PMC6424805

[549] Meents JE Bressan E Sontag S Foerster A Hautvast P Rosseler C The role of Nav1.7 in human nociceptors: insights from human induced pluripotent stem cell-derived sensory neurons of erythromelalgia patients. Pain. (2019) 160(6):1327–41. 10.1097/j.pain.0000000000001511 30720580 PMC6554007

[550] Chambers SM Qi Y Mica Y Lee G Zhang XJ Niu L Combined small-molecule inhibition accelerates developmental timing and converts human pluripotent stem cells into nociceptors. Nat Biotechnol. (2012) 30(7):715–20. 10.1038/nbt.2249 22750882 PMC3516136

[551] Young GT Gutteridge A Fox H Wilbrey AL Cao L Cho LT Characterizing human stem cell-derived sensory neurons at the single-cell level reveals their ion channel expression and utility in pain research. Mol Ther. (2014) 22(8):1530–43. 10.1038/mt.2014.86 24832007 PMC4435594

[552] Lampert A Bennett DL McDermott LA Neureiter A Eberhardt E Winner B Human sensory neurons derived from pluripotent stem cells for disease modelling and personalized medicine. Neurobiol Pain. (2020) 8:100055. 10.1016/j.ynpai.2020.100055 33364527 PMC7750732

[553] Labau JIR Andelic M Faber CG Waxman SG Lauria G Dib-Hajj SD. Recent advances for using human induced-pluripotent stem cells as pain-in-a-dish models of neuropathic pain. Exp Neurol. (2022) 358:114223. 10.1016/j.expneurol.2022.114223 36100046

[554] Alsaloum M Labau JIR Liu S Effraim PR Waxman SG. Stem cell-derived sensory neurons modelling inherited erythromelalgia: normalization of excitability. Brain. (2023) 146(1):359–71. 10.1093/brain/awac031 35088838 PMC10060693

[555] Haile Y Nakhaei-Nejad M Boakye PA Baker G Smith PA Murray AG Reprogramming of HUVECs into induced pluripotent stem cells (HiPSCs), generation and characterization of HiPSC-derived neurons and astrocytes. PLoS One. (2015) 10(3):e0119617. 10.1371/journal.pone.0119617 25789622 PMC4366250

[556] Vojnits K Mahammad S Collins TJ Bhatia M. Chemotherapy-Induced neuropathy and drug discovery platform using human sensory neurons converted directly from adult peripheral blood. Stem Cells Transl Med. (2019) 8(11):1180–91. 10.1002/sctm.19-0054 31347791 PMC6811699

[557] Rodriguez-Palma EJ De la Luz-Cuellar YE Islas-Espinoza AM Felix-Leyva AE Shiers SI Garcia G Activation of alpha 6 -containing GABA A receptors induces antinociception under physiological and pathological conditions. Pain. (2023) 164(5):948–66. 10.1097/j.pain.0000000000002763 36001074 PMC9950299

[558] Backonja MM Attal N Baron R Bouhassira D Drangholt M Dyck PJ Value of quantitative sensory testing in neurological and pain disorders: neuPSIG consensus. Pain. (2013) 154(9):1807–19. 10.1016/j.pain.2013.05.047 23742795

[559] Vollert J Maier C Attal N Bennett DLH Bouhassira D Enax-Krumova EK Stratifying patients with peripheral neuropathic pain based on sensory profiles: algorithm and sample size recommendations. Pain. (2017) 158(8):1446–55. 10.1097/j.pain.0000000000000935 28595241 PMC5515640

[560] Rolke R Baron R Maier C Tolle TR Treede DR Beyer A Quantitative sensory testing in the German research network on neuropathic pain (DFNS): standardized protocol and reference values. Pain. (2006) 123(3):231–43. 10.1016/j.pain.2006.01.041 16697110

[561] Serra J Bostock H Sola R Aleu J Garcia E Cokic B Microneurographic identification of spontaneous activity in C-nociceptors in neuropathic pain states in humans and rats. Pain. (2012) 153(1):42–55. 10.1016/j.pain.2011.08.015 21993185

[562] Pinheiro ES de Queiros FC Montoya P Santos CL do Nascimento MA Ito CH Electroencephalographic Patterns in Chronic Pain: A Systematic Review of the Literature. PLoS One. (2016) 11(2):e0149085. 10.1371/journal.pone.0149085 26914356 PMC4767709

[563] Andelic M Salvi E Marcuzzo S Marchi M Lombardi R Cartelli D Integrative miRNA-mRNA profiling of human epidermis: unique signature of SCN9A painful neuropathy. Brain. (2023) (2023) 146(7):3049–62. 10.1093/brain/awad025 36730021 PMC10316770

[564] North RY Li Y Ray P Rhines LD Tatsui CE Rao G Electrophysiological and transcriptomic correlates of neuropathic pain in human dorsal root ganglion neurons. Brain. (2019) 142(5):1215–26. 10.1093/brain/awz063 30887021 PMC6487328

[565] Fillingim RB King CD Ribeiro-Dasilva MC Rahim-Williams B Riley JL III. Sex, gender, and pain: a review of recent clinical and experimental findings. J Pain. (2009) 10(5):447–85. 10.1016/j.jpain.2008.12.001 19411059 PMC2677686

[566] Martin LJ Acland EL Cho C Gandhi W Chen D Corley E Male-Specific conditioned pain hypersensitivity in mice and humans. Curr Biol. (2019) 29(2):192–201. 10.1016/j.cub.2018.11.030 30639112

[567] Hendrich J Alvarez P Joseph EK Ferrari LF Chen X Levine JD. In vivo and in vitro comparison of female and male nociceptors. J Pain. (2012) 13(12):1224–31. 10.1016/j.jpain.2012.09.009 23146406 PMC3508307

[568] Shansky RM Murphy AZ. Considering sex as a biological variable will require a global shift in science culture. Nat Neurosci. (2021) 24(4):457–64. 10.1038/s41593-021-00806-8 33649507 PMC12900283

[569] Mifflin KA Kerr BJ. Sex-related differences in acute and chronic pain: a bench to bedside perspective. Can J Anaesth. (2013) 60(3):221–6. 10.1007/s12630-012-9881-7 23288582

[570] Fauchon C Kim JA El-Sayed R Osborne NR Rogachov A Cheng JC Exploring sex differences in alpha brain activity as a potential neuromarker associated with neuropathic pain. Pain. (2022) 163(7):1291–302. 10.1097/j.pain.0000000000002491 34711764

[571] Johnston KJA Ward J Ray PR Adams MJ McIntosh AM Smith BH Sex-stratified genome-wide association study of multisite chronic pain in UK biobank. PLoS Genet. (2021) 17(4):e1009428. 10.1371/journal.pgen.1009428 33830993 PMC8031124

[572] Ovsepian SV Waxman SG. Gene therapy for chronic pain: emerging opportunities in target-rich peripheral nociceptors. Nat Rev Neurosci. (2023) 24(4):252–65. 10.1038/s41583-022-00673-7 36658346

[573] Moreno AM Aleman F Catroli GF Hunt M Hu M Dailamy A Long-lasting analgesia via targeted in situ repression of NaV1.7 in mice. Sci Transl Med. (2021) 13(584):eaay9056. 10.1126/scitranslmed.aay9056 33692134 PMC8830379

